# Integrated multi-omics identifies distinct immune signatures with diagnostic and therapeutic implications in immune checkpoint inhibitor-induced myocarditis

**DOI:** 10.1038/s41467-026-77262-0

**Published:** 2026-10-14

**Authors:** Shi-Long Zhang, Qing-Qing Cai, Jian Zhang, Yuan Liu, Hao Lu, Ye-Rui Zhang, Yu-Chen Xu, Hai-Xiang Peng, Hui-Yong Chen, Yan Wang, Lei-Lei Cheng, Jun-Bo Ge

**Affiliations:** 1 Department of Medical Oncology, Zhongshan Hospital, Fudan University, Shanghai, China; 2 Institute of Cardio-Oncology, Fudan University (in preparation), Shanghai, China; 3 Department of Pharmacy, Zhongshan Hospital, Fudan University, Shanghai, China; 4 Department of Cardiology, Zhongshan Hospital, Fudan University, Shanghai, China; 5 National Clinical Research Center for Interventional Medicine, Shanghai, China; 6 Shanghai Institute of Cardiovascular Diseases, Shanghai, China; 7 Department of Echocardiography, Zhongshan Hospital, Fudan University, Shanghai, China; 8 Shanghai Institute of Medical Imaging, Zhongshan Hospital, Fudan University, Shanghai, China; 9 Department of Cardiology, Shanghai Geriatrics Center, Shanghai, China; 10 Dunwill Med-Tech, Shanghai, China; 11 Department of Rheumatology and Immunology, Zhongshan Hospital, Fudan University, Shanghai, China

**Keywords:** Cancer immunotherapy, Cardiology, CD8-positive T cells, Chemokines, Monocytes and macrophages

## Abstract

Immune checkpoint inhibitor-associated myocarditis (ICI-myocarditis) is a rare but frequently fatal immune-related adverse event. A comprehensive understanding of the cellular and molecular determinants driving ICI-myocarditis is essential for improving diagnostic precision and developing targeted interventions. We delineate the immune landscape of ICI-myocarditis via scRNA-seq, flow cytometry, Olink proteomics, and multiplex cytokine profiling. We demonstrate that high levels of baseline ISG15⁺CD8⁺ T cells are linked to the proinflammatory features of monocytes and in vitro experiments confirm the existence of circulating ISG15⁺CD8⁺ T cells with a high potential to induce cardiomyocyte apoptosis, supporting their pathogenic potential for cardiomyocyte cytotoxicity. Notably, intercellular crosstalk between these subsets is possibly mediated via the CCL2-CCR2 axis, and its blockade attenuated ICI-induced cardiac injury in vivo. Based on the inflammatory signature in ICI-myocarditis, we develop an 8-protein custom panel with promising performance in distinguishing ICI-myocarditis from other ICIs-treated controls. JAK-STAT pathway activation promotes inflammatory programs in both ISG15⁺CD8⁺ T cells and monocytes. Furthermore, JAK-STAT inhibition ameliorates cardiac dysfunction and inflammatory infiltration while enhancing the response to anti-PD-1 therapy in mouse models. In summary, this study implicates immune-pathogenic features of ICI-myocarditis and provides a possible basis for future therapies.

## Abstract (web summary)

Immune checkpoint inhibitor (ICI)-induced myocarditis is a rare side effect of ICI therapy. Here the authors use multi-omics approaches to characterize the immune cells and mediators involved and find monocytes and ISG15^+^ CD8^+^ T cells as well as an 8-protein panel to better distinguish ICI-myocarditis.

## Introduction

Immune checkpoint inhibitors (ICIs) have revolutionized the treatment landscape across a wide spectrum of malignancies^1^. By reactivating antitumor T cell responses, ICIs substantially prolong survival in patients with advanced cancers^2^. However, this immune activation also predisposes patients to immune-related adverse events (irAEs), a spectrum of toxicities that can affect virtually any organ system. Among these, ICI-myocarditis, with an incidence of 0.27% to 1.14%, represents one of the most lethal irAEs and is associated with a mortality rate approaching 50%^3^. The clinical diagnosis of ICI-myocarditis remains challenging due to its heterogeneous presentation and overlap with cardiovascular or oncologic symptoms. Although cardiac MRI and endomyocardial biopsy, combining histopathology, immunohistochemistry, and pathogen detection, are recommended diagnostic tools, their limited use in routine practice often leads to delayed diagnosis and irreversible myocardial damage^4^. While corticosteroids remain the cornerstone of ICI-myocarditis therapy, steroid-refractory cases pose significant clinical challenges^5,6^. Therefore, the pathogenesis of ICI-myocarditis urgently needs to be elucidated to guide the treatment strategy without impeding antitumor efficacy.

The rise of single-cell omics allows detailed characterization of immune subsets and their disease-specific characteristics. Previous studies have implicated cytotoxic CD8⁺ T cells^7^, including α-myosin-specific clones^8^, and interferon-driven inflammatory macrophages^9^ in ICI-myocarditis pathophysiology. Yet, a comprehensive view of the systemic immune network, particularly the interplay between peripheral and cardiac compartments during disease onset and remission, remains elusive. Furthermore, no reliable biomarkers exist to predict which patients are at heightened risk for developing ICI-myocarditis^10^.

In this work, we integrate a comprehensive multi-omic profiling of circulating immune cells across ICI-myocarditis progression to delineate myocarditis-associated pathogenic subsets, upstream signals, and important immunopathological features. We identify a transcriptionally distinct population of ISG15⁺CD8⁺ T cells enriched during active disease and robust JAK-STAT signaling. ISG15⁺CD8⁺ T cells interacted with monocytes via the CCL2-CCR2 axis. Pharmacological blockade of the CCL2-CCR2 interaction alleviates ICI-myocarditis in mice. Based on these inflammatory signatures in ICI-myocarditis, we developed an 8-protein panel by constructing machine learning models integrating soluble and cellular biomarkers, which is able to distinguish ICI-myocarditis from other ICIs-treated controls and is validated in a larger independent cohort. Finally, leveraging bulk and single-cell RNA sequencing data from cardiac tissues, we link peripheral immune alterations to cardiac pathology and identify important biological processes underpinning ICI-myocarditis.

## Results

### Study design and patient enrollment

**Fig. 1:**
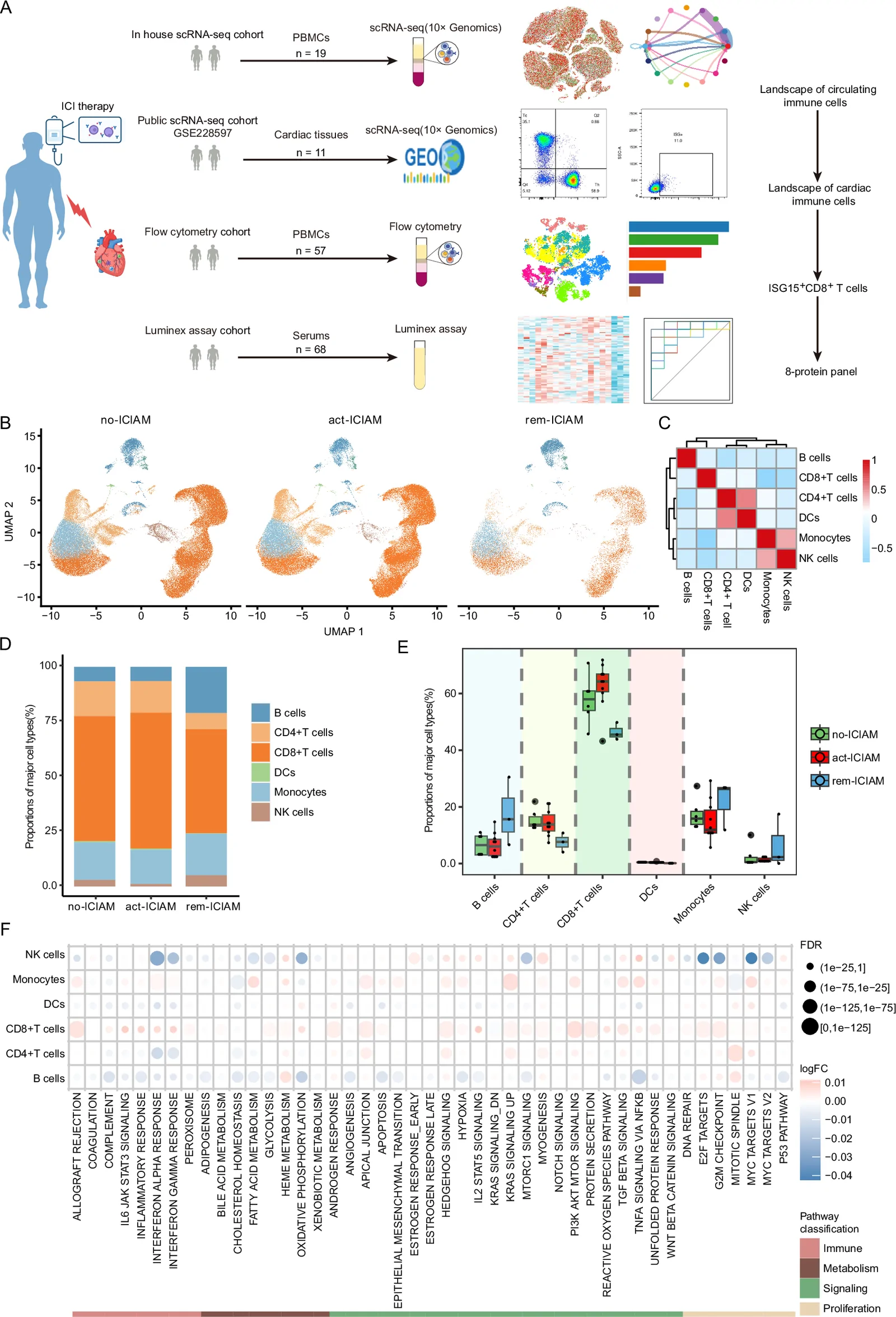
Overview of the patients included in the study and downstream analyses. **A** Workflow showing the collection and processing of samples for scRNA-seq, flow cytometry, Olink proteomics, and multiplex cytokine assays. **B** Uniform manifold approximation and projection (UMAP) view of individual cell clusters from peripheral blood mononuclear cells (PBMCs) of each group. **C** Heatmap showing the relationship among the six cell types based on the correlation of gene expression between each pair of cell types as assessed by Pearson’s correlation analysis. **D** Cell type frequency in each group. **E** Relative proportions of the main cell types between no-ICIAM (*n* = 6), act-ICIAM (*n* = 10), and rem-ICIAM (*n* = 3) samples. **F** Dot plots showing differentially enriched pathways in the main cell types between no-ICIAM and act-ICIAM samples. ICI, immune checkpoint inhibitor; no-ICIAM, no ICI-myocarditis; act-ICIAM, active ICI-myocarditis; rem-ICIAM, remitted ICI-myocarditis; UMAP, uniform manifold approximation and projection. Box plots depicted the interquartile range (box), median (center line), and full data range (whiskers). Mean ± SD were shown where appropriate.

We screened 19,676 patients treated with ICIs at our institution between September 30, 2017, and December 31, 2024. Among them, patients who developed ICI-myocarditis were enrolled in this study, while ICI-treated cancer patients without ICI-myocarditis were selected as matched controls. To identify candidate biomarkers and uncover pathophysiologic mechanisms of the disease, we conducted a comprehensive multi-omics profiling of peripheral blood, including scRNA-seq, Olink proteomics, flow cytometry, and multiplex cytokine assays (Fig. 1A). Biospecimens were obtained before corticosteroid administration. Owing to limited sample availability and differing material requirements across platforms, not all assays were performed for each patient. Therefore, samples were allocated into assay-specific analytical cohorts: a single-cell transcriptomic cohort comprising an in-house scRNA-seq cohort (*n* = 19, Supplementary Table 1) and a publicly available scRNA-seq cohort (*n* = 11), a flow cytometry cohort (*n* = 57, Supplementary Table 2), and a Luminex assay cohort (*n* = 68, Supplementary Table 3). All the demographic characteristics, clinical features, and types of immunotherapy were comparable between the groups within each cohort. To extend and cross-validate the findings derived from the in-house single-cell dataset, we further integrated an independent publicly available scRNA-seq dataset (GSE228597)^11^ of cardiac tissues.

### Global analysis of immune cell populations across no-ICIAM, act-ICIAM, and rem-ICIAM groups

To comprehensively elucidate immune responses and molecular alterations in ICI-myocarditis, particularly in active ICI-myocarditis (act-ICIAM), we analyzed peripheral blood mononuclear cells (PBMCs) from ICIs-treated cancer patients without ICI-myocarditis (no-ICIAM, *n* = 6), with active ICI-myocarditis (act-ICIAM, *n* = 10), and those in disease remission (rem-ICIAM, *n* = 3) in the in-house single-cell cohort (Supplementary Table 1). After quality control, 199,741 high-quality PBMCs were obtained (Supplementary Fig. 1A, B). Each sample contributed representative cells to all cellular subsets, suggesting minimal batch effects (Supplementary Fig. 1C, D). Based on established cell marker genes (Supplementary Table 4)^12^, we identified six major cell types, namely, NK cells, CD8^+^ T cells, CD4^+^ T cells, B cells, monocytes, and dendritic cells (DCs) (Fig. 1B and Supplementary Fig. 1E, F). A cell type-to-cell type correlation matrix demonstrated that each annotated cellular phenotype was mutually exclusive, highlighting distinct expression patterns associated with each cellular phenotype (Fig. 1C). Striking disparities in immune cell populations were observed across no-ICIAM, act-ICIAM, and rem-ICIAM groups, and CD8^+^ T cells were the largest cell population, followed by monocytes, CD4^+^ T cells, and other cell types (Fig. 1D and Supplementary Fig. 1G). Compared with no-ICIAM, act-ICIAM showed a significant increase in the proportion of CD8^+^ T cells and a marked decrease in monocytes (Fig. 1E), consistent with a recent study^13^. In contrast, B cells and monocytes were more enriched in the rem-ICIAM patients than in the other two groups. These results showed that act-ICIAM was associated with major changes in CD8^+^ T cells and monocytes, whereas other immune cell populations remained largely unchanged.

To further investigate functional alterations across major cell types, we used hallmark gene sets to compare pathway activities between the no-ICIAM and act-ICIAM groups. Notably, CD8^+^ T cells and monocytes showed upregulation across a broad spectrum of pathway activities, including different aspects of immunology, metabolism, signaling, and proliferation (Fig. 1F), implying that distinct anatomical contexts may preferentially remodel these cells and induce specific functional states. Furthermore, inflammatory response and IFN-related pathways were upregulated in CD8^+^ T cells, whereas monocytes showed greater enrichment of metabolism-associated pathways compared with the no-ICIAM group (Fig. 1F). In addition, CD4⁺ T cells, B cells, NK cells, and DCs displayed minimal changes in pathway activity.

### Identification of an imprinted peripheral CD8^+^ T cell compartment

**Fig. 2:**
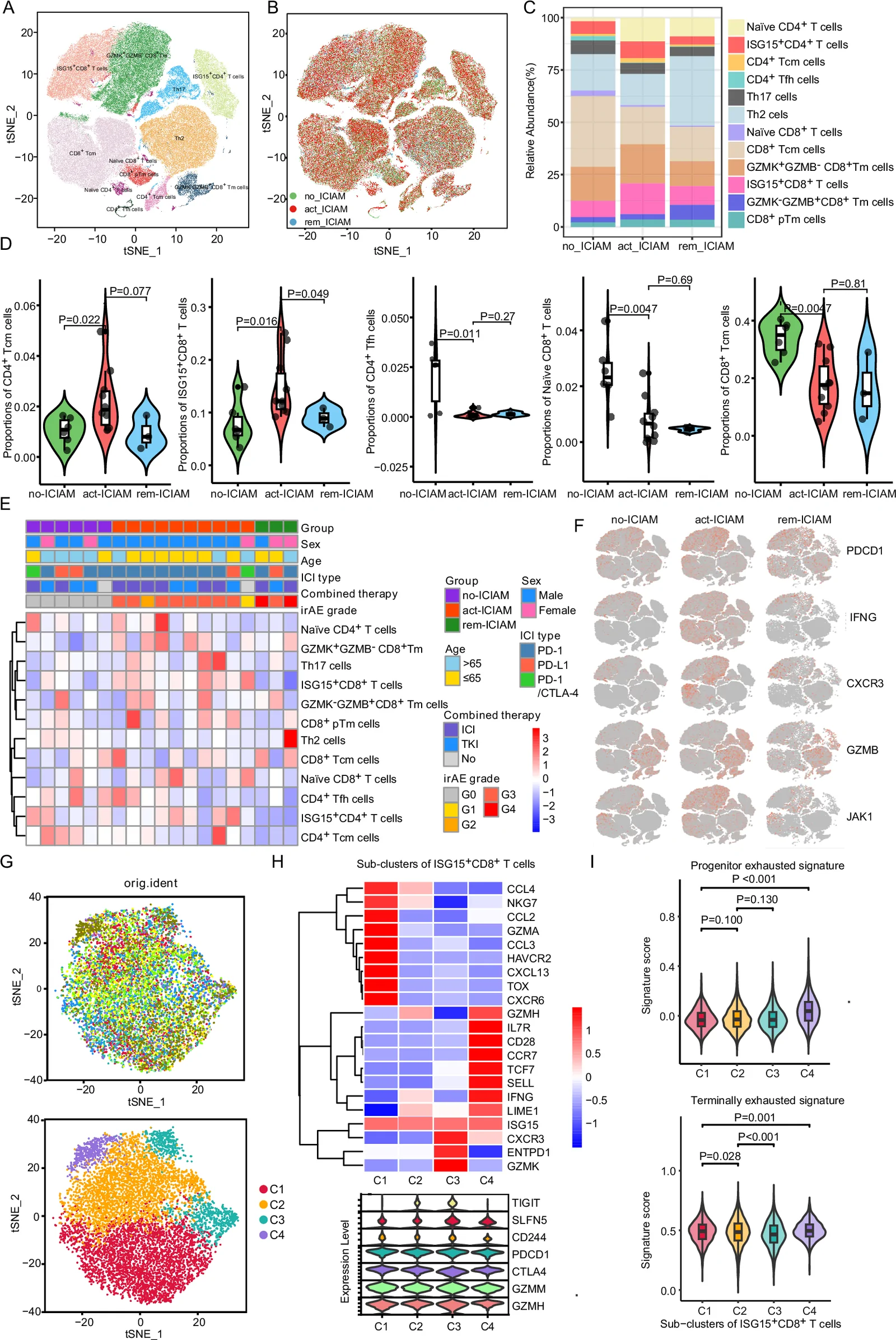
Identification of ICI-myocarditis-associated ISG15^+^CD8^+^ T cells. **A** Clustering of 144,116 T cells based on all the enrolled samples (*n* = 19), colored and labeled by T cell clusters. **B** t-SNE plot showing T cells colored by the no-ICIAM group, act-ICIAM group, and rem-ICIAM group. **C** Bar plot depicting the relative proportions of each T cell subcluster across the three groups. **D** Boxplots showing the proportions of CD4^+^ Tcm cells, ISG15^+^CD8^+^ T cells, CD4^+^ Tfh cells, naïve CD8^+^ T cells, and CD8^+^ Tcm cells from no-ICIAM (*n* = 6), act-ICIAM (*n* = 10) and rem-ICIAM (*n* = 3) groups. *P* values were calculated using the two-tailed Mann-Whitney U test. **E** Heatmap representing the clinical characteristics associated with major T cell subpopulations. **F** t-SNE plots showing the expression of genes of interest across T cell subclusters in each group. **G** Subclustering of ISG15^+^CD8^+^ T cells from the act-ICIAM group into four subsets. **H** Heatmap showing the expression of marker genes across subsets of ISG15^+^CD8^+^ T cells (top) and violin plots showing the expression of the indicated genes across subsets of ISG15^+^CD8^+^ T cells (bottom). **I** Quantification of the differences in the progenitor exhausted and terminally exhausted signature scores among four subsets of ISG15^+^CD8^+^ T cells in the act-ICIAM (*n* = 10) group. *P* values were calculated by a two-sided unpaired Student’s *t*test. ICI, immune checkpoint inhibitor; no-ICIAM, no ICI-myocarditis; act-ICIAM, active ICI-myocarditis; rem-ICIAM, remitted ICI-myocarditis; t-SNE, t-distributed stochastic neighbor embedding. Box plots depicted the interquartile range (box), median (center line), and full data range (whiskers). Mean ± SD were shown where appropriate.

Given that ICI targets are predominantly expressed on T cells, we performed a comprehensive analysis of 144,116 T cells from samples of no-ICIAM, act-ICIAM, and rem-ICIAM groups. We identified and annotated 12 main T cell subsets according to the expression of canonical gene markers^12^ (Supplementary Table 4). Among them, 6 were CD4^+^ T cell subclusters, 6 were CD8^+^ T cell subclusters (Fig. 2A and Supplementary Fig. 2A). Analysis of these clusters unveiled multiple T cell subtypes and transcriptional states, including naïve (Tn), ISG15^+^CD8^+^ T cells, and pTm (proliferating Tm) CD8^+^ T cells, in line with a recent study^7^. The distribution of these T cell clusters across different groups was visualized using t-SNE (Fig. 2B). Compared with the no-ICIAM group, the act-ICIAM group displayed significantly higher fractions of CD4^+^ Tcm cells and ISG15^+^CD8^+^ T cells but significantly lower fractions of CD4^+^ Tfh cells, naïve CD8^+^ T cells and CD8^+^ Tcm cells (Fig. 2C–E). To dissect the differences in T cell phenotypes, we analyzed the expression of panels of genes associated with four key categories^14^: exhaustion marker (PDCD1), inflammatory marker (IFNG), trafficking marker (CXCR3) and cytotoxicity marker (GZMB), in subclusters of interest (Fig. 2F). The expression of these markers was significantly higher in T cells from act-ICIAM group compared with the no-ICIAM or rem-ICIAM groups, suggesting a more activated T cell state in active disease. An exception was that JAK1, which was also a marker of ICI resistance, was consistently expressed across almost all T cell subpopulations in the act-ICIAM group (Fig. 2F).

We therefore focused on the ISG15^+^CD8^+^T cells, which were most prominently elevated in act-ICIAM patients but markedly decreased in no-ICIAM or rem-ICIAM groups. We extracted the ISG15^+^ CD8^+^ T cells in the act-ICIAM group and further subgrouped them into 4 clusters (C1-C4) to explore potential heterogeneity (Fig. 2G and Supplementary Fig. 2B). Notably, ISG15^+^CD8^+^ T cells-C1 was marked by elevated expression of terminal exhaustion markers (PDCD1, ENTPD1, HAVCR2, and TOX), whereas ISG15^+^CD8^+^ T cells-C4 was marked by elevated expression of progenitor exhausted cell markers (PDCD1, TCF7, IL7R, and SELL) (Fig. 2H). In irAEs, such as ICI-induced colitis and arthritis, CD8^+^ T cells include a subset of terminally exhausted cells, characterized by high expression of PD-1 and TIM-3, impaired effector function, and pro-inflammatory cytokine secretion, driving chronic inflammation and irAE progression^15,16^. To determine whether ISG15^+^CD8^+^ T cells exhibit a progenitor-exhausted phenotype, we scored each cell using well-established terminal- and progenitor-exhausted gene signatures^17^. The results revealed that ISG15^+^CD8^+^ T cells-C4 exhibited significantly higher activity in the progenitor-exhausted signature (PDCD1, TCF7, IL7R, SELL), whereas ISG15^+^CD8^+^ T cells-C1 were significantly enriched in the terminally-exhausted signature (PDCD1, ENTPD1, HAVCR2, TOX) (Fig. 2I).

**Fig. 3:**
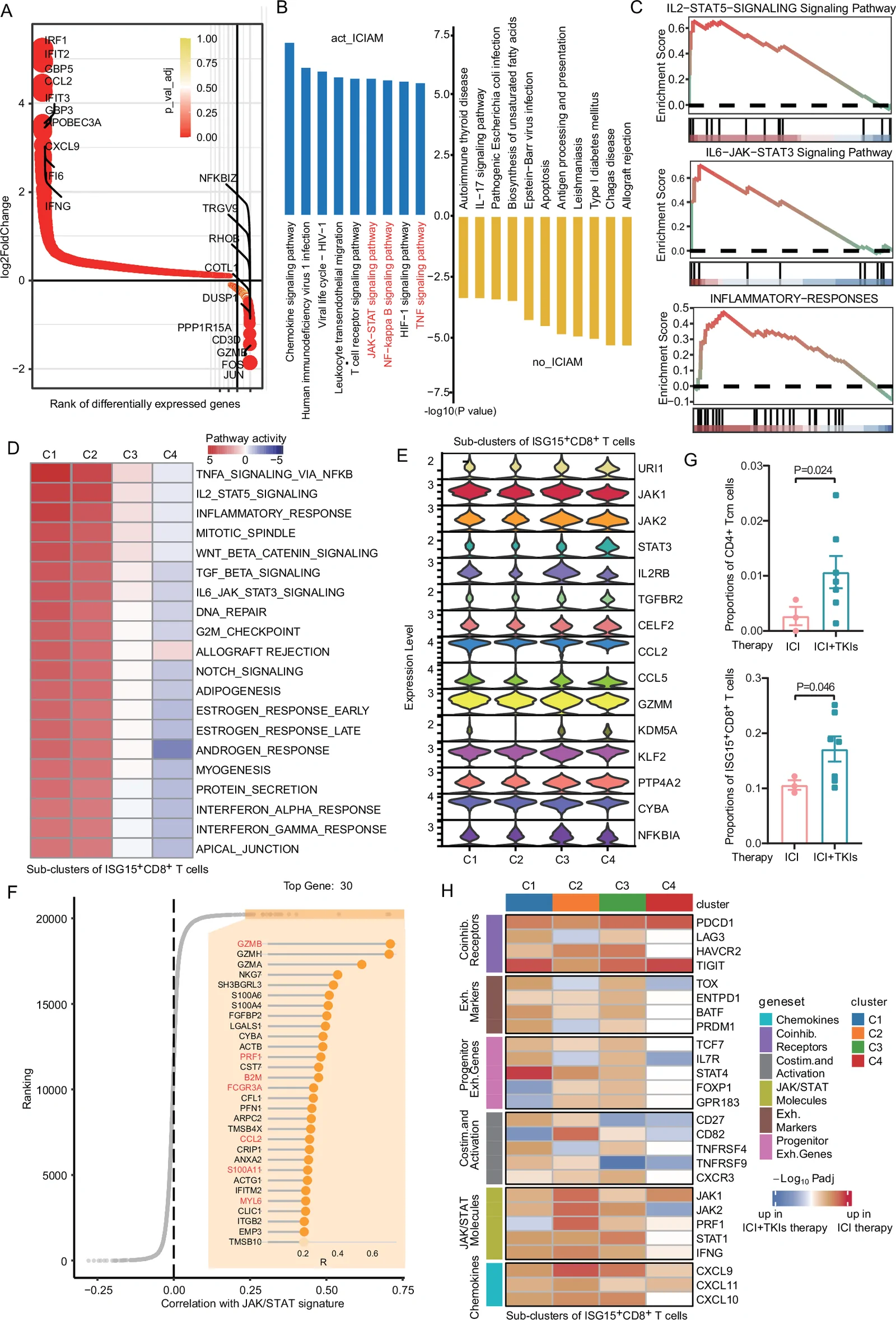
Activated JAK-STAT signaling was characteristic of ISG15^+^CD8^+^ T cells and further amplified following ICI + TKIs combination therapy. **A** Differentially expressed genes between act-ICIAM and no-ICIAM-derived ISG15^+^ CD8^+^ T cells, with the top ten and bottom ten differentially expressed genes labeled. The adjusted p-values were calculated using a two-sided Wilcoxon rank-sum test. **B** The enriched pathway activities for T cells between no-ICIAM (*n* = 6), act-ICIAM (*n* = 10) samples. A hypergeometric test was used. A Benjamini-Hochberg-adjusted *P* < 0.05 (two-sided) was used as a significance cutoff. **C** Gene set enrichment analysis (GSEA) plots revealing significant enrichment of the JAK-STAT signaling, chemokine signaling, and cytokine-cytokine receptor interaction pathways in act-ICIAM samples compared with no-ICIAM samples. **D** Differences in pathway activities scored per cell using GSVA, between ISG15^+^ CD8^+^ T cells isolated from no-ICIAM or act-ICIAM patients. **E** Violin plots showing the smoothed expression distribution of selected genes involved in T cell activation, immune checkpoints, and inflammation, stratified by ISG15^+^ CD8^+^ T cell subcluster. **F** Spearman correlation between the JAK-STAT signaling activity (the average expression of JAK-STAT signature genes) and the expression of CD8^+^ T cell-specific genes. Genes encoding known therapeutic targets in diseases with dysregulated JAK-STAT signaling, such as rheumatoid arthritis, are highlighted in red. **G** Subgroup analysis comparing ICI+TKIs combination therapy versus ICI monotherapy in terms of CD4^+^ Tcm cells and ISG15^+^ CD8^+^ T cells (*n* = 16). Two-sided unpaired Student’s *t*test was performed. **H**, Heatmap showing the relative changes in the signature gene expression within each ISG15^+^CD8^+^ T cell cluster between act-ICIAM patients treated with ICI+TKIs and those receiving ICI monotherapy. A two-sided Wilcoxon rank-sum test with Benjamini-Hochberg correction was performed. ICI, immune checkpoint inhibitor; no-ICIAM, no ICI-myocarditis; act-ICIAM, active ICI-myocarditis; TKIs, tyrosine kinase inhibitors. Box plots depicted the interquartile range (box), median (center line), and full data range (whiskers). Mean ± SD were shown where appropriate.

To investigate the pathophysiological mechanisms underlying ICI-myocarditis, we compared gene expression and pathway activities in the ISG15⁺CD8⁺ T cells between no-ICIAM and act-ICIAM patients. Compared to no-ICIAM patients, we found the top 10 differentially expressed genes (DEGs) in patients with act-ICIAM included chemokines (CXCL9, CCL2), interferon-inducible genes (IFIT2, IRF1, GBP1, GBP5, IFNG) and a gene associated with a pro-inflammatory phenotype (APOBEC3A) (Fig. 3A). Further analysis revealed pro-inflammatory changes, including activation of TNF signaling pathway, NF-κB signaling pathway, and JAK-STAT signaling pathway, which were largely consistent across different ISG15^+^CD8^+^ T cell subtypes (Fig. 3B). Building on these findings, GSEA identified enrichment of key inflammatory pathways, such as JAK-STAT signaling, chemokine signaling, and cytokine-cytokine receptor interaction. (Fig. 3C), highlighting their central role in immune dysregulation. However, some pathways were differentially regulated across CD8^+^ T cell subtypes. In the ISG15^+^CD8^+^ T cells, we identified highly inflammatory subclusters (TNFA signaling via NF-κB, IL6-JAK-STAT3 signaling, inflammatory response: clusters 1–3) (Fig. 3D). These clusters exhibited robust JAK-STAT activation with upregulation of JAK1, JAK2, STAT3, TGFBR2, CCL2, CCL5, NFKBIA, and CYBA, indicative of T-cell exhaustion and heightened inflammation (Fig. 3E). Furthermore, we identified several other molecules that exhibited a similarly strong correlation with the JAK-STAT signaling signature, suggesting their potential as candidate therapeutic targets for ICI-myocarditis (Fig. 3F).

Because combination therapy, such as tyrosine kinase inhibitors (TKIs), increases the risk of irAE compared to ICI monotherapy^18^, we investigated transcriptional differences in CD8^+^ T cells from ICI-myocarditis patients treated with ICI+TKIs versus ICI monotherapy. We found that CD4^+^ Tcm cells and ISG15^+^ CD8^+^ T cells were markedly enriched in patients receiving ICI+TKIs combination therapy (Fig. 3G). Moreover, ISG15^+^CD8^+^ T cells from patients receiving ICI+TKIs therapy showed upregulation of genes associated with enhanced TCR signaling (CD27, TNFRSF4), tissue homing and inflammation (CXCL9, CXCL10, CXCL11), coinhibitory receptors (PDCD1, LAG3, HAVCR2, TIGIT), exhaustion markers (ENTPD1, BATF), and inflammatory activities mediated by JAK-STAT signaling (JAK1, JAK2, PRF1, STAT1, IFNG) (Fig. 3H). In summary, ICI-myocarditis was characterized by an expansion of ISG15^+^CD8^+^ T cells, which exhibited a pro-inflammatory/exhausted state, and were more abundant in ICI+TKIs therapy than in those receiving ICI monotherapy, thus potentially accounting for the increased ICI-myocarditis risk.

### Determinants of act-ICIAM from peripheral immune cell populations

**Fig. 4:**
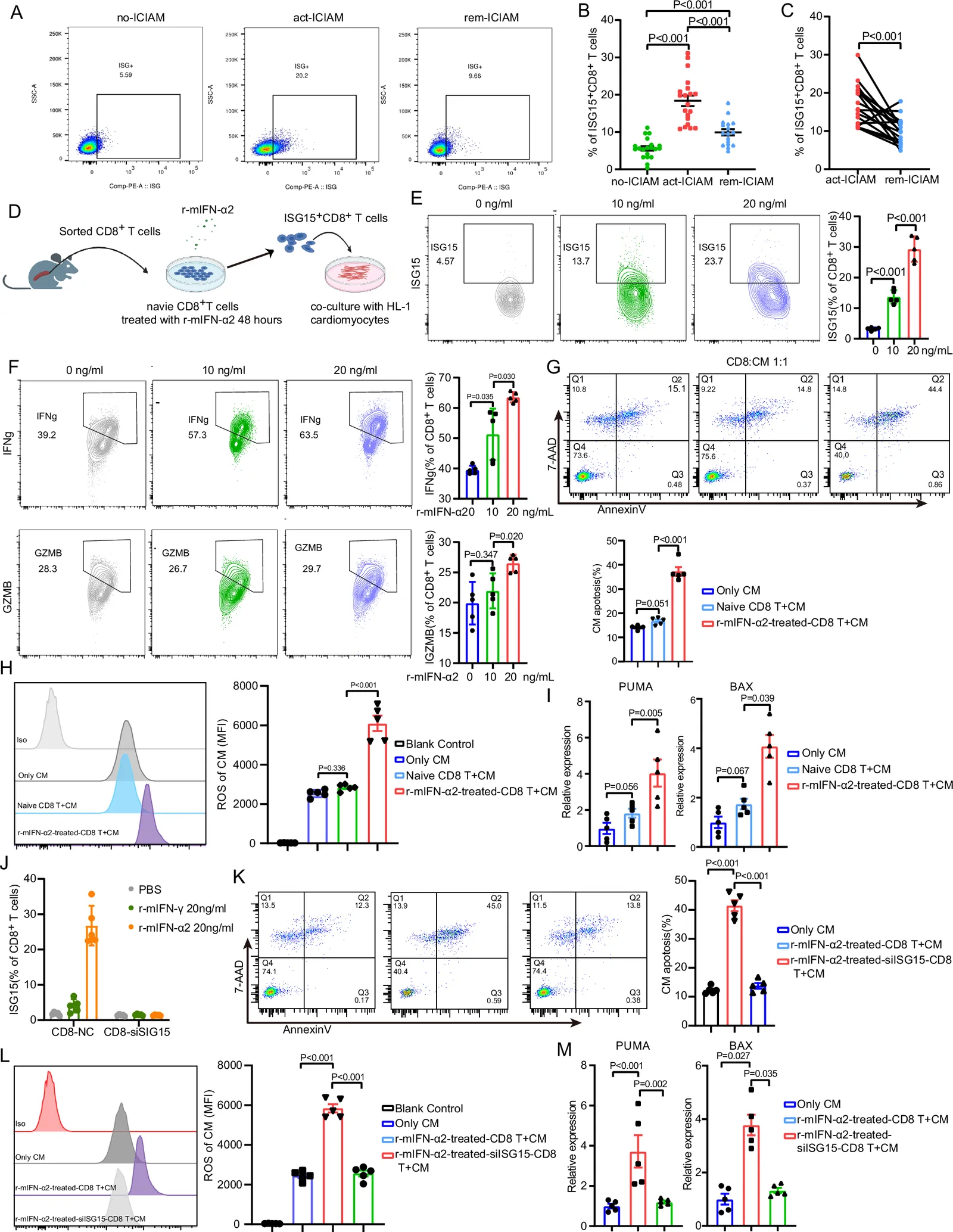
ISG15⁺ CD8⁺ T cells induce cardiomyocyte apoptosis in co-culture assays. **A**,**B** Representative flow cytometry plots (**A**) and quantification (**B**) showing the percentage of ISG15⁺CD8⁺ T cells in the indicated groups. Two-sided unpaired Student’s *t*test was performed. **C** Paired comparison of ISG15⁺CD8⁺ T cells between act-ICIAM and rem-ICIAM samples. A paired two-tailed Student’s *t*test was performed. **D** Schematic workflow of the co-culture assay between cardiomyocytes and ISG15⁺CD8⁺ T cells. **E** Flow cytometry analysis of ISG15 expression in CD8⁺ T cells following stimulation with recombinant mouse IFN-α2 (r-mIFN-α2) at the indicated concentrations (0, 10 and 20 ng/mL) (*n* = 5 biologically independent experiments). Two-sided unpaired Student’s *t*test was performed. **F** Flow cytometry analysis of IFNG and GZMB expression in CD8⁺ T cells following stimulation with r-mIFN-α2 at the indicated concentrations (0, 10 and 20 ng/mL) (*n* = 5 biologically independent experiments). A two-sided unpaired Student’s *t*test was performed. **G** Quantification of cardiomyocyte apoptosis after co-culture with ISG15⁺CD8⁺ T cells at a ratio of 1:1 (ISG15⁺CD8⁺ T:CM) (*n* = 5 biologically independent experiments). Two-sided unpaired Student’s *t*test was performed. **H** Quantitative analysis of reactive oxygen species (ROS) levels in cardiomyocytes cocultured with ISG15⁺CD8⁺ T cells at a ratio of 1:1 (*n* = 5 biologically independent experiments). A two-sided unpaired Student’s *t*test was performed. I, Relative expression levels of BAX and PUMA in cardiomyocytes following co-culture with ISG15⁺CD8⁺ T cells at a ratio of 1:1 (*n* = 5 biologically independent experiments). A two-sided unpaired Student’s *t*test was performed. J, The specific siRNA was designed to knock down ISG15 expression in CD8^+^ T cell and the knockdown efficiency was measured by flow cytometry (*n* = 5 biologically independent experiments). A two-sided unpaired Student’s *t*test was performed. **K**, Quantification of cardiomyocyte apoptosis after co-culture with CD8⁺ T cells following ISG15 siRNA-mediated knockdown (*n* = 5 biologically independent experiments). A two-sided unpaired Student’s *t*test was performed. **L**, Measurement and quantitative analysis of ROS levels in cardiomyocytes co-cultured with CD8^+^ T cells after ISG15 siRNA transfection (*n* = 5 biologically independent experiments). A two-sided unpaired Student’s *t*test was performed. **M**, Relative expression of BAX and PUMA in cardiomyocytes co-cultured with CD8^+^ T cells after ISG15 siRNA transfection (*n* = 5 biologically independent experiments). A two-sided unpaired Student’s *t*test was performed. ICI, immune checkpoint inhibitor; no-ICIAM, no ICI-myocarditis; act-ICIAM, active ICI-myocarditis; CM, cardiomyocytes; PBS, phosphate-buffered saline. Box plots depicted the interquartile range (box), median (center line), and full data range (whiskers). Mean ± SD were shown where appropriate.

Next, to systematically explore the association between different immune cell types and ICI-myocarditis, we employed two indices: the predictive index (Pi) and the therapeutic index (Ti)^19^. For the Pi analysis, CD4^+^ Tfh cells and ISG15^+^CD8^+^ T cells emerged as the most prominent predictors of high ICI-myocarditis risk (Supplementary Fig. 3A). Based on the Ti analysis, CD8^+^ Tcm cells had a significant negative Ti, suggesting a potential role for these cells as effectors in immune regulation. In contrast, ISG15^+^CD8^+^ T cells showed positive Ti, suggesting that drugs might trigger ICI-myocarditis responses by increasing ISG15^+^CD8^+^ T cells with exhaustion potential (Supplementary Fig. 3B). Notably, patients with high-grade of ICI-myocarditis had increased frequencies of ISG15^+^CD8^+^ T cells (Supplementary Fig. 3C, D). Next, we used flow cytometry to validate this finding in the flow cytometry cohort (Supplementary Table 2). The frequencies of ISG15^+^CD8^+^ T cells were significantly higher in act-ICIAM patients relative to both no-ICIAM and rem-ICIAM patients (Fig. 4A, B and Supplementary Fig. 3E). In addition, the tumor type did not seem to affect the observed expansion of ISG15^+^CD8^+^ T cells (Supplementary Fig. 3F). However, due to small sample sizes in the non-gastrointestinal cancer subgroup, the difference failed to reach statistical significance (Supplementary Fig. 3G). Interestingly, several patients in this cohort with additional non-ICIAM irAEs also exhibited elevated levels of ISG15^+^CD8^+^ T cells (Supplementary Fig. 3H). In contrast, higher frequencies of peripheral ISG15^+^CD8^+^ T cells in ICI-myocarditis patients were not associated with improved tumor responses (Supplementary Fig. 3I).

An important question regarding the increased ISG15^+^CD8^+^ T cells in the act-ICIAM group was whether they represented transient or stable populations. Thus, we used monocle pseudotime trajectory analysis to dissect the trajectories of CD8^+^ T cells. These findings suggest a continuous differentiation trajectory of CD8^+^ T cells, starting from naïve cells and transitioning through central memory (Tcm) status into distinct effector memory subsets, including GZMK^+^GZMB^-^CD8^+^ Tm cells, ISG15^+^CD8^+^ T cells and GZMK^-^GZMB^+^CD8^+^ Tm cells, ultimately leading to highly cytotoxic populations (Supplementary Fig. 4A), representing critical differentiation stages as CD8^+^ T cells transition from a less cytotoxic to a more cytotoxic phenotype under persistent immunological pressure^16,20,21^. Interestingly, as cells progressed, we observed a progressive increase in the expression of immune checkpoint molecules (LAG3), IFN-γ response mediators (IFNG, STAT1), tissue-homing signals (CCL5), and key cytotoxic effectors (GZMB, PRF1), reflecting a gradual shift toward a more activated and potentially pathogenic T cell phenotype (Supplementary Fig. 4B, C). To directly assess the temporal dynamics of ISG15⁺CD8⁺ T cells, we performed a longitudinal analysis of peripheral blood samples from the same patients during active myocarditis and after clinical remission. Results showed that ISG15⁺CD8⁺ T cells expanded in the act-ICIAM and declined in the rem-ICIAM, supporting a transient expansion of this population in association with active disease (Fig. 4C).

To independently assess the cardiac microenvironment in ICI-myocarditis at single-cell resolution, we analyzed publicly available scRNA-seq data (GSE228597)^11^. A total of 19,032 high-quality cells passed data quality control, and 10 major cell lineages were identified in the cardiac tissues, including mural cells, cardiomyocytes, endothelial cells, fibroblasts, NK cells, Mon/Mac, DCs, CD8^+^ T cells, CD4^+^ T cells, and B/plasma cells (Supplementary Fig. 5A, B). We next analyzed the CD8^+^ T cells compartments in detail. As a result, six CD8^+^ T cell subclusters were identified, including CXCR6^+^CD8^+^Trm cells, CD8^+^ cytotoxic T cells (CTLs), ISG15^+^CD8^+^ T cells, CD8^+^ Tex cells, CD8^+^ Tem cells, and cycling CD8^+^ T cells (Supplementary Fig. 5C–E). Similar to the results for PBMCs, ISG15^+^CD8^+^ T cells in the cardiac tissues also demonstrated a pronounced pro-inflammatory feature, including IL6-JAK-STAT3, IFN-γ response, IFN-α response, IL2-STAT5 and TNF signaling through NF-κB pathways (Supplementary Fig. 5F).

### ISG15⁺ CD8⁺ T cells had potential cytotoxic effects on cardiomyocytes

In the context of ICI-myocarditis, pathogenic T cells may induce apoptosis of cardiomyocytes^8,22^. To directly validate the cytotoxicity of ISG15⁺ CD8⁺ T cells on cardiomyocytes, we conducted a T cell-mediated cell-killing assay by co-culturing ISG15^+^CD8^+^ T cells and HL-1 cardiomyocytes (Fig. 4D). Previous studies have established that ISG15 is a canonical interferon-stimulated gene that is robustly upregulated downstream of type I interferon signaling and can be induced by IFN-α2^23–25^. Thus, we isolated mouse splenic CD8⁺ T cells and cocultured ex vivo and stimulated with recombinant mouse IFN-α2 (r-mIFN-α2) to induce ISG15 expression^26–28^. Notably, dose-response experiments using r-mIFN-α2 stimulation (0, 10, and 20 ng/mL) efficiently induced the differentiation of CD8⁺ T cells into an ISG15⁺ CD8⁺ T cell population (Fig. 4E). In agreement with single-cell transcriptomic findings, ISG15⁺ CD8⁺ T cells showed elevated expression of GZMB and IFNG (Fig. 4F). Among the tested conditions, stimulation with 20 ng/mL r-mIFN-α2 resulted in the most robust and stable ISG15 induction and was therefore used for subsequent functional assays (Fig. 4E). In contrast, stimulation with type II interferon (r-mIFN-γ) failed to elicit comparable effects (Supplementary Fig. 6A).

In the T cell-mediated cell-killing assay, ISG15⁺CD8⁺ T cells cocultured with cardiomyocytes at a 1:1 ratio (CD8:CM) exhibited significantly increased apoptosis (Fig. 4G), elevated reactive oxygen species (ROS) production (Fig. 4H), and enhanced expression of apoptosis markers BAX and PUMA compared with controls (Fig. 4I). Notably, these cytotoxic effects remained evident even at a lower ratio of 1:10 (Supplementary Fig. 6B, C), indicating a robust cardiomyocyte-damaging capacity of ISG15⁺CD8⁺ T cells. To directly investigate the role of ISG15, we knocked down ISG15 in CD8⁺ T cells using siRNA. We confirmed that the expression of ISG15 was significantly lower than that in the NC-siRNA groups (Fig. 4J). Notably, ISG15-knockdown CD8⁺ T cells failed to induce cardiomyocyte apoptosis or ROS production and were associated with markedly reduced expression of BAX and PUMA in cardiomyocytes (Fig. 4K–M). These findings provide functional evidence supporting a capacity of ISG15⁺ CD8⁺ T cells to induce cardiomyocyte injury in vitro.

### ISG15^+^CD8^+^ T cells drove cell communication primarily via the CCL2-CCR2 axis

**Fig. 5:**
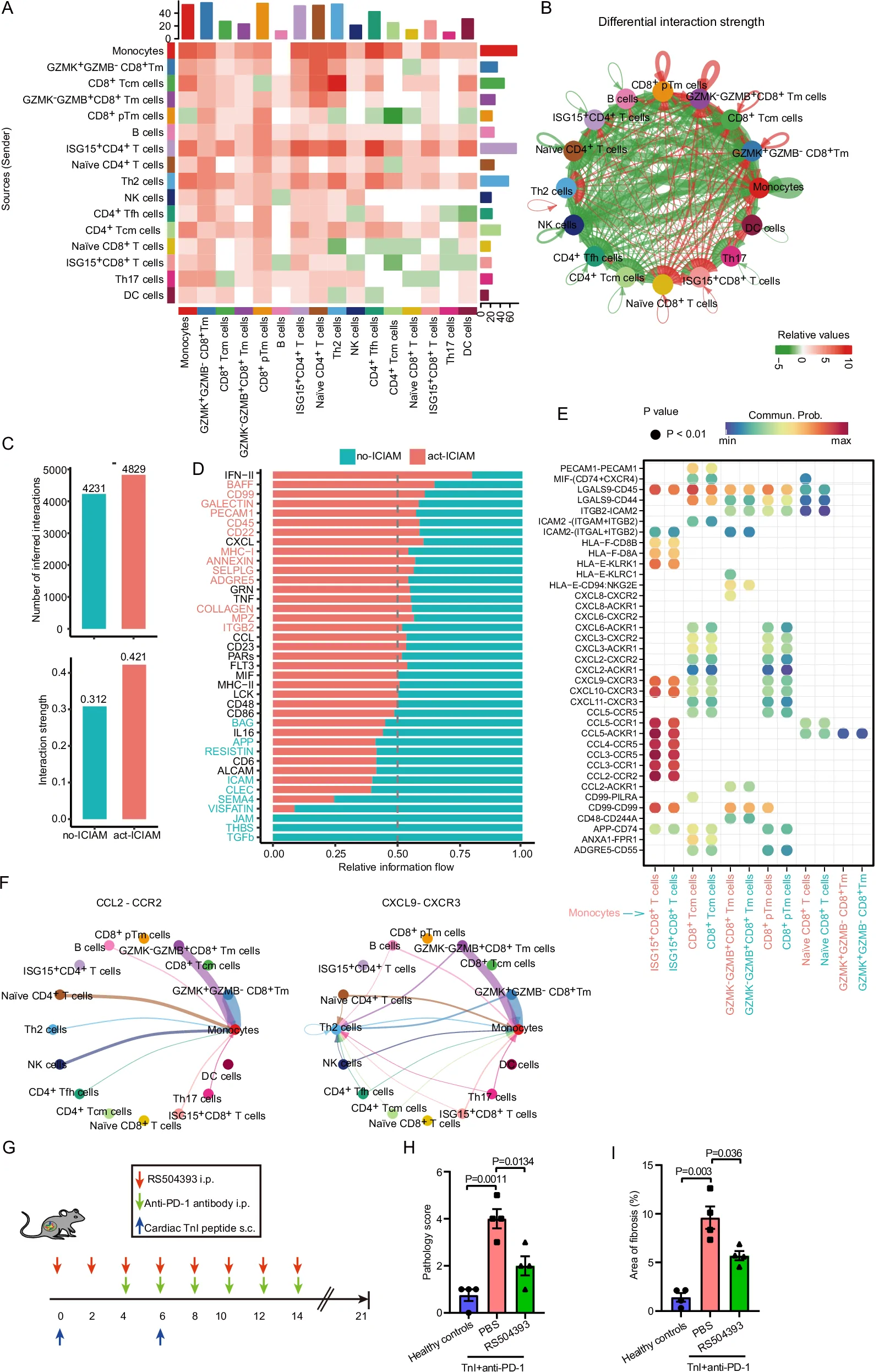
Interaction between T cells and monocytes drives a proinflammatory environment. **A**,**B** Heatmaps (**A**) and circle plots (**B**) showing the interaction quantity and interaction strength across immune cell subtypes between the no-ICIAM group and act-ICIAM group. Red indicates the increase of communication in the latter. All interactions were statistically significant by permutation tests. **C** Number of inferred interactions and interaction scores between the no-ICIAM and act-ICIAM groups. **D** Bar graph showing differences in the overall information flow of significant signaling pathways between the no-ICIAM group and the act-ICIAM group. Pathways enriched in the no-ICIAM group are shown in red (top), whereas those enriched in act-ICIAM are shown in blue (bottom). **E** Bubble plot showing the differentially expressed ligand-receptor pairs between the no-ICIAM group and the act-ICIAM group. The color (from the blue to red) and size of the dots represent the communication probability and *P* values (shown on the right), respectively. The empty spaces indicate that the communication probability was zero. *P* values are computed using two-sided permutation tests. **F** Circle plots highlighting significant CCL2-CCR2 and CXCL9-CXCR3 ligand-receptor pairs across immune cell subtypes. **G** Schematic representation of the RS504393 treatment protocol in a C57BL/6 mouse model of ICI-myocarditis induced by cardiac troponin I (cTnI) peptide immunization and anti-PD-1 administration (*n* = 4). **H**,**I** The pathology scores (**H**) and fibrosis area (**I**) in the mouse hearts from each group (*n* = 4). Two-sided unpaired Student’s *t*test was performed. ICI, immune checkpoint inhibitor; no-ICIAM, no ICI-myocarditis; act-ICIAM, active ICI-myocarditis; TnI troponin I; anti-PD-1, anti-programmed cell death protein 1 antibody; PBS, phosphate-buffered saline. Box plots depicted the interquartile range (box), median (center line), and full data range (whiskers). Mean ± SD were shown where appropriate.

To investigate immune cell crosstalk shaping ICI-myocarditis pathogenesis, we performed an unbiased ligand-receptor interaction analysis using the CellChat framework. This analysis revealed numerous ligand-receptor pairs and signaling networks (Fig. 5A, B and Supplementary Fig. 7A, B). Interaction intensity was markedly higher in the act-ICIAM group compared to the no-ICIAM group (Fig. 5C), with CD8^+^ T cells and CD4^+^ T cells forming close interaction links with monocytes (Fig. 5A, B and Supplementary Fig. 7A, B). Notably, ISG15^+^CD8^+^ T cells and monocytes exhibited the most prominent cellular communication. Compared to no-ICIAM, CD8^+^ T cells showed increased incoming communication intensity, while monocytes displayed enhanced outgoing communication in the act-ICIAM group (Supplementary Fig. 7C). The act-ICIAM samples exhibited significantly enriched interactions involving CCL, CXCL, TNF, CD99, IL16, and BAFF signaling pathways compared to no-ICIAM controls (Fig. 5C, D), highlighting their regulatory roles in the peripheral microenvironment of ICI-myocarditis. Specifically, monocytes closely interacted with CD8^+^ T cells through ligand-receptor pairs such as CCL2-CCR2, CXCL9-CXCR3, LGALS9-CD44, and HLA-DMB-CD4 (Fig. 5E, F and Supplementary Fig. 7D, E). Interestingly, previous studies identified that Cxcl9^+^Cxcl10^+^ macrophages, derived from CCR2^+^ monocytes, expand in Ctla4^+^/-Pdcd1-/- mouse hearts and contribute to ICI-myocarditis pathogenesis^9^. Consistent with these findings, myocarditis-associated CD8^+^ T cells highly expressed chemokine receptor genes CXCR3 and CCR2, while monocytes expressed their ligands CXCL9, CXCL10, and CCL2 (Supplementary Fig. 7F, G). In addition to the reported CXCL9/CXCL10-CXCR3 axis in ICI-myocarditis^9^, our findings showed a potential mechanism wherein monocytes contributed to ICI-myocarditis pathogenesis by recruiting ISG15^+^CD8^+^ T cells through the CCL2-CCR2 axis. In the ICI-myocarditis model, we administered RS504393, a specific CCR2 antagonist, to inhibit CCL2-CCR2 axis in vivo (Fig. 5G). After three weeks, RS504393 significantly alleviated both inflammatory infiltration and interstitial fibrosis in cardiac tissues (Fig. 5H, I). Therefore, blocking the CCL2-CCR2 axis could markedly alleviate myocardial damage in myocarditis, highlighting the therapeutic potential of targeting this axis in clinical intervention.

### Monocytes exhibited elevated metabolic profiles and NLRP3 inflammasome pathway activity in act-ICIAM patients

Next, given the marked reduction in circulating monocyte frequency during active disease, we performed a detailed analysis of monocyte subsets. We identified six transcriptionally distinct monocyte subpopulations, including S100A12^hi^ CD14⁺monocytes, IL1B^hi^ CD14⁺ monocytes, CD16⁺ monocytes, IFN-activated (IFN^act^) CD14⁺ monocytes, HLA-DRB5⁺CD14⁺ monocytes, and IL1B^hi^ CD16^+^ monocytes (Supplementary Fig. 8A, B). Notably, S100A12^hi^CD14⁺monocytes and IL1B^hi^ CD14⁺ monocytes were increased in act-ICIAM group (Supplementary Fig. 8C), and DEG analysis indicated that S100A12^hi^ CD14⁺ monocytes were the most influenced subset between no-ICIAM and act-ICIAM (Supplementary Fig. 8D, E). When analyzing gene expression related to myeloid cell functions, most monocyte subclusters in the act-ICIAM patients exhibited significantly higher levels of inflammation- and antigen presentation-related pathways (Supplementary Fig. 8F). In addition, phagocytosis-related signatures were consistently higher in all subclusters in act-ICIAM patients (Supplementary Fig. 8F). To understand the different mechanisms of monocyte subsets, we then defined pathway-level differences in monocyte subsets between act-ICIAM and no-ICIAM groups. This revealed pervasive alterations, largely coherent across monocyte subtypes, including increased hypoxia, glycolysis and oxidative phosphorylation signatures (Supplementary Fig. 8G). In parallel, act-ICIAM monocytes exhibited marked enrichment of TLR and NOD-like receptor signaling, NF-κB activation, which may collectively provide the priming signal for NLRP3 inflammasome activation^29,30^, and enhanced cytokine and chemokine signaling modules (Supplementary Fig. 8G).

### Olink proteomic profiling revealed circulating inflammatory mediators for ICI-myocarditis detection

**Fig. 6:**
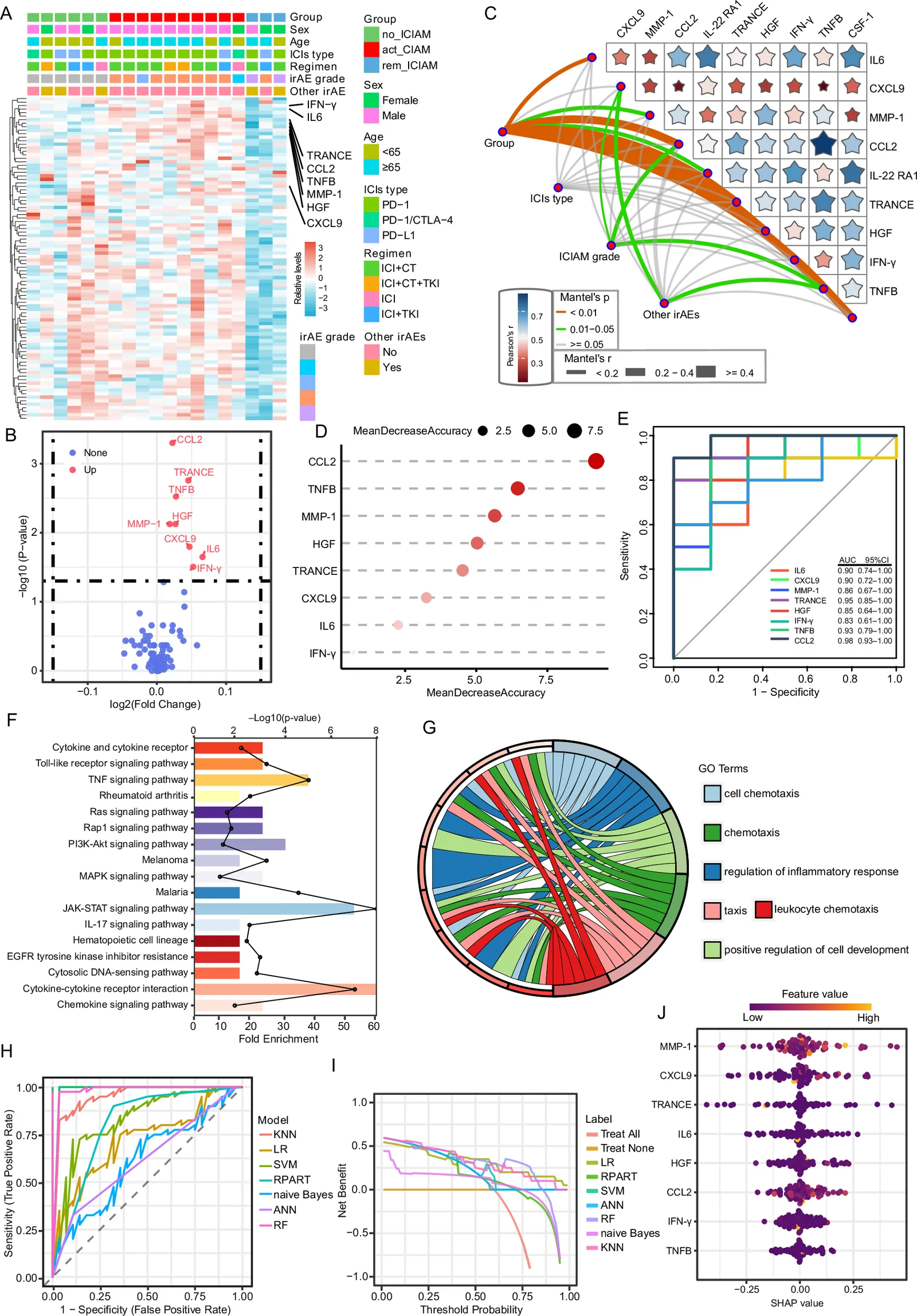
Integrative serum proteomic modeling identifies candidate biomarkers for ICI-myocarditis. **A** Heatmap showing the expression of 92 serum proteins in the single-cell cohort (*n* = 19). **B** Volcano plot displaying differentially expressed proteins between the no-ICIAM and act-ICIAM groups. Significant proteins level with changes P < 0.05 were considered (*n* = 16). Two-tailed Wilcoxon rank sum test was used. **C** Mantel correlation network showing associations between clinical features and serum protein profiles. Edge width is proportional to Mantel r value, and the edge colors indicate statistical significance levels. Pairwise Mantel correlation coefficients of clinical variables are shown in color gradients. Mantel correlations were assessed using a two-sided permutation test (9999 permutations), and multiple comparisons were controlled using the Benjamini-Hochberg method. **D** Ranking of protein importance identified by the Random Forest analysis between the no-ICIAM and act-ICIAM groups (*n* = 16). **E** Receiver operating characteristic (ROC) curve analysis of eight candidate proteins, with the area under the curve (AUC) indicated. **F** KEGG pathway enrichment analysis of significantly altered proteins. Bars represent the number or proportion of proteins in each functional category, ranked by pathway coverage. Pathway enrichment was assessed using a hypergeometric test, with P values adjusted by Benjamini-Hochberg method. **G** GO term enrichment analysis of differentially expressed proteins. **H** ROC curves comparing the diagnostic performance of seven machine learning models, including random forest (RF), artificial neural network (ANN), naive Bayes, decision tree (RPART), support vector machine (SVM), logistic regression (LR), and k-nearest neighbors (KNN). **I** Decision curve analysis (DCA) assessing the clinical net benefit of each model. The x-axis represents the decision threshold, corresponding to the minimum predicted risk probability at which a clinical intervention would be considered. The y-axis indicates the net benefit, defined as the difference between true positives and the weighted number of false positives, offering insight into the clinical utility of each model. **J** SHAP (Shapley Additive exPlanations) beeswarm plot visualizing the contribution of individual features to the predictions made by the RF model. Vertical axis denotes feature importance ranked by mean SHAP value; the horizontal axis reflects whether each feature is associated with increased (right) or decreased (left) risk prediction. Color gradient indicates feature value (yellow, high; purple, low). ICI immune checkpoint inhibitor, no-ICIAM no ICI-myocarditis, act-ICIAM active ICI-myocarditis, irAE immune-related adverse event, CT chemotherapy, TT targeted therapy.

Having identified cellular immune profiles of ICI-myocarditis, we conducted an Olink multiplex inflammation panel^31^ in the in-house scRNA-seq cohort to assess soluble immune signatures of ICI-myocarditis. Broadly, most cytokine signatures were observed across different ICI regimens and various cancer types, although subgroup differences were not always statistically significant due to limited sample sizes (Fig. 6A). Notably, inflammation-related proteins were significantly enriched in the act-ICIAM group, whereas their levels were reduced in the no-ICIAM and rem-ICIAM groups (Fig. 6A), highlighting systemic hyperinflammation in ICI-myocarditis pathogenesis. Comparative analysis between the act-ICIAM and no-ICIAM groups revealed that 8 proteins, including immune cell-recruiting chemokines (CCL2 and CXCL9), inflammatory storm mediators (IL6, TNFB, TRANCE, and MMP-1), and tissue repair or remodeling factors (IFN-γ and HGF), were significantly upregulated in the act-ICIAM group (Fig. 6B). Correlation analysis between clinical traits and proteins identified strong associations, with specific proteins such as CCL2, MMP-1, TRANCE, TNFB, CXCL9, and HGF, demonstrating significant clinical relevance (Fig. 6C). Among these proteins, CCL2, TNFB, and MMP-1 emerged as the top three contributors to ICI-myocarditis severity, as determined by Random Forest analysis (Fig. 6D). ROC analysis showed that these proteins could discriminate act-ICIAM from ICI-treated controls without myocarditis (Fig. 6E). Furthermore, KEGG and GO enrichment analyses indicated enrichment of pathways related to cytokine, PI3K-Akt, and TNF signaling pathway, supporting the involvement of inflammatory processes in ICI-myocarditis (Fig. 6F, G).

To validate the robustness and translational potential of our findings, we developed a custom multiplex Luminex panel targeting 8 candidate proteins (Supplementary Fig. 9A). This platform enables high-throughput and accurate technical validation of our results^32^, and was applied to serum samples from the Luminex assay cohort (Supplementary Table 3). Consistent with trends observed in the Olink proteomic dataset, all eight candidate proteins were significantly elevated in patients with act-ICIAM compared to the no-ICIAM group (Supplementary Fig. 9B). Pearson correlation analysis suggested the independence of these candidate proteins (Supplementary Fig. 9C). Subsequently, the predictive performance of the 8-protein panel was evaluated using seven machine learning algorithms, including k-nearest neighbors (KNN), logistic regression (LR), support vector machine (SVM), recursive partitioning and regression trees (RPART), Naive Bayes (NB), artificial neural network (ANN), and random forest (RF). The dataset was internally validated using 10-fold cross-validation with 1,000 repetitions. Among these models, RF consistently delivered superior performance, achieving an AUC of 0.996 (95% CI: 0.99-1.00) (Fig. 6H). Thus, RF was selected as the final predictive model. In addition, confusion matrices from all models were presented in Supplementary Fig. 9D, confirming the robustness and stability of the RF classifier. These results suggest that the 8-protein custom panel holds promise as a set of predictive biomarkers for ICI-myocarditis. Decision curve analysis (DCA) demonstrated substantial net benefit in the panel among almost all of the threshold probabilities, underscoring the strong clinical utility of the RF model (Fig. 6I). To further interpret the RF model outputs, Shapley Additive Explanations (SHAP) analysis was used to quantify the contribution of each feature to ICI-myocarditis prediction. SHAP analysis indicated that MMP-1 had the greatest contribution to the model’s prediction, with an average SHAP value of 0.092. Other proteins, such as TRANCE and CXCL9, also exhibited high importance, contributing average SHAP values of 0.063 and 0.058, respectively (Fig. 6J and Supplementary Fig. 9E). These findings emphasized the critical role of systemic inflammation in ICI-myocarditis and provided candidate biomarkers and therapeutic targets for mitigating this event.

### Multi-omic analysis revealed shared inflammatory signatures between blood and myocardium

**Fig. 7:**
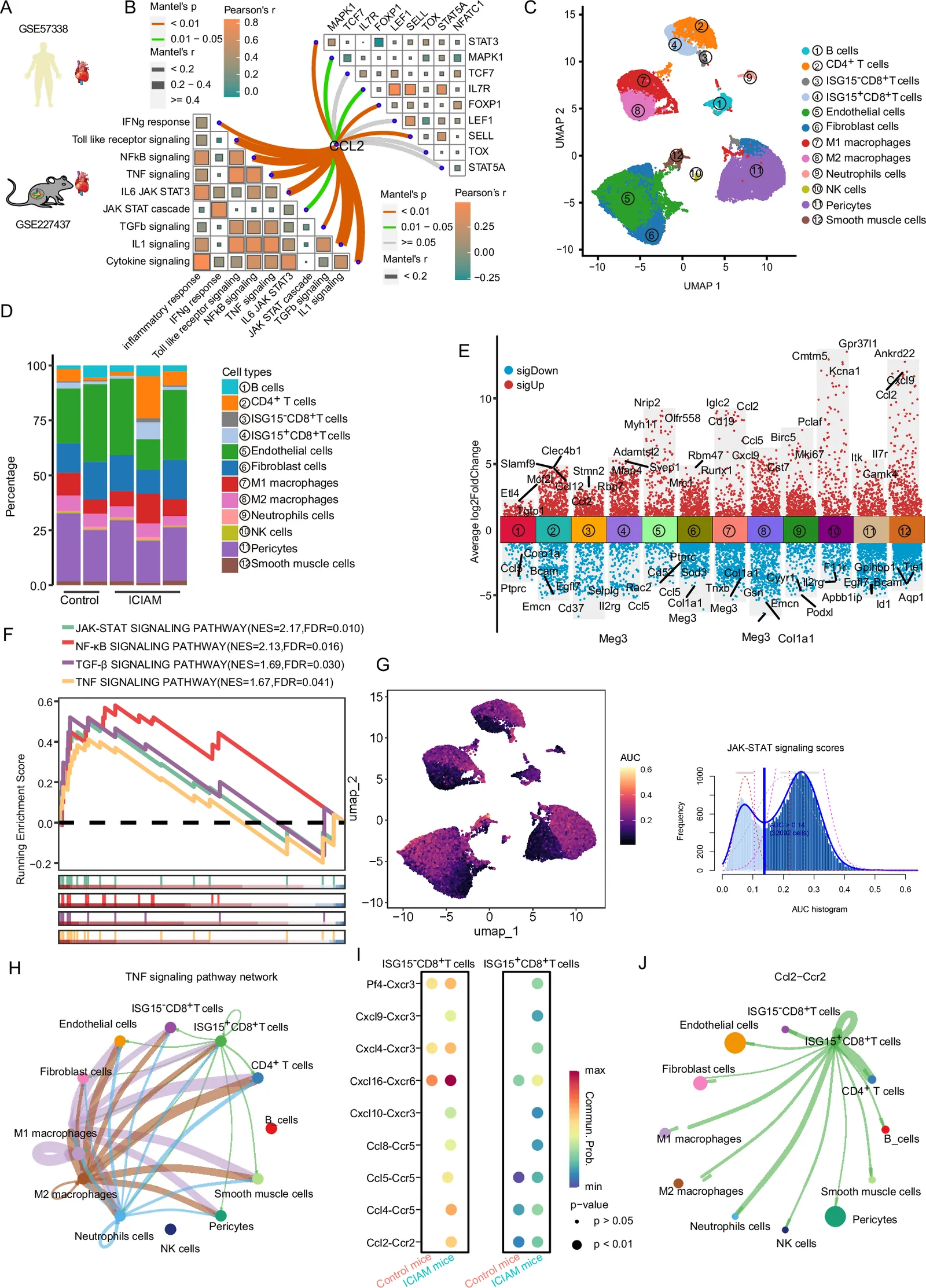
Multi-omic integration of peripheral blood and cardiac tissue identifies key inflammatory mediators in ICI-myocarditis. **A** Schematic illustration of cardiac sample sources. **B** Mantel’s correlations between CCL2, T cell exhaustion signatures, and inflammatory signals in the GSE57338 cohort (*n* = 313). Edge widths represent Mantel’s r values, and edge colors indicate statistical significance levels. Pairwise correlation coefficients are shown as color gradients. Mantel correlations were assessed using a two-sided permutation test (999 permutations), and multiple comparisons were controlled using the Benjamini-Hochberg method. **C** UMAP projection of 12 cell clusters derived from single-cell transcriptomes of cardiac tissues from Ctla4^+^/-Pdcd1-/- ICI-myocarditis mice in the GSE227437 (*n* = 5). **D** Cell cluster frequencies shown as fractions of total cells for each sample. **E** Volcano plot illustrating the top 5 upregulated and top 5 downregulated DEGs across cell clusters in cardiac tissues from ICI-myocarditis mice compared to controls. **F** GSEA of DEGs highlighting inflammatory pathways in cardiac tissues from ICI-myocarditis mice versus controls. **G** UMAP plots showing ISG scores (left) and JAK-STAT signaling scores (right) projected onto cardiac cells from Ctla4^+^/-Pdcd1-/- ICI-myocarditis mice. **H** CellChat analysis visualizing intercellular communication via TNF signaling among cardiac cell subsets. **I** Bubble plots depicting ligand-receptor interactions between macrophages and other cell types, with significant differences observed between ICI-myocarditis mice and controls. **J** Circular plots highlighting significant CCL2-CCR2 ligand-receptor interactions across cell subtypes in ICI-myocarditis mice compared to controls. ICI immune checkpoint inhibitor, ICIAM ICI-myocarditis, AUC area under the curve, UMAP uniform manifold approximation and projection.

To elucidate the impact of myocarditis-specific signatures on the cardiac microenvironment, we aimed to establish a connection between blood-derived molecular observations and events occurring within cardiac tissues. Given the limited availability of human cardiac tissues, we expanded our analysis to include the microarray expression data from human cardiac tissues (Fig. 7A) in the GEO dataset (GSE57338)^33^. Our analysis revealed a positive correlation between CCL2 expression and key regulators of T cell biology and inflammation, including STAT3, MAPK1, IL7R, and SELL. Consistently, we observed that CCL2 expression positively correlated with key inflammatory signaling pathways, such as IFN-γ response, Toll-like receptor signaling, NF-κB signaling, TNF signaling, and IL6-JAK-STAT3 signaling (Fig. 7B). Additionally, we validated the correlation between CXCL9 expression and inflammatory signatures (Supplementary Fig. 10A).

To corroborate these findings, we examined the scRNA-seq dataset (GSE227437)^9^, which profiles the immune and non-immune cardiac landscape in a Ctla4⁺/−Pdcd1 − /− mouse model of ICI-myocarditis (Fig. 7C, Supplementary Fig. 10B). The data revealed elevated proportions of CD68^+^ macrophages, CD4^+^ T cells, and CD8^+^ T cells in the ICI mouse model. Further analysis identified the expansion of ISG15^+^CD8^+^ T cells within the cardiac tissue (Fig. 7D). Additionally, non-immune cell populations, such as cardiac pericytes and fibroblasts, were detected. To uncover the underlying pathogenic mechanisms in affected heart tissues, we investigated the gene expression features of cell subsets between the control and ICI-myocarditis models. The top 5 DEGs between two groups across distinct cell subsets were chemokines, IFN-inducible genes and genes related to the M1 macrophage phenotype, T-cell exhaustion, and inflammatory signaling pathways (Fig. 7E).

GSEA showed that multiple inflammatory pathways were prominently enriched in the ICI-affected heart tissues, and were all designated as disease-associated inflammatory pathways, including JAK-STAT signaling, TNF signaling, and NF-κB signaling (Fig. 7F). To explore the spatial distribution of inflammatory signals, we mapped ISG15 and JAK-STAT signaling scores^34^ to single-cell UMAP projections. Elevated ISG15 and JAK-STAT signaling scores were detected across diverse cell clusters (Fig. 7G). Notably, a strong positive correlation between ISG15 and JAK-STAT signaling scores was evident in both immune and non-immune cells, reinforcing their inflammatory roles in ICI-myocarditis (Supplementary Fig. 10C). Additionally, downstream pathways of JAK-STAT signaling, including chemokine signaling and cytokine-cytokine receptor interaction pathways, were significantly upregulated in heart tissues, particularly in ISG15^+^CD8^+^ T cells and macrophages (Supplementary Fig. 10D, E). These findings highlighted the potential role of JAK-STAT pathways in orchestrating the proinflammatory cardiac microenvironment during ICI-myocarditis.

We next performed CellChat analysis to further evaluate the inflammation-associated intercellular communication across cardiac cell types. Interestingly, inflammatory macrophages exhibited strong TNF signaling pathway interactions with ISG15^+^CD8^+^ T cells, CD4^+^ T cells, and non-immune cells (Fig. 7H, Supplementary Fig. 11A-B). Ligand-receptor interactions were further analyzed across immune and non-immune cells, revealing extensive interactions in ICI-affected hearts but not in controls (Supplementary Fig. 11C-D). The dominant cytokine/chemokine-receptor pair was CCL2-CCR2 (Fig. 7I). Consistent with recent studies^35,36^, CCL2 was mainly located on immune cells, predominantly macrophages, indicating their role in ISG15^+^CD8^+^ T cell recruitment (Fig. 7J).

### JAK-STAT inhibition alleviated myocarditis and enhanced anti-PD-1 efficacy in vivo

**Fig. 8:**
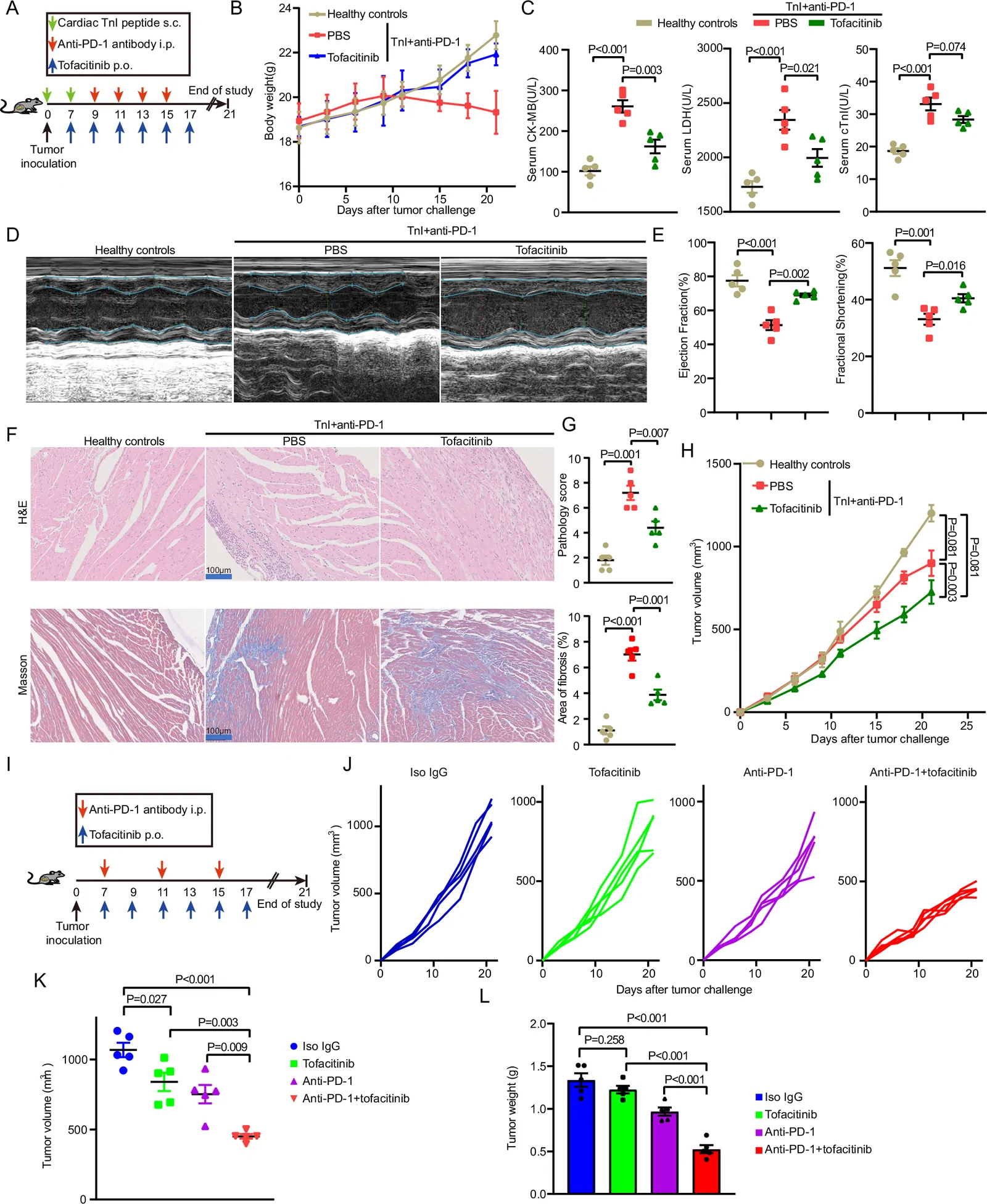
JAK-STAT inhibition ameliorates cardiac inflammation and enhances anti-PD-1 response in ICI-myocarditis model. **A** Treatment diagram for MC38 tumor-bearing mice induced with ICI-myocarditis by cardiac TnI and anti-PD-1 injection. **B** Body weight surveillance during the experiment described above (*n* = 5). **C** Serum levels of CK-MB, LDH, and cTnI in mice (*n* = 5). Two-sided unpaired Student’s *t*test was performed. **D**,**E** Representative photographs of echocardiography (**D**) and quantification of cardiac properties and output (**E**) in echocardiography test (*n* = 5). Two-sided unpaired Student’s *t*test was performed. **F**,**G** Representative images of H&E staining and Masson staining in mouse hearts from each group and their statistical analysis (*n* = 5). Two-sided unpaired Student’s *t*test was performed. **H** Tumor growth curves in MC38 tumor-bearing mice (*n* = 5). Two-sided unpaired Student’s *t*test was performed. **I** Schematic of treatment protocol for MC38 tumor-bearing mice receiving tofacitinib and anti-PD-1 therapy in the absence of myocarditis induction. **J**,**K** Individual growth curves (**J**) and endpoint tumor volume (**K**) for each treatment group (*n* = 5). Two-sided unpaired Student’s *t*test was performed. **L** Tumor weights of MC38 tumor-bearing mice at endpoint across treatment groups (*n* = 5). Two-sided unpaired Student’s *t*test was performed. Iso IgG, Isotype control IgG; TnI troponin I; anti-PD-1, anti-programmed cell death protein 1 antibody. Box plots depicted the interquartile range (box), median (center line), and full data range (whiskers). Mean ± SD were shown where appropriate.

To explore the therapeutic potential of targeting JAK-STAT pathway pharmacologically, we investigated the effects of tofacitinib (Fig. 8A), a first-generation pan-JAK inhibitor clinically approved for rheumatoid arthritis, psoriatic arthritis, and ulcerative colitis^37,38^. Our findings in a mouse model of ICI-myocarditis demonstrated that mice receiving tofacitinib exhibited a notable increase in body weight compared to those in the control group (Fig. 8B). The serum levels of CK-MB, LDH, and cTnI were significantly reduced in the tofacitinib group (Fig. 8C). At day 21, tofacitinib resulted in a significant increase in ejection fraction and fractional shortening (Fig. 8D, E). Furthermore, tofacitinib effectively alleviated inflammatory infiltration and fibrosis in cardiac tissues in vivo (Fig. 8F, G).

In addition to their anti-inflammatory effects, JAK inhibitors, such as ruxolitinib^39^, have been reported to enhance the efficacy of ICI therapy in patients with non-small cell lung cancer. Inspired by these findings, we investigated whether tofacitinib’s mitigation of cardiac inflammation might compromise the antitumor efficacy of ICIs. Surprisingly, we found that tofacitinib did not impair tumor control and instead appeared to modulate tumor growth (Fig. 8H). To better depict the implications of tofacitinib on anti-tumor activity, we established the MC38 tumor-bearing mouse model in the absence of ICI-myocarditis(Fig. 8I). Strikingly, while tofacitinib alone had no significant impact on tumor growth, anti-PD-1 showed enhanced efficacy when combined with tofacitinib (Fig. 8J–L). These findings demonstrated that tofacitinib not only alleviated ICI-myocarditis but also enhanced the antitumor efficacy of anti-PD-1 therapy in this model.

## Discussion

ICIs have revolutionized oncology by harnessing the immune system to target and eliminate tumor cells. However, these agents can trigger a spectrum of irAEs, which can adversely affect patient health and restrict the utility of ICIs in clinical practice^40,41^. Among these irAEs, myocarditis is relatively uncommon but potentially fatal, often leading to significant cardiac dysfunction and, in severe cases, death^42^. Despite growing awareness of this serious complication, the pathogenesis of ICI-myocarditis remains incompletely characterized. In this study, we used an integrative approach combining scRNA-seq, flow cytometry, Olink proteomics, multiplex cytokine assays, and mouse models to identify pathogenic immune signatures, define critical signaling pathways, and explore an effective therapeutic intervention for ICI-myocarditis.

T cells have been recognized as a central therapeutic target in myocarditis, and clinical management frequently involves immunosuppressive strategies aimed at modulating T-cell responses^3,9,22^. Our study revealed that multiple CD8^+^ T cell subsets, including GZMK⁻GZMB^+^, ISG15^+^, and proliferating CD8^+^ T populations, were disproportionately expanded in patients with active ICI-myocarditis. A key finding of our work is the identification and characterization of an ISG15^+^ CD8^+^ T cell population selectively enriched in patients with ICI-myocarditis. These cells exhibited a transitional exhaustion phenotype, suggesting they have been chronically stimulated yet retain the capacity for pathogenic effector function^43^. Similar transitional or “intermediate” exhaustion states have been observed in other contexts of chronic immune activation, such as chronic viral infections and some autoimmune disorders^12,44,45^. Our data specifically link this exhausted-like phenotype to clinical disease severity, reinforcing the notion that T cell phenotypic heterogeneity is a fundamental determinant of tissue-specific toxicities under ICI therapy.

Mechanistically, ISG15^+^CD8^+^ T cells exhibit an upregulation of the canonical JAK-STAT signaling components. ISG15 is a canonical interferon-stimulated gene (ISG) induced downstream of type I interferon signaling via the JAK-STAT pathway^46^. Its expression therefore reflects a well-established interferon-driven immune activation program rather than an unexpected molecular feature. In T cells, type I interferon signaling plays an important role in shaping CD8⁺ T cell activation and differentiation, often accompanied by induction of ISGs. Indeed, ISG15 upregulation has been widely reported in immune cells under conditions characterized by strong interferon responses, including viral infection, autoimmune-associated dermatomyositis, and ICI-induced adverse events following cancer immunotherapy^12,23,24,47^. In our study, we isolated mouse CD8⁺ T cells and stimulated them with r-mIFN-α2 in vitro, which resulted in a marked increase in the proportion of ISG15⁺ CD8⁺ T cells. Importantly, functional coculture assays demonstrated that ISG15⁺ CD8⁺ T cells significantly increased cardiomyocyte apoptosis, indicating direct pathogenic potential. From this perspective, the enrichment of JAK-STAT signaling within the ISG15⁺ CD8⁺ T cells observed in our study is biologically consistent with the canonical interferon-ISG axis. Notably, a recent single-cell study in Epstein-Barr virus (EBV)-associated gastric cancer has identified ISG15⁺ CD8⁺ T cells enriched in immunotherapy-responsive tumors^20^. In a recent study, Blum et al. identified cytotoxic CD8⁺ T cells in cardiac tissues as key mediators of ICI-myocarditis^11^. In contrast, our study focuses on peripheral blood and reveals an expansion of ISG15⁺ CD8⁺ T cells with a prominent JAK-STAT-driven program. These differences likely reflect complementary aspects of disease biology. Tissue-resident cytotoxic CD8⁺ T cells may represent terminal effectors, whereas peripheral ISG15⁺ CD8⁺ T cells may capture a systemic immune activation state. In addition, variation in disease stage, sample source, and analytical approaches may also work. Notably, reanalysis of the published dataset from Blum et al.^11^ confirmed the presence of ISG15⁺ CD8⁺ T cells in myocarditis, supporting the relevance of this state across compartments.

The JAK-STAT pathway orchestrates diverse immune responses, including cytokine signaling, immune-cell survival, differentiation, and effector function, and is frequently associated with inflammation when dysregulated^48^. These findings align with evidence from studies of ICI-induced colitis, where tissue-resident memory CD8^+^ T cells strongly express checkpoint protein, and serve as key effector populations^49^. Notably, a single-cell analysis of ICI-associated colitis has demonstrated consistent tissue-resident memory CD8^+^ T cell activation through JAK-STAT signaling, suggesting inhibition of JAK-STAT signaling as a promising therapeutic approach. In line with these findings, we demonstrate that tofacitinib effectively attenuates cardiac inflammation and improves cardiac function in mouse models. A major concern regarding JAK inhibition in cancer patients is the possibility of impairing antitumor immunity, since this pathway also participates in immune regulatory circuits and, in certain contexts, has been implicated in immune escape mechanisms, such as PD-L1 upregulation in tumor cells^50–52^. Therefore, therapeutic inhibition of JAK-STAT signaling in cancer patients requires careful consideration of both potential benefits and risks. Emerging clinical and experimental evidence suggests that this risk may be context-dependent. For example, two recent independent studies have demonstrated that JAK inhibitors can enhance ICI therapy in patients with non-small cell lung cancer^39^ and Hodgkin lymphoma^53^, respectively. Motivated by these data, we evaluated whether the anti-inflammatory effects of tofacitinib might diminish the antitumor activity of ICIs. Contrary to concerns, we observed that tofacitinib did not compromise tumor control and even appeared to enhance tumor control in the MC38 model in the absence of myocarditis, highlighting the translational potential of JAK-STAT inhibition as a promising targeted therapy for this life-threatening complication. Nevertheless, JAK-STAT inhibition should not be viewed as a broadly applicable adjunct to immunotherapy. The effects of JAK inhibitors are likely dependent on cancer type, timing, and dosing of ICI therapy^54,55^.

In parallel to these mechanistic discoveries, our integrated analysis allowed us to construct an 8-protein signature for the identification of ICI-myocarditis. This signature, characterized by enhanced inflammatory and chemotactic proteins, demonstrated robust predictive performance in both training and validation cohorts. Current methods for diagnosing ICI-myocarditis, including echocardiography and troponin-based tests, can sometimes be non-specific, underscoring the potential utility of a molecular biomarker panel for more timely and accurate intervention^56,57^. The potential clinical value of this panel lies in its ability to facilitate earlier risk stratification among ICI-treated patients, a setting where delayed diagnosis can result in irreversible myocardial injury^58^. From a practical standpoint, the panel is based on serum biomarkers, which can be obtained through routine blood sampling and are therefore minimally invasive and readily compatible with standard oncology workflows. The multiplex Luminex platform allows simultaneous measurement of multiple analytes with relatively low sample volume and reasonable cost^59^. As such, this approach is well suited for repeated measurements and longitudinal monitoring. Of note, this panel would not replace the current diagnostic standards, such as cardiac biomarkers, echocardiography, or cardiac MRI, but rather serve as a complementary tool. In particular, it could function as an early screening or triage assay to identify ICI-treated patients at high risk for ICI-myocarditis, prompting intensified cardiac surveillance, expedited imaging, or earlier therapy for ICI-myocarditis. In addition, validation in independent cohorts with diverse irAEs and cardiac diseases will be essential to further define its diagnostic specificity. In particular, testing the panel in patients with non-cardiac irAEs would help determine whether the 8-protein panel is myocarditis-specific or reflects a more general immune activation state, and we consider this an important direction for future prospective studies.

An additional dimension of our study focused on delineating the intercellular communication networks that contribute to myocardial injury. Ligand-receptor interaction analyses revealed that ISG15^+^CD8^+^ T cells and monocytes engaged in robust crosstalk via the CCL2-CCR2 axis. This chemokine signaling axis, classically implicated in the recruitment of monocytes and other immune cells to sites of inflammation, has emerged in recent years as a pivotal mediator of tissue damage in various inflammatory settings^60–62^. A further avenue for future research involves assessing whether the ISG15^+^CD8^+^T cells' transitional exhaustion state or the CCL2-CCR2 axis is relevant to other forms of irAEs beyond myocarditis. Studies of ICI-induced pneumonitis, hepatitis, nephritis, and colitis have begun to reveal parallels in the underlying immunopathology^63,64^. Specifically, hyperactivated T-cell subsets interacting with proinflammatory myeloid compartments appear to be a recurring theme. Should these pathogenic circuits prove consistent across various tissues, a pan-irAE therapeutic strategy targeting JAK-STAT signaling and associated chemokine networks might be envisaged. This would markedly simplify clinical management of irAEs, sparing patients multiple lines of immunosuppressive regimens that often carry significant infection risk and other side effects.

Our findings also align with the expanding literature on type I interferons, an area that has seen significant focus in autoimmune-like conditions, such as MDA5^+^ dermatomyositis^12^. Similar to MDA5^+^ dermatomyositis, where overactivation of type I and II interferons has been implicated in driving lung fibrosis and other organ complications, ICI-myocarditis exhibits overlapping immunological signatures^65,66^. In both conditions, unchecked IFN signaling fosters hyperinflammation, fueling tissue damage. By pinpointing overactive IFN pathways, we emphasize the clinical value of JAK inhibitors, which simultaneously block both types I and II interferon signals, as a targeted solution for conditions arising from dysregulated interferon responses.

In summary, our study provides a comprehensive immunologic landscape of ICI-myocarditis and identifies cellular and molecular features related to disease activity. These insights may improve our understanding of the pathogenesis of ICI-myocarditis, providing clues for biomarkers and therapeutic targets that preserve antitumor immunity.

## Methods

### Study design and patient population

We consecutively enrolled adult cancer patients who received anti-PD-1, anti-PD-L1, anti-CTLA-4, or combination ICI therapies at Zhongshan Hospital, Fudan University, between September 30, 2017 and December 31, 2024, and identified patients diagnosed with ICI-related myocarditis. Patients were reviewed for a clinical diagnosis of ICI-related myocarditis, defined according to the criteria of Bonaca et al^4^. The first and senior authors undertook a second round of review to adjudicate all patients identified as having ICI-myocarditis to ensure adherence to the defining criteria. The Bonaca definition was applied as the main diagnostic definition because it was the principal diagnostic framework during the study period. Although it uses similar variables to the subsequent International Cardio-Oncology Society definition^67^, it differs in that it does not require a troponin elevation for diagnosis (and so may capture cases after the troponin rise if they are manifested subacutely) and has sub-categories.

The follow-up period ended on May 1, 2025. Inclusion criteria were: (1) individuals aged 18 years or older at myocarditis onset; (2) histological or imaging-based confirmation of malignancy; (3) administration of at least one cycle of ICI therapy; and (4) evidence supporting a diagnosis of ICI-myocarditis, based on myocardial biopsy, biochemical indicators, clinical symptoms, or cardiac magnetic resonance imaging (CMR) findings. Exclusion criteria encompassed: (1) those who had an elevation of the CK-MB and/or troponin I/T due to other cardiovascular diseases; (2) active severe infections; (3) chronic infectious conditions such as HIV or tuberculosis; and (4) concurrent cardiovascular or pulmonary diseases including acute coronary syndrome, viral myocarditis, pericarditis, or pulmonary embolism. The degree of myocarditis was classified according to the Common Terminology Criteria for Adverse Events (CTCAE), version 5.0.

The present study was designed to profile transcriptomic changes in PBMC samples of ICI-myocarditis. PBMC samples were collected from the following groups for single-cell RNA sequencing: (1) ICI-treated patients with non-myocarditis irAE (no-ICIAM, *n* = 6); (2) patients during active ICI-related myocarditis onset (act-ICIAM, *n* = 10); and (3) patients with ICI-related myocarditis sampled during disease remission (rem-ICIAM, *n* = 3). For act-ICIAM patients, they provided PBMC samples prior to the initiation of any corticosteroid or immunosuppressive treatment. Remission samples were collected after clinical remission. Patients’ demographic information, laboratory data, clinical manifestations, and treatments were recorded in Supplementary tables. This study was approved by the Institutional Review Board of Zhongshan Hospital, Fudan University (B2022-444). Written informed consent was obtained from all participants. All procedures adhered to the ethical principles in the Declaration of Helsinki.

### Sample processing and preparation

For PBMC isolation, whole blood samples were diluted 1:1 with phosphate-buffered saline (Biosharp) then layered over lymphocyte separation solution (Ficoll-Paque PREMIUM) in 50 mL centrifuge tubes. Centrifugation was performed at 2000 g for 20 min at 20 °C with the brake disabled in a horizontal rotor. PBMCs were collected from the PBS-Ficoll-Paque interlayer and lysed red blood cells with red blood cell lysis buffer (Sangon Biotech) for 5 min at room temperature, then washed twice in PBS. The freshly prepared cells were then used in subsequent assays for further analysis.

### Single-cell RNA sequencing

Single-cell suspensions were loaded onto a Chromium Single Cell Controller (10x Genomics) to generate Gel Bead-In-Emulsions (GEMs). Single-cell 3’ gene expression libraries were prepared by strictly following the protocols of a Chromium Single Cell 3’v2 Library kit (10x Genomics). All resulting libraries were sequenced on the Illumina NovaSeq 6000 PE150 platform (Dunwill Medical, Shanghai, China).

### Data alignment and quality control

Raw data were assembled into FASTQ files using Illumina bcl2fastq. After initial quality control, adapter sequences, sequences with over three uncertain nucleotides (designated as N), and low-quality reads were removed. To filter cells with low quality, we used the following selection criteria: cells with a gene number greater than 200 and less than 4000 and a mitochondrial DNA percentage lower than 10%, and genes with at least one feature count in more than 5 cells were selected for subsequent analysis.

### Single-cell transcriptome data processing

All single-cell transcriptome sequencing data were aligned and quantified by using CellRanger (v7.1.0) against the GRCh38 human reference genome from 10x Genomics official website. Unique molecule identifiers (UMIs) for each gene and each cell barcode were counted and added to gene expression matrices. scRNA-seq expression matrices were processed using Seurat (v4.0.1)^68^. Then, DoubletFinder (v2.0.3)^69^ was used to identify and remove the potential doublets. The standard of identifying the doublet cells for each single cell was based on the bimodal distribution by using the default parameters.

### Dimensionality reduction and annotation of major cell clusters

We performed data normalization by employing a global-scaling normalization method “LogNormalize” that normalized the feature expression measurements for each cell by the total expression, multiplied this by a scale factor of 10,000, and log-transformed the result. The batch effects in the study samples were corrected by the RunHarmony function in the Harmony (v2.0.5). The t-distributed stochastic neighbor embedding (t-SNE) or uniform manifold approximation and projection (UMAP) method was applied to visualize a 2D projection of identified clusters from the shared nearest neighbor (SNN) graph. Cells were clustered with highly variable genes at a resolution of 0.6. The top 30 differentially expressed genes (DEGs) of each cluster were identified and carefully examined for established canonical marker genes. Based on the marker genes of each cluster, two researchers manually annotated the cell clusters and rechecked them independently.

### Differential gene expression and functional enrichment analysis

To elucidate the differences in gene expression profiles among cell clusters and between different groups, we performed the FindAllMarkers function (Seurat) to identify DEGs, which were defined as genes with |log₂fold change | > 2.0 and adjusted *P* < 0.05.

To annotate the function of these DEGs, we performed the Gene Ontology (GO) Biological Process and Kyoto Encyclopedia of Genes and Genomes (KEGG) pathway enrichment analysis by using the ClusterProfiler (v3.19.0) following the default parameters (datasets: org.Hs.eg.db v3.13.0)^70^. Gene set enrichment analysis (GSEA) was performed using the canonical pathways in curated gene sets and hallmark gene sets in the Molecular Signatures Database (MSigDB) to identify the enriched pathways between different groups. Pathway activity scores were calculated at the single-cell level based on the expression of the MSigDB gene sets using the AUCell package (v1.14.0)^71^.

### Pathway and signature enrichment analysis

We employed the GSVA package (v1.50.5)^72^ to illustrate pathway activity in T-cell and myeloid subpopulations using the Hallmark and C2 gene collections from the MSigDB database. Differential pathway enrichment scores were estimated by applying a linear model through the limma package. In addition, we used the ClusterProfiler (v3.19.0) to calculate the distribution of gene sets in lists of genes ordered by population expression differences, thereby pinpointing signatures that were markedly enriched in specific immune cell subsets.

### Definition of signature gene score

We calculated naiveness, cytotoxicity, and dysfunction scores using AUCell (v1.14.0)^71^ for the following genes commonly reported in studies^73–75^ related to each state: six naïve markers (TCF7, CCR7, SELL, LEF1, IL7R, LTB), six dysfunction-associated genes (PDCD1, CTLA4, TIGIT, HAVCR2, LAG3, LAYN), and eight cytotoxic markers (CX3CR1, PRF1, GZMA, GZMB, GZMH, GNLY, KLRG1, NKG7). For each cell, AUCell ranks gene expression and calculates an AUC value that reflects how many genes from the specified set are highly expressed in that cell. Consequently, cells with higher relative expression of a given set receive elevated AUC values. We then used the AUCell_exploreThresholds function to determine the point at which a cell is considered to be “active” for a particular signature. Finally, we visualized these results on the UMAP embedding by coloring cells according to their AUC values, thereby highlighting clusters enriched in each signature^76^.

### Cell-cell interaction analysis

We utilized the CellChat package (v1.6.1) to infer intercellular communication and identify significant ligand-receptor pairs involved in the immunopathogenesis of ICI-myocarditis using a standard pipeline. To ensure statistical significance, probability values for each interaction were calculated through permutation tests. The inferred intercellular communication network for each ligand-receptor pair and signaling pathway was summarized and visualized with circle plots and heatmaps.

### Trajectory analysis

To investigate potential differentiation pathways among CD8^+^ T cells, we conducted trajectory analysis via the monocle package (v2.22.0)^77^. For monocle trajectory inference analysis, we created the monocle object using the “newCellDataSet” function, selecting DEGs calculated via the “differentialGeneTest” function for further trajectory analysis. Then, dimensionality reduction was achieved using the “DDRTree” function, and visualization was performed with the “plot_cell_trajectory” function. Branching lineages in the cell types were inferred separately, considering naïve T cells as the root state for calculating pseudotime and trajectories.

### External validation analysis for independent scRNA‑seq and bulk RNA‑seq datasets of cardiac tissue

To corroborate our findings, we retrieved single‑cell RNA‑seq datasets (GSE227437 and GSE228597) from the GEO database. Raw count matrices were imported into Seurat (v4.0.1) as individual Seurat objects. After standard quality filtering and log‑normalization, we integrated all samples with Harmony (v2.0.5) to correct batch effects^78^. Downstream analyses, including unsupervised clustering, identification of differentially expressed genes, gene‑signature scoring, and pseudotime trajectory reconstruction, were carried out following the workflows described above.

To extend these observations to human disease, we also analyzed the microarray expression data of human cardiac tissues from the GSE57338 dataset. Expression matrices were processed using the limma pipeline for differential gene expression, and DEGs were subjected to pathway enrichment analysis to assess concordance with single‑cell results.

### Flow cytometry

PBMCs were initially stained with Fixable Viability Stain 510 (BD Biosciences, 564406) according to the manufacturer’s instructions to discriminate and remove nonviable cells. After washing, cells were incubated with human Fc receptor blocking reagent (STARTER, S0F0008) and stained with fluorochrome-conjugated antibodies against surface markers in staining buffer (PBS supplemented with 5% FBS, 2 mM EDTA and 0.09% sodium azide) for 15 min at room temperature. Subsequently, cells were fixed and permeabilized using the Cytofix/Cytoperm Fixation/Permeabilization Kit (BD Biosciences, 554714), then stained intracellularly with the relevant fluorophore-tagged antibodies in permeabilization buffer for 30 min at 4 °C. Data were acquired using a BD LSRFortessa flow cytometer and analyzed using FlowJo software (v10.0.7).

The antibodies used in this study are listed as follows: APC anti-human CD8 (BD Pharmingen, 566852), APC-Cy7 anti-human CD3 (BD Pharmingen, 557757), FITC anti-human CD4 (BD Pharmingen, 555346), and PE-conjugated anti-human ISG15/UCRP antibody (R&D Systems, IC8044P).

### Olink proteomics analysis

The Olink® target 96 inflammation panel (OLINK Proteomics AB, Uppsala, Sweden) was used to quantify protein levels. Sample processing and data collection were performed by Sinotech Genomics Co., Ltd. (Shanghai, China). The details of the proximity extension assay are available from the manufacturer’s website (www.olink.com). Wilcoxon rank-sum tests were employed to identify differentially expressed proteins, with a significance threshold of P < 0.05. Functional annotation of significant proteins was then performed through GO and KEGG pathway enrichment analysis.

### Multiplex cytokine assay

Serum was separated from whole blood samples by centrifugation at 3000 × g for 10 min and stored at -80 °C. Concentrations of the following cytokines and chemokines were measured using the multiplexed Luminex assay in accordance with the recommendations of the manufacturer(R&D Systems, LXSAHM-14). Concentrations were determined using Luminex Xponent software (v4.3), with samples measured in duplicate and the concentrations subsequently averaged. Samples exhibiting a coefficient of variation value > 30% were eliminated. For cytokines/chemokines with concentrations below the lower limit of quantification, values were set to 50% of that limit. Cytokines/chemokines present in at least half of the samples in at least one subgroup were included in the analysis. The selected cytokines/chemokines included IL-6, CXCL9, MMP-1, CCL2, HGF, IFN-γ, TRANCE, and TNFB.

### ICI-myocarditis model and treatments

Female C57BL/6 mice (6-8 weeks old, 18–20 g) were obtained from SLAC Laboratory Animal Co., Ltd (Shanghai, China). All animal care and protocols were conducted according to the guidelines of Institutional Laboratory Animal Care and Use Committee of the Zhongshan Hospital, Fudan University (No. 202503004S). Only female mice were included in this study due to vivarium constraints. Sex was deemed non-critical to the research objectives, as the investigated therapeutic targets are not typically subject to sex-specific regulation.

To establish an in vivo model of ICI-myocarditis, mice were immunized subcutaneously on day 0 and 6 with 0.1 mL mouse cardiac troponin I (cTnI) peptide 250 µg dissolved in complete Freund’s adjuvant (Sigma-Aldrich, USA)^79,80^. Starting on day 4, mice were given an intraperitoneal injection of anti-PD-1 antibody (BioXCell, BE0146) every 2 days at a dose of 250 μg per mouse for six times. During the model establishment, RS504393 (MedChemExpress, HY-15418) was administered at 6 mg/kg by intraperitoneal injection every 2 days for a total of eight doses to inhibit CCL2-CCR2 axis in vivo.

### Blocking JAK-STAT signaling pathway in the ICI-myocarditis model

To investigate the role of JAK-STAT signaling in the ICI-myocarditis, ICI-myocarditis mice received tofacitinib (MedChemExpress, HY-40354) at a dose of 10 mg/kg via oral gavage every 2 days for a total of six doses to block JAK-STAT signaling pathway in vivo.

### Tumor models and treatments

Female C57BL/6 mice (6–8 weeks, 18–20 g) were inoculated subcutaneously in the right flank with 1.0 × 10^6^ MC38 cells in 100 µL serum‑free medium. Tumor dimensions were measured every 3 days with digital calipers, and volume was calculated as (width^2^×length)/2. Once the tumors reached a volume of 50–100 mm^3^, animals were randomized into four groups and treated with isotype IgG control (200 µg/mouse, BE0089), anti-PD-1 antibody (200 µg/mouse, BioXCell, BE0146), tofacitinib (10 mg/kg, MedChemExpress, HY-40354), or anti-PD-1 antibody combined with tofacitinib. After tumor inoculation, isotype IgG or anti-PD-1 were intraperitoneally injected every 4 days from day 7 for three times, while tofacitinib was administered via oral gavage every 2 days for six times. Tumor growth and survival outcomes were then monitored to evaluate treatment efficacy. Mice were euthanized when tumors exceeded 2,000mm^3^, a threshold that was not reached by any animal in this study.

### Serum markers of myocardial injury

Serum levels of myocardial injury markers, including creatine kinase-MB (CK-MB) and lactate dehydrogenase (LDH), were measured using commercially available assay kits (Nanjing Jiancheng BioTech, China) following the manufacturer’s protocols. Cardiac troponin I (cTnI) concentrations were quantified using an ELISA kit (Elabscience Biotechnology, China) in accordance with the manufacturer’s instructions.

### Histological analysis of hearts

Heart tissues were collected, and fixed in 4% paraformaldehyde, embedded in paraffin, and sectioned for histological examination. Paraffin-embedded cardiac sections were subjected to Masson’s trichrome staining to assess fibrosis, where collagen fibers were visualized in blue and myocardial fibers in red. Hematoxylin and eosin (H&E) staining was used to examine overall tissue architecture, including inflammatory infiltration and necrotic damage. Stained sections were visualized and imaged using a light microscope (NanoZoomer S210, Hamamatsu, Japan).

### Mouse splenic CD8⁺ T cell isolation and culture

Mouse splenic CD8⁺ T cells were isolated using a mouse CD8⁺ T cell isolation kit (BioLegend, 480008) according to the manufacturer’s instructions. The sorted CD8⁺ T cells were cultured ex vivo in RPMI-1640 medium (Corning, 10-040-CVR) supplemented with 10% fetal bovine serum, 12.5 mM HEPES (Gibco, 15630-080), 5% sodium pyruvate (Gibco, 11360-070), 5% penicillin-streptomycin-amphotericin B, 5% non-essential amino acids (Gibco, 11140-050) and 100 U/mL recombinant mouse IL-2 (Novoprotein, CK24).

### In vitro stimulation of CD8^+^ T cells

For T cell activation, culture plates were pre-coated with anti-mouse CD3ε antibody (BioLegend, 100340), and soluble anti-mouse CD28 antibody (BioLegend, 102116) was added to the culture medium. Cells were stimulated for 3 days at 37 °C with 5% CO₂. On day 3, cells were harvested and washed twice with PBS by centrifugation at 1000 g for 5 min to remove activation stimuli, and then further cultured under the same conditions for expansion, with fresh medium replaced daily. The total culture period was within 6 days and mouse CD8⁺ T cells were collected for further use.

To generate ISG15⁺ CD8⁺ T cells, cultured mouse CD8⁺ T cells were treated with recombinant mouse IFN-α2 (MCE, HY-P73247) at the indicated concentrations for 48 h. Cells were then harvested and washed twice with PBS before subsequent analyses.

### Co-culture and cytotoxicity assays

After cytokine stimulation, the cells were centrifuged and washed three times with PBS and subjected to downstream functional assays. ISG15^+^CD8^+^ T cells were co-cultured with HL-1 cardiomyocytes in different ratios (1:1, 1:5, and 1:10) with respect to cardiomyocyte count. Following co-culture for 24 h, ISG15^+^CD8^+^ T cells were harvested for detecting GZMB expression, whereas cardiomyocytes were harvested for apoptosis, and reactive oxygen species (ROS) production assay. All measurements were performed using flow cytometry.

### Statistical analysis

Comparisons of categorical variables were performed using the chi-squared test or Fisher’s exact test, while continuous variables were analyzed with either the Wilcoxon rank-sum test or the unpaired two-tailed Student’s *t*test, depending on data distribution. Receiver operating characteristic (ROC) analysis was conducted to assess the diagnostic performance of candidate biomarkers, and the area under the curve (AUC) was calculated. A two-sided P value < 0.05 was considered statistically significant. Data analysis and visualization were performed using R software (v4.2.0) and GraphPad Prism (v8.0.1).

### Reporting summary

Further information on research design is available in the Nature Portfolio Reporting Summary linked to this article.

## Supplementary information

Supplementary Information

Reporting Summary

Transparent Peer Review file

## Source data

Source data

## Data Availability

Raw single-cell RNA data generated in this study have been deposited in the National Genomics Data Center with the accession code HRA019659. This study reanalyzed publicly available genomic datasets from the Gene Expression Omnibus (GEO) database, accessible under accession numbers GSE228597, GSE227437, and GSE57338. All other data are available in the article and its Supplementary files or from the corresponding author upon request. Source data are provided with this paper.
