# Supplementary material for: Integrated multi-omics identifies distinct immune signatures with diagnostic and therapeutic implications in immune checkpoint inhibitor-induced myocarditis
Source: Nat Commun. 2026 Oct 14;17:11591. doi: 10.1038/s41467-026-77262-0 (PMC13751616; doi:10.1038/s41467-026-77262-0)
Supplement: Supplementary file 1 [file 41467_2026_77262_MOESM1_ESM.pdf]

Supplementary Information

Supplementary Fig. 1. Quality control and annotation of PBMC single-cell RNA-seq data.

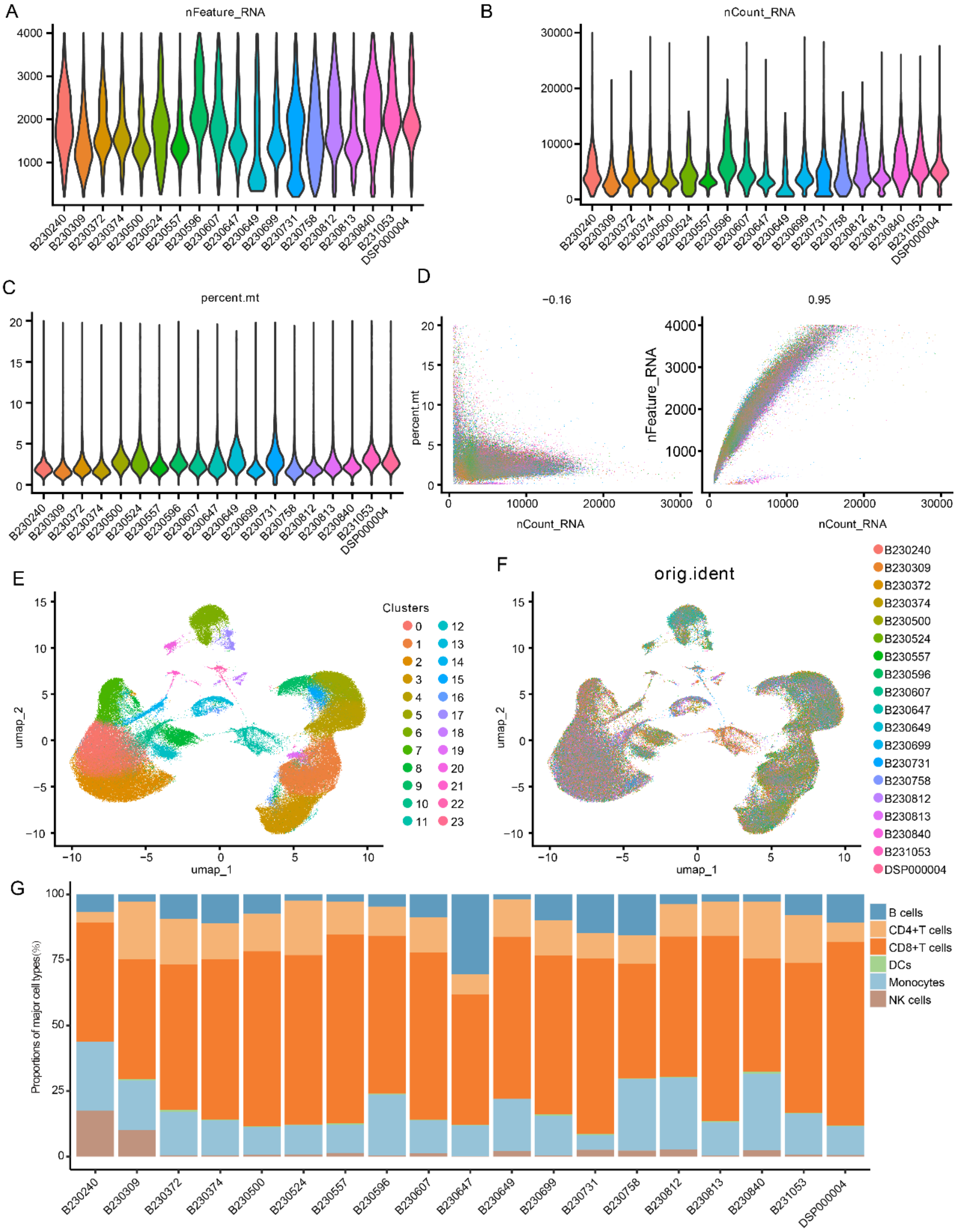

A-C, The genes (A), counts (B), and mitochondrial gene percentage (C) of each sample.

D, Relationship between RNA count and mitochondrial gene percentage in single cells.

E, 199,741 cells from no-ICIAM (n = 6), act-ICIAM (n = 10), and rem-ICIAM (n = 3) human PBMCs are clustered into 24 subpopulations.

F, UMAP plots showing the origins of individual immune cells in the included patients. G,Stacked column charts showing the proportions of indicated clusters in the six major cell types across all samples. Clusters were color-coded.

Supplementary Fig. 2. Dot plots showing the expression of canonical marker genes across different clusters, including $CD8^+$ T cell subclusters (A), and $ISG15^+CD8^+$T cell subclusters (B).

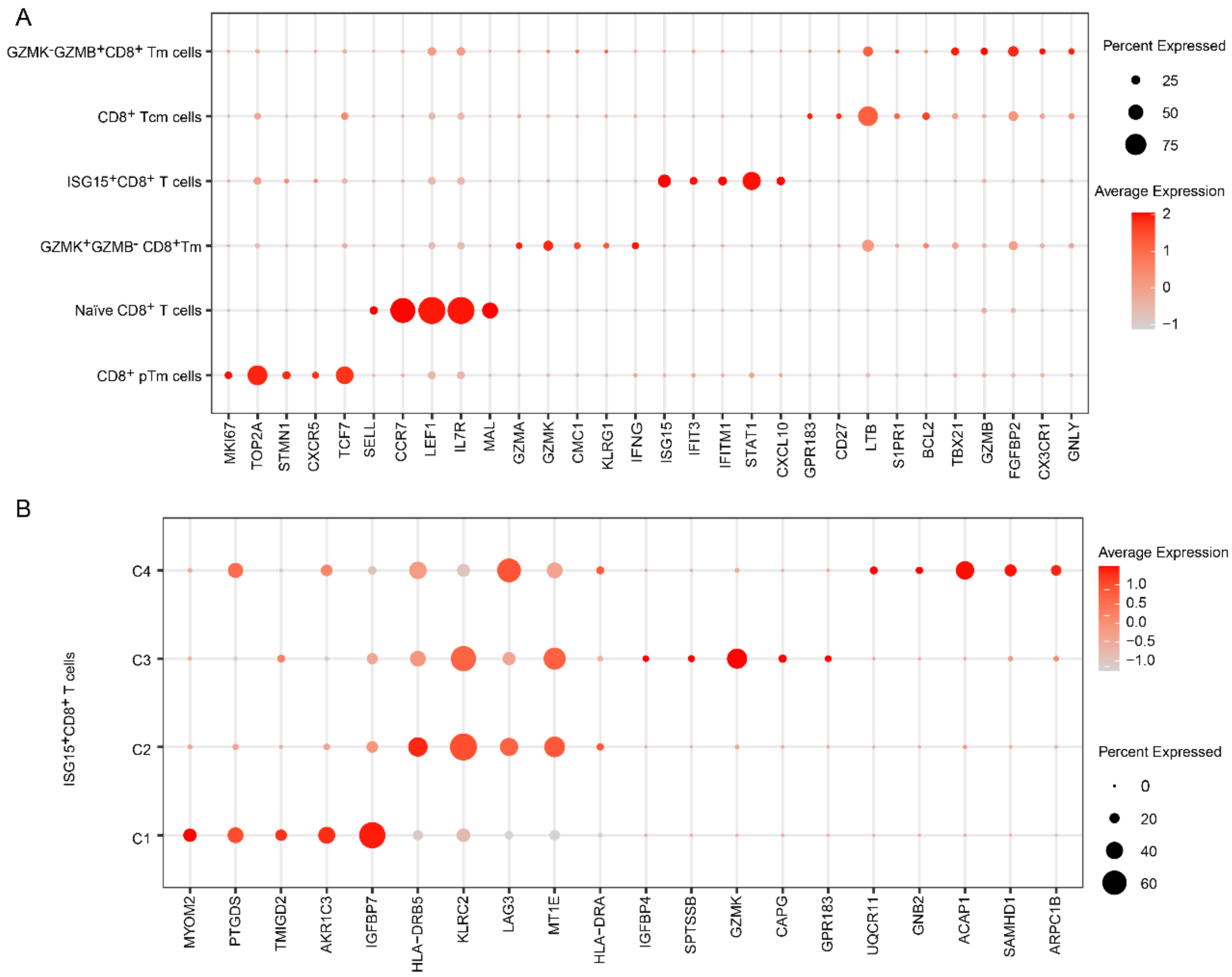

Supplementary Fig. 3. Determinants of act-ICIAM based on peripheral blood profiling.

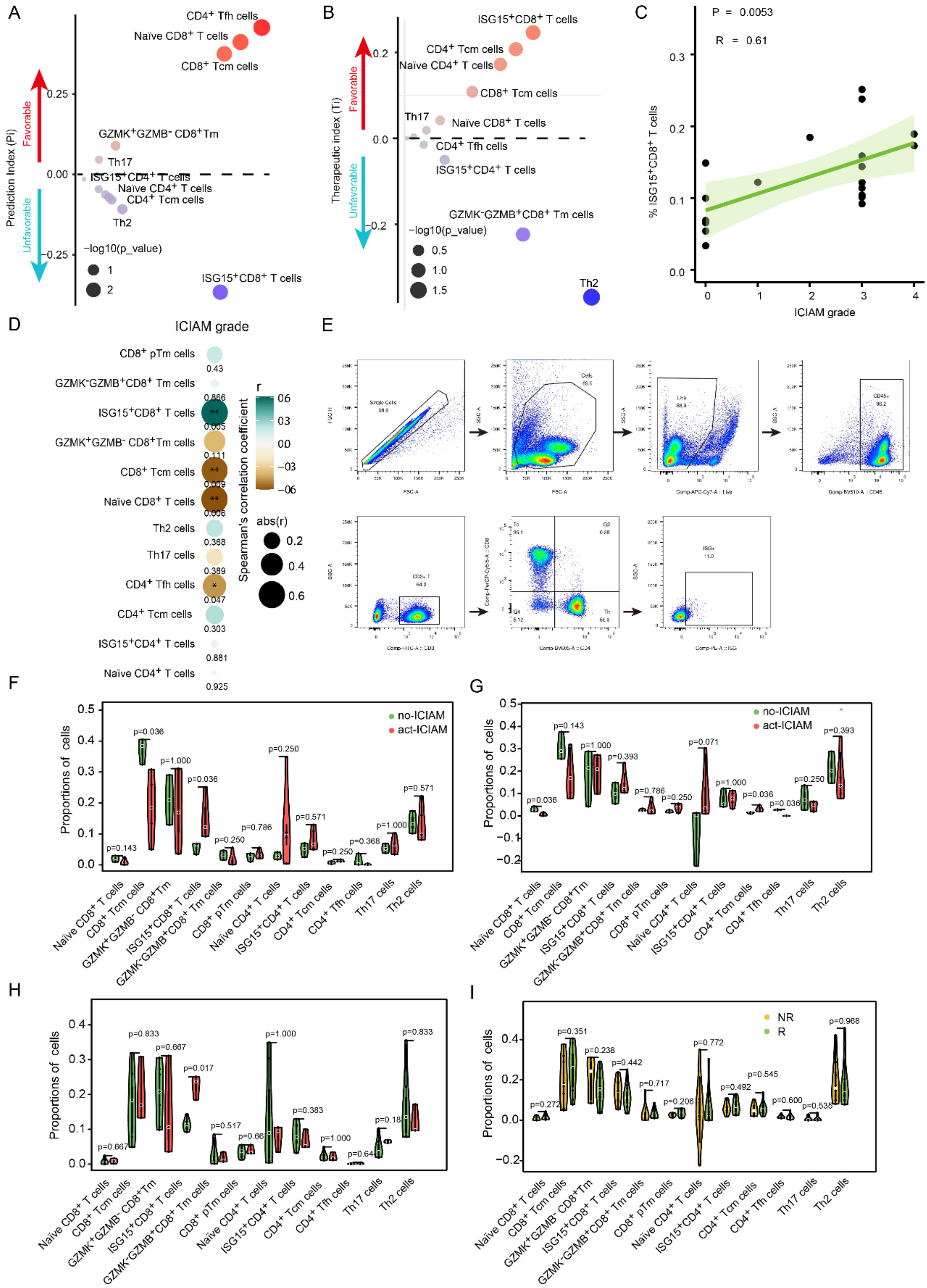

A-B, Predictive index (Pi) and therapeutic index (Ti) for major immune cell types in the scRNA-seq discovery cohort (n = 16). Dot size corresponds to -log$_{10}$(P), and colors denote immune cell subsets.

C, The correlation between the proportion of ISG15^{+} CD8^{+} T cells and ICI-myocarditis

grade, analyzed using Spearman correlation.

D, Dot plot showing the correlation between cell proportion of T cells and ICI-myocarditis grade. Spearman correlation analysis was applied.

E, Flow cytometry gating strategy to determine $ISG15^{+}CD8^{+}T$ cells in peripheral blood.

F, Subgroup analysis according to the cancer type reveals a visible but statistically not significant increase of $ISG15^{+}CD8^{+}T$ cells in patients with gastrointestinal cancer subgroup.

G, Subgroup analysis according to the cancer type reveals a visible but statistically not significant increase of $ISG15^{+}CD8^{+}T$ cells in the non-gastrointestinal cancer subgroup.

H, Subgroup analysis according to non-ICIAM irAEs reveals a visible but statistically not significant increase of $ISG15^{+}CD8^{+}T$ cells.

I, Elevation of $ISG15^{+}CD8^{+}T$ cells in patients with ICI-myocarditisis not associated with a better tumor response. NR, non-responders; R, responders; ICI, immune checkpoint inhibitor; no-ICIAM, no ICI-myocarditis; act-ICIAM, active ICI-myocarditis. Box plots depicted the interquartile range (box), median (centre line), and full data range (whiskers). Mean ± SD were shown where appropriate.

Supplementary Fig. 4. Pseudotime trajectory analysis of CD8⁺ T-cell differentiation states.

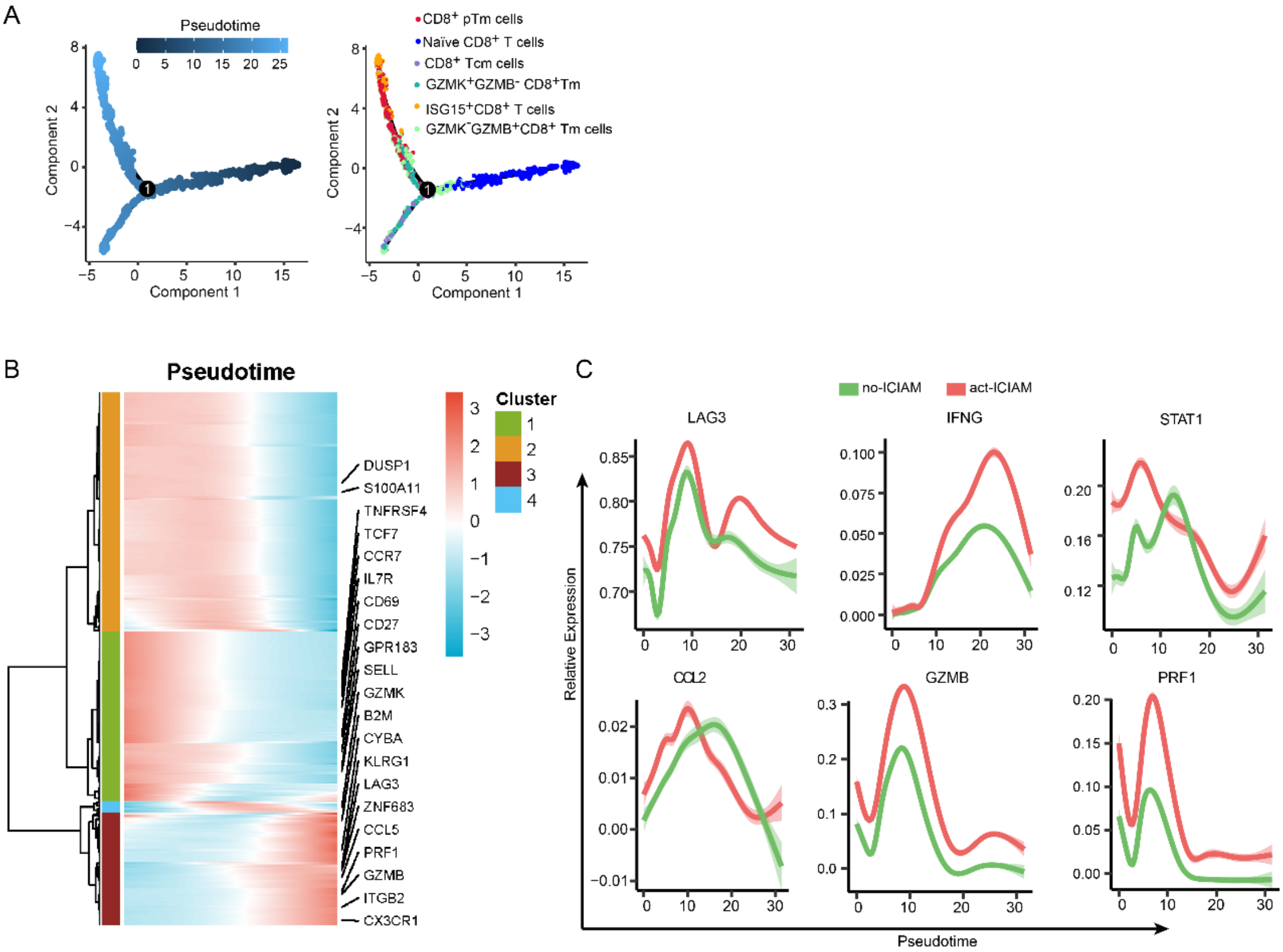

A, Differentiation trajectory of CD8$^+$ T cell subclusters predicted by Monocle2. CD8$^+$ T cell subclusters are labeled by color.

B, Pseudotime expression modules identified by Monocle2 along with the development of CD8$^+$ T cell subclusters fate.

C, Two-dimensional plots illustrating the dynamic expression of genes significantly upregulated along pseudotime in the no-ICIAM and act-ICIAM groups. ICI, immune checkpoint inhibitor; no-ICIAM, no ICI-myocarditis; act-ICIAM, active ICI-myocarditis.

Supplementary Fig. 5. Single-cell transcriptomic analysis of human cardiac tissues in the GSE228597 dataset.

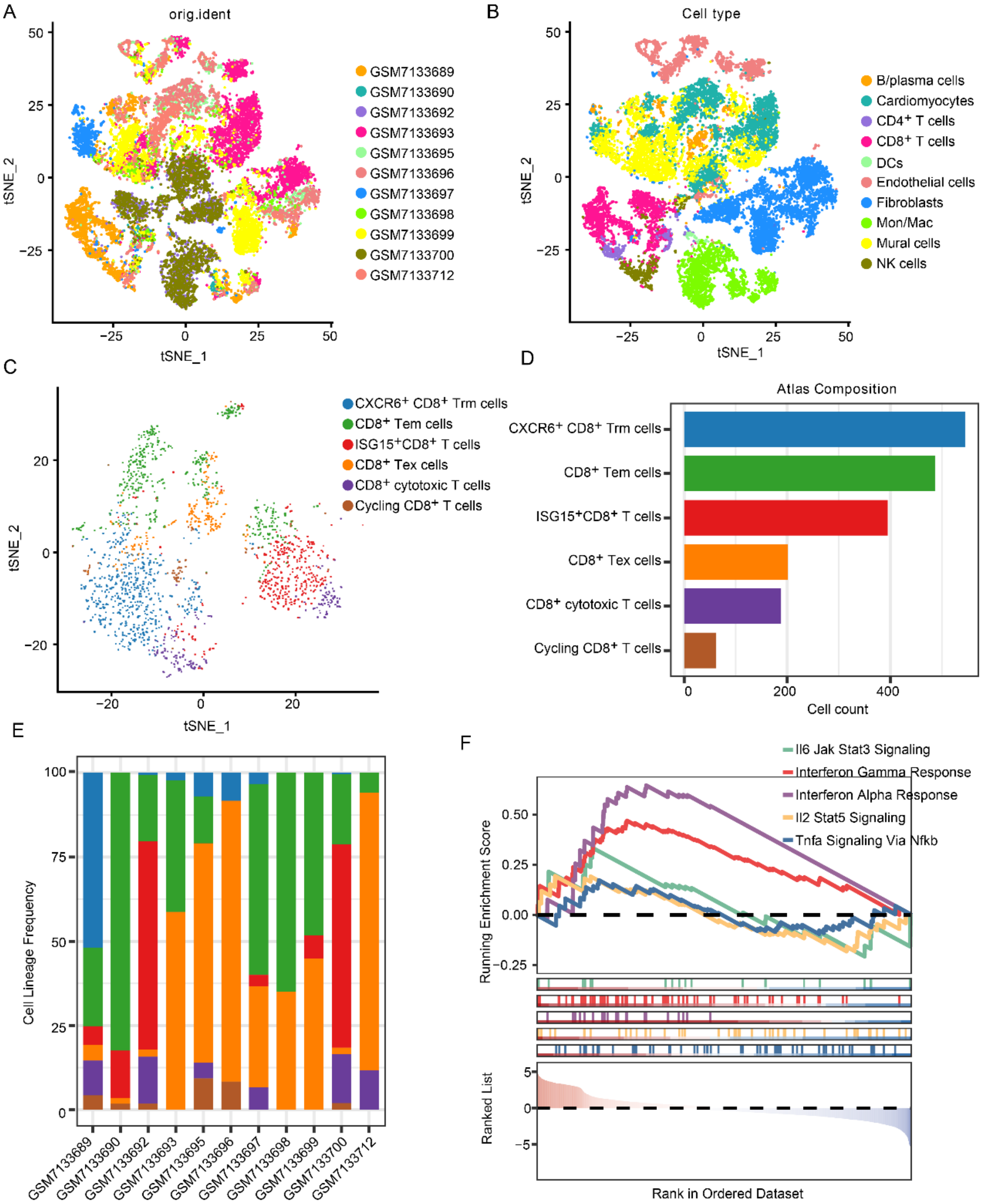

A, t-SNE plot showing the origins of individual immune cells in the cardiac tissues from no-ICIAM and ICIAM patients (n = 11).

B, t-SNE plot showing the overview of 10 cell clusters in the cardiac tissues from no-ICIAM and ICIAM patients (n = 11). Clusters are named according to the indicated immune cell subsets using the canonical marker genes and are color-coded.

C, Clustering of CD8^{+} T cells based on all enrolled samples (n =11), colored and labeled by CD8^{+} T cell clusters.

D-E, Bar plots showing the cell counts for each CD8^{+}T cell lineage (D) and the cell lineage proportions per sample (E).

F, GSEA analysis revealing pathways significantly upregulated in $ISG15^{+}$ $CD8^{+}$ T cells from ICIAM samples compared with no-ICIAM samples. t-SNE, t-distributed stochastic neighbor embedding.

Supplementary Fig. 6. ISG15 induction and cytotoxic effects of ISG15⁺CD8⁺ T cells on cardiomyocytes at a low ratio (1:10).

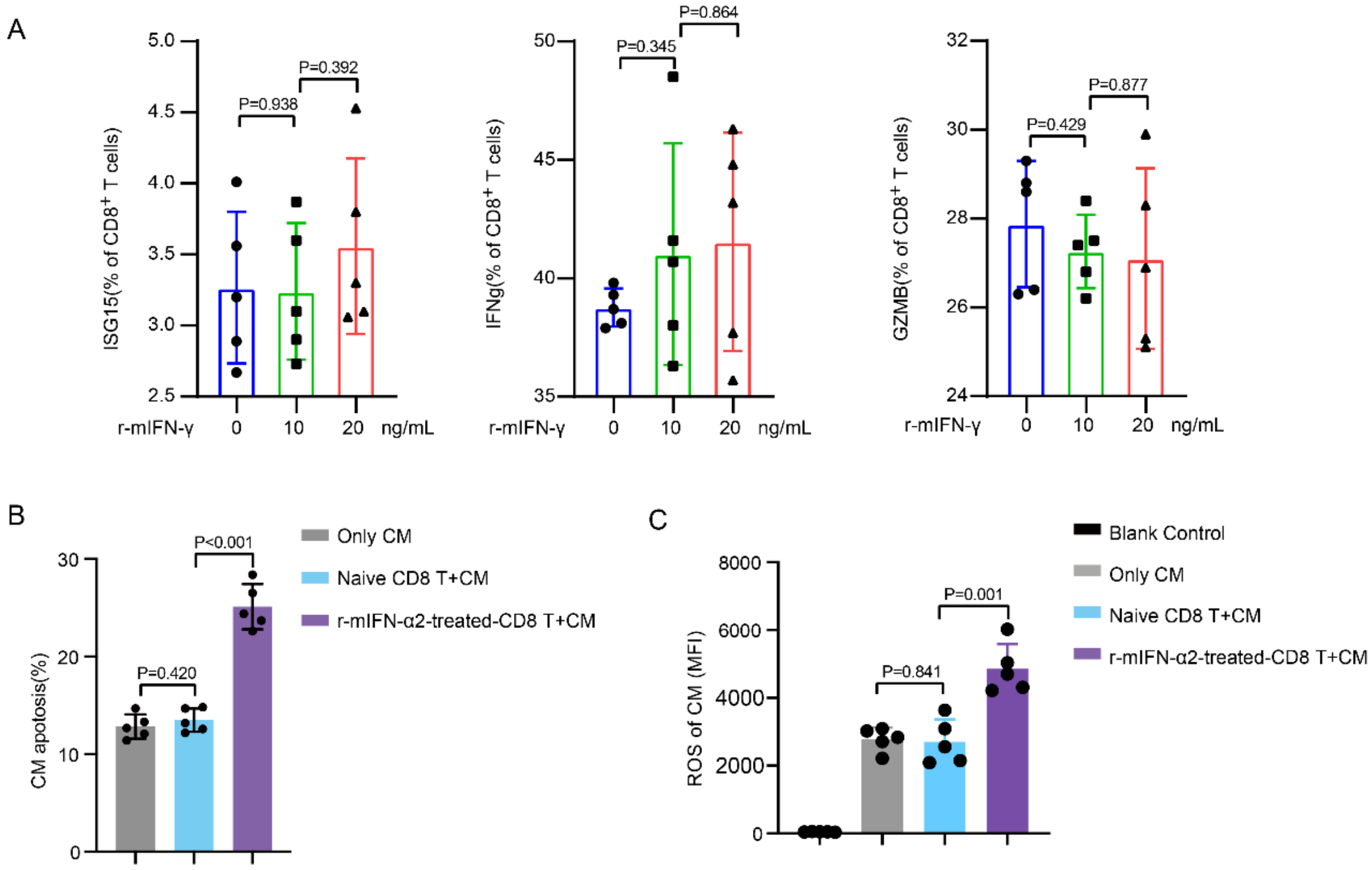

A, Flow cytometry analysis of ISG15, IFNG and GZMB in mouse CD8⁺ T cells following stimulation with recombinant mouse type II interferon (r-mIFN-γ) at the indicated concentrations (0, 10, and 20 ng/mL).

B, Quantification of cardiomyocyte apoptosis after coculture with ISG15⁺CD8⁺ T cells at a ratio of 1:10.

C, Quantitative analysis of reactive oxygen species (ROS) levels in cardiomyocytes co-cultured with ISG15⁺CD8⁺ T cells at a ratio of 1:10. The data are presented as means ± SD and analyzed by two-sided unpaired Student's t-tests. n = 5 biologically independent experiments.

Supplementary Fig. 7. CellChat analysis of intercellular communication among cell types in the in-house single-cell cohort.

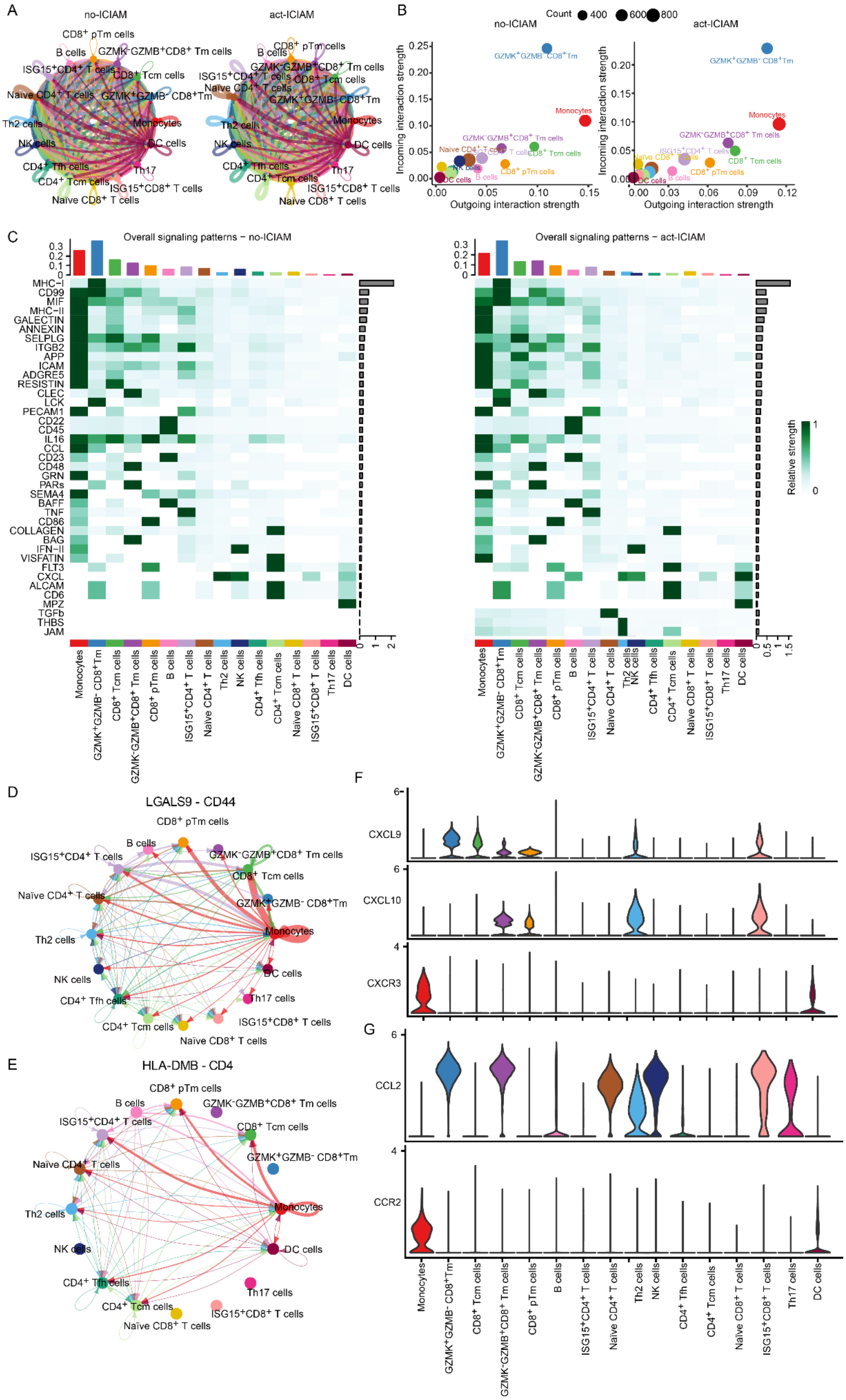

A, Number of intercellular interactions between the no-ICIAM and act-ICIAM groups.
B, Relationship between differential outgoing interactions and incoming interaction strength across cell subtypes in the no-ICIAM and act-ICIAM groups.
C, Overall signaling patterns of intercellular interactions between the no-ICIAM and act-ICIAM groups.
D, Circle plots showing significant LGALS9-CD44 ligand-receptor pair across immune cell subtypes in the no-ICIAM and ICIAM groups.
E, Circle plots showing the significant HLA-DMB-CD4 ligand-receptor pair across immune cell subtypes in the no-ICIAM and ICIAM groups.
F, Violin plots showing the expression of CXCL9-CXCR3, CXCL10-CXCR3 ligand-receptor pairs across cell subtypes.
G, Violin plot showing the expression of CCL2-CCR2 ligand-receptor pair across cell subtypes. ICI, immune checkpoint inhibitor; no-ICIAM, no ICI-myocarditis; act-ICIAM, active ICI-myocarditis.

Supplementary Fig. 8. Changes in inflammatory response and cytokines in the monocyte subclusters.

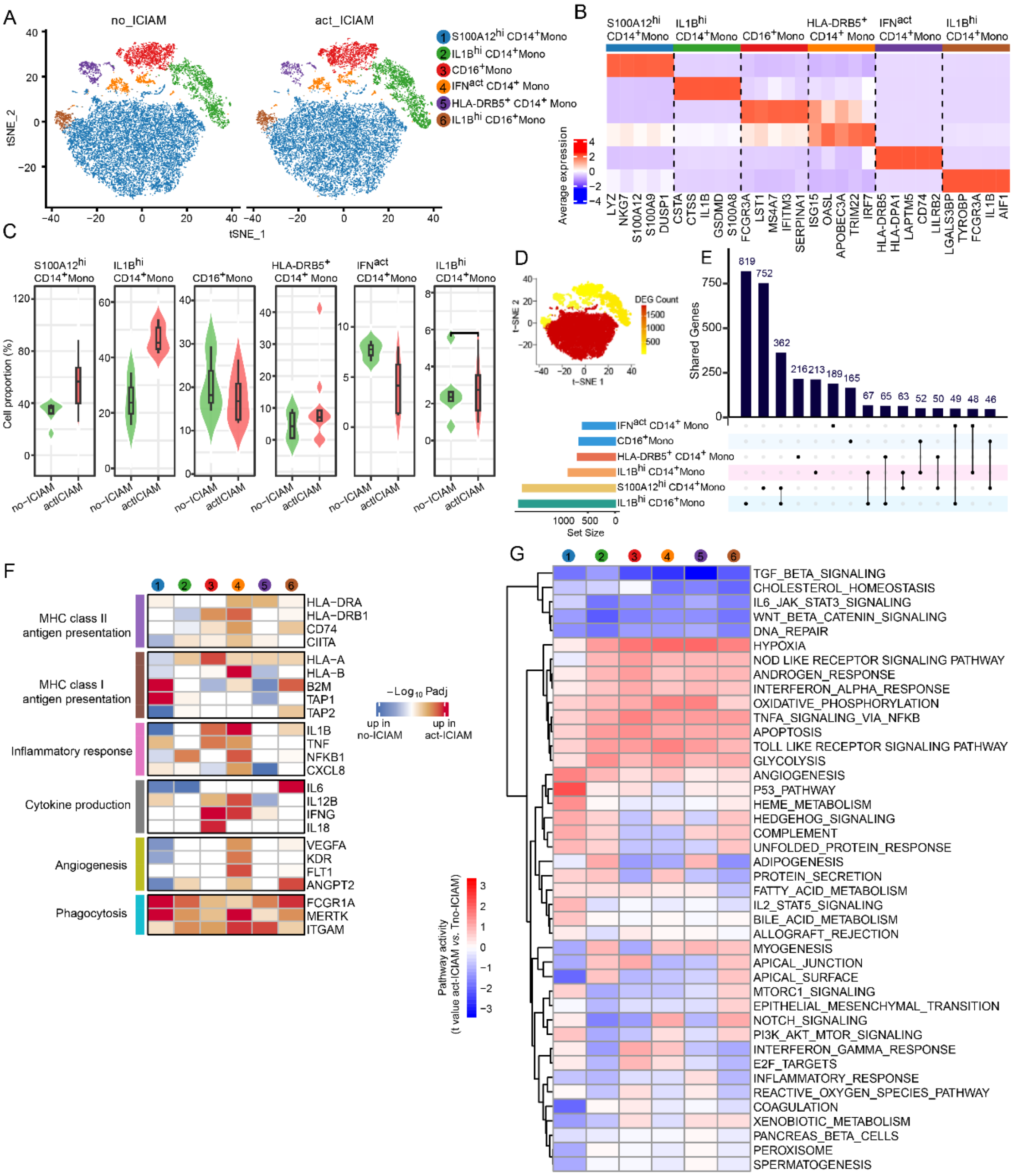

A, Identification of six subclusters of monocytes between the no-ICIAM and act-ICIAM groups.

B, Heatmap showing the scaled expression of top 5 DEGs among six subclusters of monocytes.

C, Violin and box plots showing the proportion of each monocyte subcluster between the no-ICIAM and act-ICIAM groups.

D, Feature plot showing the count of DEGs determined by a two-sided Wilcoxon rank-sum test.

E, UpSet plot showing all considered comparisons, with horizontal bars indicating the number of genes in each set and vertical bars showing the intersections, revealing shared DEGs across monocyte subsets.

F, Heatmap showing the relative change in the signature gene expression within each monocyte subcluster between the no-ICIAM and act-ICIAM groups.

G, Differences in pathway activities scored per cell in monocytes using GSVA, between the no-ICIAM and act-ICIAM groups. The t values are independent of effects from the patient of origin. ICI, immune checkpoint inhibitor; no-ICIAM, no ICI-myocarditis; act-ICIAM, active ICI-myocarditis; DEGs,differentially expressed genes; GSVA, gene set variation analysis.

Supplementary Fig. 9. Evaluation of candidate protein levels and diagnostic performance of machine learning models.

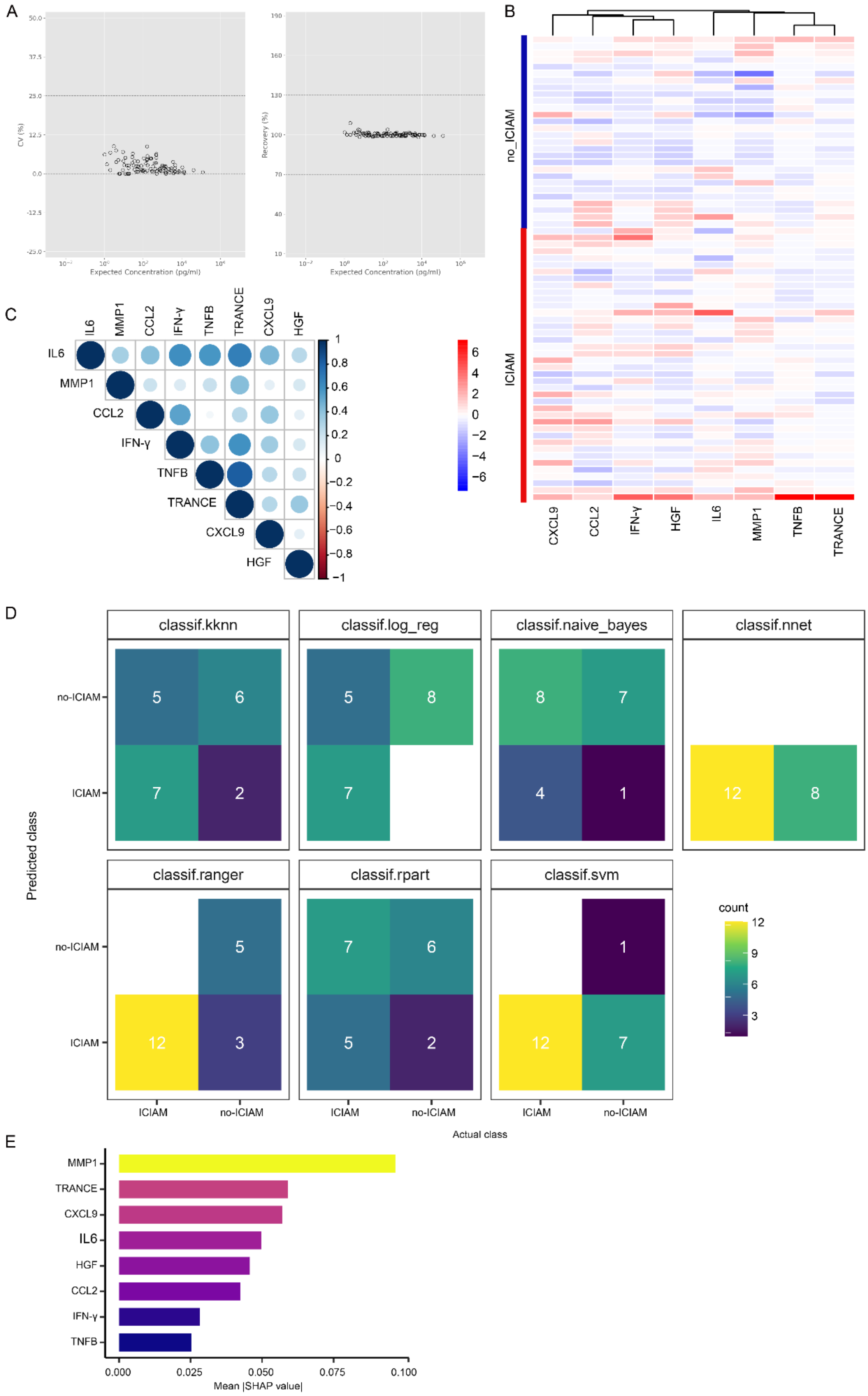

A, Quality control metrics of the custom-designed Luminex multiplex panel targeting eight candidate proteins.
B, Heatmap showing the relative expression levels of eight candidate proteins in patients with and without ICIAM, as measured by the Luminex panel.
C, Heatmap depicting pairwise Pearson correlations among the candidate proteins retained in the final panel.
D, Confusion matrices for each model, demonstrating classification accuracy, sensitivity, and specificity.
E, SHAP summary plot derived from the Random Forest model, illustrating the contribution of each feature to the predicted outcome. ICI, immune checkpoint inhibitor; ICIAM, ICI-myocarditis; SHAP, SHapley Additive exPlanations.

Supplementary Fig. 10. Bulk and single-cell transcriptomic validation of inflammatory signatures in the cardiac tissues.

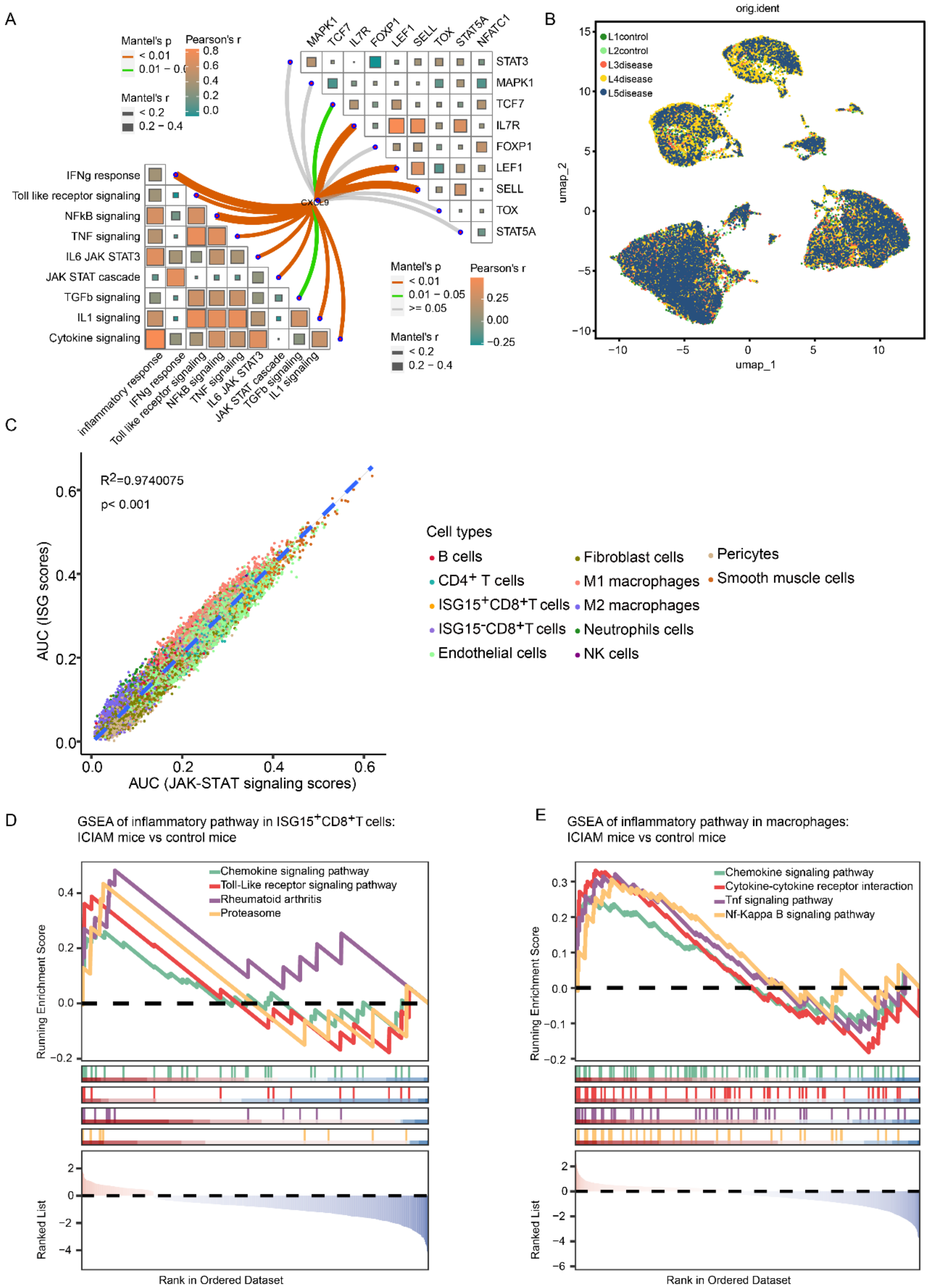

A, Mantel's correlations between CXCL9, T-cell exhaustion features, and inflammatory signals in the GSE57338 cohort (n = 313). Edge widths represented Mantel's r values, and edge colors indicated statistical significance levels. Pairwise correlation coefficients were shown as color gradients.

B, UMAP plots of cardiac tissues from each sample in the GSE227437 dataset (n = 5).
C, Correlation analysis between ISG scores and JAK-STAT signaling scores across cardiac cells from $Ctla4^{+}$/-Pdcd1-/- ICI-myocarditis mice in the GSE227437 dataset (n = 5). Spearman correlation analysis was applied.
D, GSEA of $ISG15^{+}CD8^{+}$ T cells for inflammatory pathways in ICI-myocarditis model mice compared with controls.
E, GSEA of macrophages for inflammatory pathways in ICI-myocarditis model mice compared with controls. ICI, immune checkpoint inhibitor; ICIAM, ICI-myocarditis; AUC, area under the curve; UMAP, uniform manifold approximation and projection.

Supplementary Fig. 11. CellChat analysis of intercellular communication across cell types in the GSE227437 dataset.

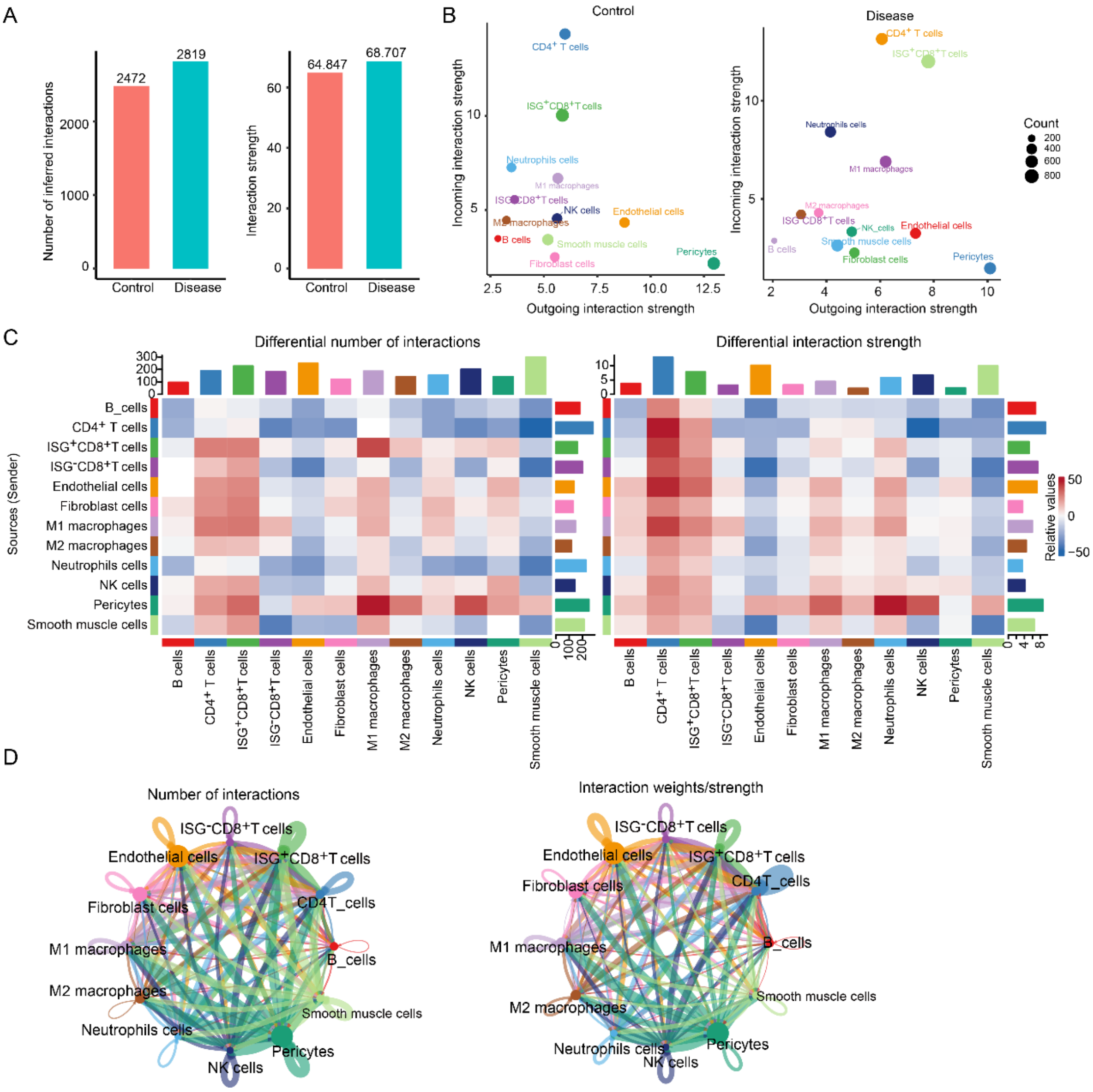

A, Total number and interaction strength of interactions between ICI-myocarditis mice and control mice.

B, Relationship between differential outgoing interactions and incoming interaction strengths across cardiac cell subtypes in ICI-myocarditis mice and control mice.

C, differential number and interaction strength between ICI-myocarditis mice and control mice.

D, Differences in the number and strength of communications across cardiac cell types between ICI-myocarditis mice and control mice.

Supplementary Tables

Supplementary Table 1. Baseline characteristics of recruited participants in the in-house scRNA-seq cohort (n = 19).

**Characteristic**	Overall N (%)	no-ICIAM N (%)	act-ICIAM N (%)	rem-ICIAM N (%)	P value
	19 (100)	6 (31.6)	10 (52.6)	3 (15.8)	
**Age**					0.463
≦ 65	8 (42.1)	4 (66.7)	3 (30.0)	1 (33.3)	
> 65	11 (57.9)	2 (33.3)	7 (70.0)	2 (66.7)	
**Sex**					0.124
Female	5 (26.3)	2 (33.3)	1 (10.0)	2 (66.7)	
Male	14 (73.7)	4 (66.7)	9 (90.0)	1 (33.3)	
**ECOG**					0.741
0	9 (47.4)	4 (66.7)	4 (40.0)	1 (33.3)	
1	7 (36.8)	2 (33.3)	4 (40.0)	1 (33.3)	
2	3 (15.8)	0 (0.0)	2 (20.0)	1 (33.3)	
**CTCAE**					<0.001
0	6 (31.6)	6 (100.0)	0 (0.0)	0 (0.0)	
1	1 (5.3)	0 (0.0)	1 (10.0)	0 (0.0)	
2	2 (10.5)	0 (0.0)	0 (0.0)	2 (66.7)	
3	9 (47.4)	0 (0.0)	8 (80.0)	1 (33.3)	
4	1 (5.3)	0 (0.0)	1 (10.0)	0 (0.0)	
**Diabetes mellitus**					0.700
No	10 (52.6)	4 (66.7)	5 (50.0)	1 (33.3)	
Yes	9 (47.4)	2 (33.3)	5 (50.0)	2 (66.7)	
**Tumor type**					0.990
Colorectal cancer	1 (5.3)	0 (0.0)	1 (10.0)	0 (0.0)	
Esophageal cancer	3 (15.8)	1 (16.7)	2 (20.0)	0 (0.0)	
Gastric cancer	1 (5.3)	0 (0.0)	1 (10.0)	0 (0.0)	
Hepatocellular carcinoma	3 (15.8)	1 (16.7)	1 (10.0)	1 (33.3)	
Lung cancer	5 (26.3)	2 (33.3)	2 (20.0)	1 (33.3)	
Breast cancer	1 (5.3)	0 (0.0)	1 (10.0)	0 (0.0)	
Cholangiocarcinoma	1 (5.3)	1 (16.7)	0 (0.0)	0 (0.0)	
Nasopharyngeal carcinoma	1 (5.3)	1 (16.7)	0 (0.0)	0 (0.0)	
Renal cell carcinoma	1 (5.3)	0 (0.0)	1 (10.0)	0 (0.0)	
Soft tissue sarcoma	1 (5.3)	0 (0.0)	1 (10.0)	0 (0.0)	
Thymic tumor	1 (5.3)	0 (0.0)	0 (0.0)	1 (33.3)	
**Tumor stage**					0.683
III	8 (42.1)	2 (33.3)	4 (40.0)	2 (66.7)	

IV	11 (57.9)	4 (66.7)	6 (60.0)	1 (33.3)	
**ICIs type**					0.703
Anti-PD-1	13 (68.4)	3 (50.0)	8 (80.0)	2 (66.7)	
Anti-PD-L1	4 (21.1)	2 (33.3)	1 (10.0)	1 (33.3)	
Anti-PD-1+ anti-CTLA-4	2 (10.5)	1 (16.7)	1 (10.0)	0 (0.0)	
**Treatment regimen**					0.918
ICI	2 (10.5)	1 (16.7)	1 (10.0)	0 (0.0)	
CT + ICI	10 (52.6)	2 (33.3)	6 (60.0)	2 (66.7)	
TT + ICI	4 (21.1)	2 (33.3)	1 (10.0)	1 (33.3)	
CT+ TT + ICI	3 (15.8)	1 (16.7)	2 (20.0)	0 (0.0)	

Abbreviations: ECOG, Eastern Cooperative Oncology Group; CTCAE, Common Terminology Criteria for Adverse Events; ICI, immune checkpoint inhibitor; PD-1, programmed cell death protein 1; PD-L1, programmed death-ligand 1; CTLA-4, cytotoxic T-lymphocyte-associated protein 4; CT, chemotherapy; TT, targeted therapy.

Supplementary Table 2. Baseline characteristics of recruited participants in the flow cytometry cohort (n = 57).

Characteristic	Total N (%)	no-ICIAM N (%)	act-ICIAM N (%)	rem-ICIAM N (%)	P value
	57 (100)	20 (35.1)	20 (35.1)	17 (29.8)	
**Age**					0.058
≦ 65	13 (22.8)	8 (40.0)	4 (20.0)	1 (5.9)	
＞ 65	44 (77.2)	12 (60.0)	16 (80.0)	16 (94.1)	
**Sex**					0.684
Male	43 (75.4)	15 (75.0)	14 (70.0)	14 (82.4)	
Female	14 (24.6)	5 (25.0)	6 (30.0)	3 (17.6)	
**ECOG**					0.937
0	25 (43.9)	8 (40.0)	10 (50.0)	7 (41.2)	
1	20 (35.1)	8 (40.0)	6 (30.0)	6 (35.3)	
2	10 (17.5)	4 (20.0)	3 (15.0)	3 (17.6)	
3	2 (3.5)	0 (0.0)	1 (5.0)	1 (5.9)	
**Hypertension**					0.835
No	34 (59.6)	12 (60.0)	11 (55.0)	11 (64.7)	
Yes	23 (40.4)	8 (40.0)	9 (45.0)	6 (35.3)	
**Diabetes mellitus**					0.535
No	49 (86.0)	16 (80.0)	17 (85.0)	16 (94.1)	
Yes	8 (14.0)	4 (20.0)	3 (15.0)	1 (5.9)	
**Coronary artery disease**					0.535
No	47 (82.5)	15 (75.0)	17 (85.0)	15 (88.2)	
Yes	10 (17.5)	5 (25.0)	3 (15.0)	2 (11.8)	
**Tumor type**					0.624
Colorectal cancer	7 (12.3)	2 (10.0)	3 (15.0)	2 (11.8)	
Esophageal cancer	7 (12.3)	1 (5.0)	4 (20.0)	2 (11.8)	
Gastric cancer	11 (19.3)	5 (25.0)	3 (15.0)	3 (17.6)	
Hepatocellular carcinoma	13 (22.8)	7 (35.0)	3 (15.0)	3 (17.6)	
Lung cancer	6 (10.5)	0 (0.0)	3 (15.0)	3 (17.6)	
Others	13 (22.8)	5 (25.0)	4 (20.0)	4 (23.5)	
**Tumor stage**					0.904
III	25 (43.9)	9 (45.0)	8 (40.0)	8 (47.1)	
IV	32 (56.1)	11 (55.0)	12 (60.0)	9 (52.9)	
**ICIs type**					0.831
Anti-PD-1	41 (71.9)	15 (75.0)	13 (65.0)	13 (76.5)	
Anti-PD-L1	10 (17.5)	4 (20.0)	4 (20.0)	2 (11.8)	
Anti-PD-1 + anti-CTLA-4	6 (10.5)	1 (5.0)	3 (15.0)	2 (11.8)	
**Treatment regimen**					0.563

ICI	10 (17.5)	1 (5.0)	5 (25.0)	4 (23.5)
CT + ICI	25 (43.9)	10 (50.0)	8 (40.0)	7 (41.2)
TT + ICI	17 (29.8)	8 (40.0)	5 (25.0)	4 (23.5)
CT + TT + ICI	5 (8.8)	1 (5.0)	2 (10.0)	2 (11.8)

Abbreviations: ECOG, Eastern Cooperative Oncology Group; ICI, immune checkpoint inhibitor; PD-1, programmed cell death protein 1; PD-L1, programmed death-ligand 1; CTLA-4, cytotoxic T-lymphocyte-associated protein 4; CT, chemotherapy; TT, targeted therapy.

Supplementary Table 3. Baseline characteristics of recruited participants in the Luminex assay cohort (n = 68).

Characteristic	Total N (%)	no-ICIAM N (%)	act-ICIAM N (%)	P value
	68 (100%)	28 (41.2%)	40 (58.8%)	
**Age**				0.263
≦65	37 (54.4)	18 (64.3)	19 (47.5)	
> 65	31 (45.6)	10 (35.7)	21 (52.5)	
**Sex**				0.844
Male	44 (64.7)	19 (67.9)	25 (62.5)	
Female	24 (35.3)	9 (32.1)	15 (37.5)	
**ECOG**				0.240
0	26 (38.2)	8 (28.6)	18 (45.0)	
1	30 (44.1)	13 (46.4)	17 (42.5)	
2	11 (16.2)	7 (25.0)	4 (10.0)	
3	1 (1.5)	0 (0.0)	1 (2.5)	
**Hypertension**				1.000
No	42 (61.8)	17 (60.7)	25 (62.5)	
Yes	26 (38.2)	11 (39.3)	15 (37.5)	
**Diabetes mellitus**				0.965
No	33 (48.5)	13 (46.4)	20 (50.0)	
Yes	35 (51.5)	15 (53.6)	20 (50.0)	
**Coronary artery disease**				1.000
No	62 (91.2)	26 (92.9)	36 (90.0)	
Yes	6 (8.8)	2 (7.1)	4 (10.0)	
**Tumor type**				0.151
Colorectal cancer	4 (5.9)	0 (0.0)	4 (10.0)	
Esophageal cancer	8 (11.8)	1 (3.6)	7 (17.5)	
Gastric cancer	6 (8.8)	3 (10.7)	3 (7.5)	
Hepatocellular carcinoma	15 (22.1)	9 (32.1)	6 (15.0)	
Lung cancer	15 (22.1)	7 (25.0)	8 (20.0)	
Others	20 (29.4)	8 (28.6)	12 (30.0)	
**Tumor stage**				0.216
II	2 (2.9)	2 (7.1)	0 (0.0)	
III	22 (32.4)	8 (28.6)	14 (35.0)	
IV	44 (64.7)	18 (64.3)	26 (65.0)	
**ICIs type**				0.483
Anti-PD-1	61 (89.7)	26 (92.9)	35 (87.5)	
Anti-PD-L1	5 (7.4)	2 (7.1)	3 (7.5)	
Anti-PD-1 + anti-CTLA-4	2 (2.9)	0 (0.0)	2 (5.0)	
**Treatment regimen**				0.878
ICI	21 (30.9)	10 (35.7)	11 (27.5)	
CT + ICI	33 (48.5)	13 (46.4)	20 (50.0)	

TT + ICI	7 (10.3)	3 (10.7)	4 (10.0)
CT + TT + ICI	7 (10.3)	2 (7.1)	5 (12.5)

Abbreviations: ECOG, Eastern Cooperative Oncology Group; ICI, immune checkpoint inhibitor; PD-1, programmed cell death protein 1; PD-L1, programmed death-ligand 1; CTLA-4, cytotoxic T-lymphocyte-associated protein 4; CT, chemotherapy; TT, targeted therapy.

Supplementary Table 4. Marker genes used to annotate peripheral blood mononuclear cell (PBMC) clusters identified by scRNA-seq.

**Main Cell Types**	**Cell Subtypes**	**Reference Markers**
NK cells		KLRD1/GNLY/NKG7/CD160
Monocytes		CD68/LYZ/CD14
B cells		CD79A/CD19
$CD4^+$T cells	$CD4^+$ T lineage	CD3D/CD4
$CD4^+$T cells	Naïve $CD4^+$ T cells	SELL/CCR7/LEF1
$CD4^+$T cells	$ISG15^+CD4^+$ T cells	ISG15/IFIT3/IFITM1
$CD4^+$T cells	$CD4^+$ Tcm cells	GPR183/MYADM/DUSP1
$CD4^+$T cells	$CD4^+$ Tfh cells	CXCR5/DUSP5/KLRB1
$CD4^+$T cells	Th17 cells	CCR6/KLRB1/LGALS3
$CD4^+$T cells	Th2 cells	CXCR4/ITGB1/LGALS1
$CD8^+$T cells	$CD8^+$ T lineage	CD3D/CD8
$CD8^+$T cells	Naïve $CD8^+$ T cells	SELL/CCR7/LEF1
$CD8^+$T cells	$CD8^+$ Tcm	GPR183/IL7R/LTB
$CD8^+$T cells	$GZMK^+GZMB^-$ $CD8^+$Tm	GZMA/GZMK/CMC1
$CD8^+$T cells	$ISG15^+$ $CD8^+$ T cells	ISG15/IFIT3/IFITM1
$CD8^+$T cells	$GZMK^-GZMB^+CD8^+$ Tm cells	GZMA/GZMB/FGFBP2
$CD8^+$T cells	$CD8^+$ pTm cells	MKI67/TOP2A/STMN1
