# Supplementary material for: Integrated multi-omics identifies distinct immune signatures with diagnostic and therapeutic implications in immune checkpoint inhibitor-induced myocarditis
Source: Nat Commun. 2026 Oct 14;17:11591. doi: 10.1038/s41467-026-77262-0 (PMC13751616; doi:10.1038/s41467-026-77262-0)
Supplement: Supplementary file 2 [file 41467_2026_77262_MOESM2_ESM.pdf]

nature portfolio

Corresponding author(s): Yan Wang

Last updated by author(s): Nov 24, 2025

Reporting Summary

Nature Portfolio wishes to improve the reproducibility of the work that we publish. This form provides structure for consistency and transparency in reporting. For further information on Nature Portfolio policies, see our Editorial Policies and the Editorial Policy Checklist.

Statistics

For all statistical analyses, confirm that the following items are present in the figure legend, table legend, main text, or Methods section.

n/a	Confirmed	
☐	☒	The exact sample size (*n*) for each experimental group/condition, given as a discrete number and unit of measurement
☐	☒	A statement on whether measurements were taken from distinct samples or whether the same sample was measured repeatedly
☐	☒	The statistical test(s) used AND whether they are one- or two-sided<br>*Only common tests should be described solely by name; describe more complex techniques in the Methods section.*
☒	☐	A description of all covariates tested
☒	☐	A description of any assumptions or corrections, such as tests of normality and adjustment for multiple comparisons
☐	☒	A full description of the statistical parameters including central tendency (e.g. means) or other basic estimates (e.g. regression coefficient) AND variation (e.g. standard deviation) or associated estimates of uncertainty (e.g. confidence intervals)
☐	☒	For null hypothesis testing, the test statistic (e.g. *F*, *t*, *r*) with confidence intervals, effect sizes, degrees of freedom and *P* value noted<br>*Give P values as exact values whenever suitable.*
☒	☐	For Bayesian analysis, information on the choice of priors and Markov chain Monte Carlo settings
☒	☐	For hierarchical and complex designs, identification of the appropriate level for tests and full reporting of outcomes
☐	☒	Estimates of effect sizes (e.g. Cohen's *d*, Pearson's *r*), indicating how they were calculated

Our web collection on statistics for biologists contains articles on many of the points above.

Software and code

Policy information about availability of computer code

Data collection	For PBMC samples, single-cell suspensions were loaded onto a Chromium Single Cell Controller (10x Genomics) to generate Gel Bead-In-Emulsions (GEMs). Single-cell 3' gene expression libraries were prepared by strictly following the protocols of a Chromium Single Cell 3'v2 Library kit (10x Genomics). All resulting libraries were sequenced on the Illumina NovaSeq 6000 PE150 platform.
Data analysis	CellRanger v7.1.0;Seurat v4.0.1; DoubletFinder v2.0.3; Harmony v2.0.5;circlize v0.4.15; monocle v2.22.0; scran v1.2.0; clusterProfiler v3.19.0; AUCell v1.14.0; GSVA v1.50.5; CellChat v1.6.1; psych v2.3.3; corrplot v0.92; nichenetr v1.1.0; batchelor v1.16.0; pheatmap v1.0.12; ggplot2 v3.4.2; ggsci v3.0.0; bseqsc v1.0; FlowJo software v10.0.7;R software v4.2.0; GraphPad Prism v8.0.1

For manuscripts utilizing custom algorithms or software that are central to the research but not yet described in published literature, software must be made available to editors and reviewers. We strongly encourage code deposition in a community repository (e.g. GitHub). See the Nature Portfolio guidelines for submitting code & software for further information.

Data

Policy information about availability of data

All manuscripts must include a data availability statement. This statement should provide the following information, where applicable:

- Accession codes, unique identifiers, or web links for publicly available datasets
- A description of any restrictions on data availability
- For clinical datasets or third party data, please ensure that the statement adheres to our policy

All relevant data related to this manuscript are available within the Article, Supplementary Information or Source Data file. Raw sequencing data of single-cell RNA

data generated in this study have been deposited in the National Genomics Data Center with the accession code HRA019659. This research reanalyzes existing, publicly available genomic datasets, including data from the Gene Expression Omnibus (GEO) database under accession number (GSE228597, GSE227437, and GSE57338).

Research involving human participants, their data, or biological material

Policy information about studies with human participants or human data. See also policy information about sex, gender (identity/presentation), and sexual orientation and race, ethnicity and racism.

Reporting on sex and gender	This study doesn't report any data or results related to sex and gender.
Reporting on race, ethnicity, or other socially relevant groupings	The blood samples used in this study were obtained exclusively from Chinese individuals, including both Asian maleand Asian female. All samples were collected from Zhongshan Hospital affiliated to Fudan University, located at Shanghai, China. The race, ethnicity, or other socially relevant groupings were not used as covariates in this study.
Population characteristics	See Above.
Recruitment	We consecutively enrolled adult cancer patients who received anti-PD-1, anti-PD-L1, anti-CTLA-4, or combination ICI therapies at Zhongshan Hospital, Fudan University, between September, 30 2017 and December, 31 2024, and identified patients diagnosed with ICI-related myocarditis. Adult (age >18 years) patients who were treated with an ICI agent were identified from the Zhongshan Hospital institutional patient's records. Charts were reviewed for a clinical diagnosis of ICI-related myocarditis, defined according to the criteria of Bonaca et al.The first and senior authors undertook a second round of review to adjudicate all patients identified as having myocarditis to ensure adherence with the defining criteria,Supplemental Table 1, 2 and 3. The Bonaca definition was applied as this was the main diagnostic definition used up to the census date of the study. Although it uses similar variables to the subsequent International Cardio-Oncology Society definition, it differs in that it does not require a troponin elevation for diagnosis (and so may capture cases after the troponin rise if they are manifested subacutely) and has sub-categories.
Ethics oversight	This study was approved by the Institutional Review Board of Zhongshan Hospital, Fudan University(B2022-444). Written informed consent was obtained from all participants. All procedures adhered to the ethical principles outlined in the Declaration of Helsinki.

Note that full information on the approval of the study protocol must also be provided in the manuscript.

Field-specific reporting

Please select the one below that is the best fit for your research. If you are not sure, read the appropriate sections before making your selection.

[x] Life sciences [ ] Behavioural & social sciences [ ] Ecological, evolutionary & environmental sciences

For a reference copy of the document with all sections, see nature.com/documents/nr-reporting-summary-flat.pdf

Life sciences study design

All studies must disclose on these points even when the disclosure is negative.

Sample size	No standard methods were used to predetermine sample size. The sample sizes are sufficient to provide stable single cell clustering results and to perform statistical analysis
Data exclusions	For scRNA-Seq data, We excluded low quality cells through quality control pipeline, and cell types with less than 200 unique genes, more than 4000 unique genes, and those with more than 10% mitochondrial genes.
Replication	Findings derived from scRNA-seq analysis were validated by flow cytometry study performed in independent groups of ICIAM patients and all attempts were successful.
Randomization	No randomization was performed due to the nature of the study, as all patients were diagnosed with inflammatory myositis and treated by standard procedure.
Blinding	For scRNA-seq, flow cytometry and Olink proteomics data, the experimenters were blinded to the sample information. For analysis, the initial analysis for individual samples were also blinded to the analyzers; then for the group analysis, the sample information was notified to the analyzers, as the purpose was to identify the difference between groups.

Reporting for specific materials, systems and methods

We require information from authors about some types of materials, experimental systems and methods used in many studies. Here, indicate whether each material, system or method listed is relevant to your study. If you are not sure if a list item applies to your research, read the appropriate section before selecting a response.

Materials & experimental systems

n/a	Involved in the study	
[ ]	[x]	Antibodies
[x]	[ ]	Eukaryotic cell lines
[x]	[ ]	Palaeontology and archaeology
[ ]	[x]	Animals and other organisms
[ ]	[x]	Clinical data
[x]	[ ]	Dual use research of concern
[x]	[ ]	Plants

Methods

n/a	Involved in the study	
[x]	[ ]	ChIP-seq
[ ]	[x]	Flow cytometry
[x]	[ ]	MRI-based neuroimaging

Antibodies

Antibodies used	APC anti-human CD8 (BD Pharmingen, Clone: HIT8A, #566852), APC-Cy7 anti-human CD3 (BD Pharmingen, 557757), FITC anti-human CD4 (BD Pharmingen, 555346), and PE-conjugated anti-human ISG15/UCRP antibody (R&D Systems, IC8044P)
Validation	All antibodies were obtained commercially validated by the respective company. All antibodies had validation statement provided on the website of the manufacturer. Related technical data sheets can be obtained from the manufacturer's website using the catalog number provided above.

Animals and other research organisms

Policy information about studies involving animals; ARRIVE guidelines recommended for reporting animal research, and Sex and Gender in Research

Laboratory animals	Female, mice, C57BL/6, 6-8 weeks old.
Wild animals	This study doesn't involve wild animals.
Reporting on sex	This study doesn't report any data or results related to sex and gender.
Field-collected samples	Mice were housed under a 12-hour light/dark cycle with food and drinking water provided ad libitum. Room temperature was maintained within the range of 22-24 °C and relative humidity ranged between 45-55%. All mice were euthanized by cervical dislocation at the end of experiment.
Ethics oversight	All animal care and protocols conducted according to guidelines of Institutional Laboratory Animal Care and Use Committee of the Zhongshan Hospital, Fudan University.

Note that full information on the approval of the study protocol must also be provided in the manuscript.

Clinical data

Policy information about clinical studies

All manuscripts should comply with the ICMJE guidelines for publication of clinical research and a completed CONSORT checklist must be included with all submissions.

Clinical trial registration	This is not a clinical trial.
Study protocol	Study design and recruitment of participants can be found in Methods section. Briefly, we prospectively recruited patients with newly developed ICI-related myocarditis after immune checkpoint inhibitor administration and rheumatoid arthritis. FPeripheral blood samples were collected from the patients through venepuncture. Mononuclear cells were isolated from peripheral blood samples and were further used for sequencing and flow cytometry. Supernatant serum were used for ELISA tests.
Data collection	Clinical information were collected from medical records.
Outcomes	Not applicable.

Plants

Seed stocks: NA

Novel plant genotypes: NA

Authentication: NA

Flow Cytometry

Plots

Confirm that:

- [x] The axis labels state the marker and fluorochrome used (e.g. CD4-FITC).
- [x] The axis scales are clearly visible. Include numbers along axes only for bottom left plot of group (a 'group' is an analysis of identical markers).
- [x] All plots are contour plots with outliers or pseudocolor plots.
- [x] A numerical value for number of cells or percentage (with statistics) is provided.

Methodology

Sample preparation: PBMCs were initially incubated with Zombie Yellow (Biolegend, Cat: 423104) to discriminate and remove nonviable cells. For surface labeling, the cells were stained with fluorochrome-conjugated antibodies in a staining buffer (consisting of PBS, 5% FBS, 2 mM EDTA, and 0.09% NaN3) for 15 minutes at room temperature. Subsequently, cells were fixed and permeabilized using the Cytofix/Cytoperm Fixation/Permeabilization Kit (BD, Cat: 554714), then stained intracellularly with the relevant fluorophore-tagged antibodies in permeabilization buffer for 30 minutes at 4°C.

Instrument: Data acquisition was performed on a BD LSRFortessa flow cytometer.

Software: FlowJo software (v10.0.7)

Cell population abundance: The purity was verified by flow cytometry. Frequencies of each cell population are displayed in all flow cytometry dot plots in main and supplementary Figures. Raw data are available in the source data file.

Gating strategy: The gating strategies are shown in Supplementary Fig. 3E.

- [x] Tick this box to confirm that a figure exemplifying the gating strategy is provided in the Supplementary Information.
