# Supplementary material for: Integrated multi-omics identifies distinct immune signatures with diagnostic and therapeutic implications in immune checkpoint inhibitor-induced myocarditis
Source: Nat Commun. 2026 Oct 14;17:11591. doi: 10.1038/s41467-026-77262-0 (PMC13751616; doi:10.1038/s41467-026-77262-0)
Supplement: Supplementary file 3 [file 41467_2026_77262_MOESM3_ESM.pdf]

nature portfolio

Peer Review File

Integrated multi-omics identifies distinct immune signatures with diagnostic and therapeutic implications in immune checkpoint inhibitor-induced myocarditis

Corresponding Author: Professor Shi-Long Zhang

This file contains all reviewer reports in order by version, followed by all author rebuttals in order by version.

A version of this paper was originally rejected for publication by Nature Communications, however that decision was reconsidered after appeal by the authors.

Version 0:

Reviewer comments:

Reviewer #1

(Remarks to the Author)
In this study, Shi-Long Zhang et al. characterize the cellular and molecular landscape of the rare but often lethal immune checkpoint inhibitor–associated myocarditis (ICIAM) in a large prospective cohort, screening nearly 20,000 patients for this uncommon complication. The authors collected peripheral blood mononuclear cells (PBMCs) and serum samples from patients during acute ICIAM, after ICIAM remission, and from ICI-treated patients who did not develop myocarditis, enabling a comprehensive analysis of the immune land-scape associated with this adverse event.

The study reveals pronounced alterations in the immune landscape during acute ICIAM, including changes in the frequencies of multiple $CD8^+$ T-cell subsets. Notably, the authors identify a distinct population of $ISG15^+$ $CD8^+$ T cells. They further demonstrate marked upregulation of the JAK–STAT signaling pathway in acute ICIAM and suggest that immune cell crosstalk is partially mediated through the CCR2–CCL2 axis. Using an in vivo mouse model, the authors show that blockade of the CCR2–CCL2 axis or inhibition of JAK–STAT signaling reduces $CD8^+$ T-cell infiltration and improves cardiac function, respectively. In addi-tion, they develop an eight-protein biomarker panel capable of distinguishing acute ICIAM samples from other ICI-treated patients and validate this panel in an independent cohort.

The management of immune-related adverse events represents a major challenge in clinical oncology and a key focus of translational oncoimmunology research. Overall, this study addresses an important and timely topic by defining immune cell profiles and intercellular communication pathways involved in acute ICIAM, while highlighting potential therapeutic targets for improved disease management. However, some sections of the manuscript are dense and repetitive. The following comments are intended to help improve the overall clarity, readability, and quality of the manuscript.

Major Issues
1. Contextualization of ISG15 biology
ISG15 is, by definition, an interferon-stimulated gene. However, this well-established biological con-text is not discussed in the manuscript, and the upregulation of JAK–STAT signaling within the $ISG15^+$ $CD8^+$ T-cell subset is presented as somewhat unexpected. Including a brief overview of what is currently known in the literature about $ISG15^+$ $CD8^+$ T cells and their association with inter-feron signaling would provide important context and help readers better interpret these findings.
2. Rationale for focusing on $ISG15^+$ $CD8^+$ T cells
The manuscript convincingly shows a marked increase in the proportion of $ISG15^+$ $CD8^+$ T cells in acute ICIAM compared with non-ICIAM samples, suggesting that this subset may be biologically rel-evant. However, other immune cell populations also display significant frequency shifts but are not further explored. Providing a clearer rationale for prioritizing this specific subset, and commenting on whether similar analyses were performed (or considered) for other altered populations, would strengthen the interpretation of the results.
3. Specificity of the 8-protein panel
The proposed 8-protein panel includes broadly acting immunomodulatory molecules (e.g., IFNγ, IL-6, and chemokines). While the panel effectively distinguishes acute ICIAM samples from other ICI-treated samples, it remains unclear whether it

is specific for ICIAM as opposed to other immune-related adverse events (irAEs) or cardiac conditions. Testing the panel in plasma samples from pa-tients with other irAEs, if available, would help clarify its diagnostic specificity.

4. Clinical applicability of the protein panel

The authors clearly outline the diagnostic challenges of ICIAM, including the need for multiple diag-nostic tools and exclusion of alternative causes, which may delay treatment and lead to myocardial damage. In this context, the development of the 8-protein panel is highly relevant. Expanding the discussion to address how this panel could be implemented in clinical practice—particularly with re-spect to feasibility, cost, turnaround time, and how it compares to current diagnostic standards—would further highlight its potential clinical impact.

5. Causal role of ISG15⁺ CD8⁺ T cells

At present, the evidence provided does not fully support the conclusion that ISG15⁺ CD8⁺ T cells play a detrimental role in disease pathogenesis (line 336). The mouse experiments involve depletion of all peripheral CD8⁺ T cells prior to myocarditis induction. While these data are informative, they do not specifically address the contribution of the ISG15⁺ subset, which represents only a fraction of the total CD8⁺ T-cell pool. This distinction should be clarified, and conclusions moderated accordingly.

6. Comparison with recent literature (Blum et al., Nature 2024)

A recent study by Blum et al. (Nature, 2024) analyzed blood and heart tissue from patients with im-mune-related myocarditis and identified cytotoxic CD8⁺ T cells as key contributors to disease. This population is not discussed in the current manuscript, and some discrepancies between the blood-based findings of the two studies are apparent. Addressing these differences, proposing possible ex-planations, and—if feasible—validating key findings using this external dataset would substantially enhance the robustness and impact of the study.

7. Link between blood-based findings and cardiac tissue pathology

The primary analyses are based on blood and serum samples collected during acute ICIAM. While significant immune alterations are identified, the mechanistic connection between circulating immune changes and myocardial tissue damage remains unclear. If available, additional mechanistic insights could be included; alternatively, this limitation could be explicitly acknowledged in the Discussion.

8. Validation of ISG15⁺ CD8⁺ T cells in tissue datasets

Some findings are validated using bulk mRNA sequencing of human myocardial tissue and single-cell RNA sequencing of mouse myocardial tissue. However, given that a central conclusion of the study is the pathogenic role of ISG15⁺ CD8⁺ T cells, stronger validation in myocardial tissue would be valuable. Specifically, it would be helpful to clarify whether this population is detectable in the val-idation datasets or in other single-cell datasets derived from ICIAM myocardial tissue (e.g., Blum et al., 2024), rather than normal heart tissue.

9. Interpretation of JAK–STAT pathway inhibition

The JAK–STAT pathway is widely recognized for its roles in cell survival, immune regulation, and, in some contexts, immune escape mechanisms (e.g., PD-L1 upregulation in tumors). Given these known functions, further discussion of the potential risks, limitations, and broader implications of in-hibiting this pathway—particularly in cancer patients—would be important.

10. Steroid treatment status

Please clarify whether patients with active ICIAM were receiving corticosteroid treatment at the time of sample collection, as this could significantly influence immune profiles and should be accounted for in the interpretation of the data.

11. Clarifications and interpretative questions

o Line 391: Please specify whether this refers to the discovery or validation cohort.
o Line 431: "Optimal predictive model" should be defined more clearly—optimal compared to which alternatives?
o While interferon signaling and T-cell infiltration are associated with inflammation, the mecha-nistic link to ICIAM development remains unclear. Are ISG15⁺ CD8⁺ T cells driving myocar-ditis, or does an inflamed myocardium promote IFNγ production and secondary ISG15 ex-pression in infiltrating T cells? Clarifying/discussing this point would improve mechanistic un-derstanding.

Minor Issues

- Marker genes identified by DEG analysis (FindAllMarkers) for each T-cell cluster should be provid-ed.
- The repeated listing of techniques (scRNA-seq, flow cytometry, Olink, multiplex cytokine assays; e.g., lines 181 and 208) could be streamlined by introducing methods only when their corresponding data are first presented.
- t-SNE plots are shown in the figures but not described in the Methods section.
- The section beginning at line 301 contains results that do not fully align with the section title; subdivid-ing the section based on the underlying rationale may improve clarity.
- Figure 1E: The figure description does not match the text.
- Figures 2A and 2G: Please provide dot plots showing DEG expression by cluster.
- A reference to Figure 2E is missing in the text.
- The readability of the text in Figure 3A could be improved.
- Figure 3H: Clusters are labeled as 0–3 in the legend but elsewhere as C1–C4; consistent labeling is recommended. Alternatively, the legend could be simplified.
- Line 381: Please clearly indicate that the following section refers to the mouse model.
- Figure 6A: The legend colors for "group" do not match those used in the figure.
- Figure 7C: Increasing color contrast would improve discrimination between overlapping cell types (e.g., endothelial cells and M1 macrophages).
- Figure 7E: Label readability could be improved.
- Figure 7F: A legend description is missing.
- Several typographical errors are present (e.g., line 176 missing parenthesis; line 204 "analyzed" in-stead of "analyzing"; line 504).
- (Optional) Please comment on whether blockade of the CCL2–CCR2 axis affects tumor growth.

• QC preprocessing cutoffs are repeated (lines 699 and 709).
• Olink is a well-established technique and does not require a detailed methodological explanation.
• Line 805: Please provide a table listing the antibodies used.

Reviewer #2

(Remarks to the Author)
Overall, Zhang et al. provide interesting data on ICIAM by employing single-cell omic methods as well as circulating plasma proteomics. They confirm key findings by reverse-translational approaches to the mouse model, showing that the CCL2-CCR2 axis recruits distinct, pathogenic ISG15+ CD8+ T-cell subsets which associate with adverse outcomes. The depletion of either rescues disease severity. Interestingly, inhibiton of cardiac toxicity of PD1 inhibitors by targetting culprit JAK/Stat signalling simultaneously boosters anti-tumor activity of PD1 inhibition.
This is an interesting study, yet requires major further improvements as it – as it currently stands – is rather preliminary yet.
1. Statistics in Figure 1 includes too few patients with too high variability, a higher number of patients should be included at least for a FACS based validation in an independent validation cohort, focussing on ISG15+ CD8+ T-cells
2. Would it be possible that the corticosteroids / treatment of ICI affect the resonses
3. Figure 2 does not employ proper statistics per patient but regards all cells from a conditiion as individual replicates, this should be changed
4. The concept of a exploratory and validation cohort is not correct. Comparing cell-specific expression levels with circulating cytokines is far-fetched. The validation cohort is proteomics based and the exploratory cohort is cell based. This should be rephrased.
5. Language should be improved throughout the complete manuscript
6. The claim that depleting CD8+ T-cells in total corresponds to the depletion of ISG15+ CD8+ T-cells is too far fetched. Different CD8+ T-cell subsets might have fully opposite roles, hence depleting CD8+ T-cells in total is solely an approximation
7. In Figure 6A the legend does not match the colors in the Figure (i.e. group)
8. Figure 6J should be better explained, this remains
9. It remains unclear what is meant by L1-L5 in Figure7D (what are the different disease stages mentioned in the x-axis?
10. It does not become clear how „exhaustion“ is distinctly defined

Reviewer #3

(Remarks to the Author)
The manuscript of Zhang et al. addresses immune response during immune checkpoint inhibitor-associated myocarditis using scRNAseq on PBMC and plasma Olink/Luminex in human cohort with support of experiments in mouse model. Authors point to $ISG15^+CD8^+$ T cells as a key players in this condition, JAK-STAT pathway activation and CCR2-CCL2 axis as critical molecular mechanism. Furthermore, authors developed a 8-protein classifiers to identify ICIAM.

Overall, the manuscript is overloaded with bioinformatics analyses based on rather limited biological samples. Based of their nature, these analyses serve as hypothesis-generating tools. The actual validation of these hypotheses is mostly poor or inadequate. Furthermore, the experimental part often does not follow GLP principles.

Specific comments:
1) Clinical cohorts. Discovery cohort show no comparison between analysed groups (no-ICIAM, act-ICIAM, and rem-ICIAM) for clinical parameters. Validation cohort is impressive, but no experiment is performed on the whole cohort! For example, flow cytometry analysis or ELISAs show n=22 (out of 270). It is unclear how patients were preselected.

2) Mouse studies. Presented data show no myocarditis in this model, which is not surprising as BL6 mice are myocarditis-resistant. CD45 looks unspecific, with no inflammatory aggregates typical for myocarditis (on H/E or CD45 IHC). It is very concerning that experimental data for mouse studies vary between n=3-5. For example, in Fig. 8 in anti-PD1 PBS group for serum measurement n=5, while for EF n=3. Furthermore, Method section describes (briefly) only 1 out of 3 mouse experiments.

3) Validation in mouse study is superficial. Even if experiments were performed in the right model, blockade of all CD8+ cells can not validate pathogenic role of ISG15+CD8+ T subset as suggested in this manuscript.

4) Along the same line, analysis of transient appearance of ISG15+CD8+ T cells can be directly measured in the blood of the same patients during and post myocarditis. Instead, authors used monocle pseudotime trajectory analysis with validation of total CD8+ in mouse model which is superficial.

5) In depth analysis of scRNAseq data is nearly exclusively shown for ISG15+CD8+ T subset. The question arises, whether the identified mechanisms are the same in other cell populations in ICIAM patients?

Reviewer #4

(Remarks to the Author)

The manuscript presented by Zhang et al. is not only beautifully presented but also a simple tour de force including all elements of a study anybody could wish for: a range of state-of-the-art technology, clear patient relevance, several distinct animal models for mechanistic explanation, thorough validation. If anything, the immense amount of data could probably be broken up into several manuscripts to make them easier to digest. I did not find any concerns and congratulate the authors to this impressive achievement.
Noteworthy results are (among others) that the authors show that ISG15+CD8+ T cells are major culprits in ICI-myocarditis, and validating aspects of this using human cohorts as well as a range of distinct mouse models. Importantly, they use patient PBMC, allowing this study to have direct translational impact as blood biomarker.
This work will be of significance to the field as this is an analysis at thus far unseen scale and depth. The generated datasets will be of immense value to the community (provided they are being deposited as per FAIR data policy).
All conclusions and claims are robustly supported by several lines of evidence. The methodology is sound and exceeds standard expectations in the field. The methods are well described. I have not discovered any flaws.
Minor comments:
(1) I recommend to reword the title to take emphasis away from one selected technology (single-cell omics). The manuscript is so much more than just yet another single-cell atlas and that should be reflected in the title.
(3) ICIAM feels like a rather clunky abbreviation for ICI-myocarditis. I suggest to spell out ICI-myocarditis for easier readability.
(2) Data availability; in the interest of open science, I believe the presented datasets should be deposited in an appropriate repository.

Reviewer #5

(Remarks to the Author)
I co-reviewed this manuscript with one of the reviewers who provided the listed reports. This is part of the Nature Communications initiative to facilitate training in peer review and to provide appropriate recognition for Early Career Researchers who co-review manuscripts.

Version 1:

Reviewer comments:

Reviewer #1

(Remarks to the Author)
The revised manuscript is substantially improved, and I appreciate the authors' efforts to address the previous comments. Several important points have been strengthened, including the contextualization of ISG15 biology, clarification of corticosteroid status at sampling, additional monocyte analyses, reanalysis of the Blum et al. dataset, and the expanded discussion of JAK-STAT inhibition.

A few issues would still benefit from further refinement. First, the causal language regarding $ISG15^{+}$ $CD8^{+}$ T cells should be moderated. The new coculture and ISG15-knockdown experiments are valuable additions and strengthen the biological plausibility of this subset. However, because these experiments remain in vitro/ex vivo, they do not fully establish subset-specific pathogenic causality in vivo. I would therefore suggest replacing terms such as "direct pathogenic role" with more cautious wording, such as "pathogenic potential" or "capacity to induce cardiomyocyte injury in vitro."

Second, the diagnostic specificity of the 8-protein panel should be framed more cautiously. The panel appears promising for distinguishing active ICI-myocarditis from the tested ICI-treated non-myocarditis controls, but it has not yet been evaluated against other irAEs, non-ICI myocarditis, acute coronary syndromes, heart failure, infection, or other inflammatory conditions. Therefore, broader claims of ICI-myocarditis specificity should be avoided or clearly presented as requiring future validation.

Finally, the manuscript would benefit from thorough professional English editing. Some typographical and grammatical issues remain, including "Basd on," "with high potential inducing cardiomyocyte apoptosis," "custom panel of 8-protein," "other ICIs-treated samples," "we used hallmark gene sets to to compare," "To be note," and "Shapley Additive exExplanations." Correcting these issues throughout the manuscript would improve clarity and readability.

Overall, the manuscript has improved considerably, but further revision would help ensure that the conclusions are appropriately balanced and that the presentation meets publication standards.

Reviewer #2

(Remarks to the Author)
I believe that the key points were answered, I have no further questions.

Reviewer #3

(Remarks to the Author)

The authors responded very broadly and adequately to the reviewer's comments.

Reviewer #4

(Remarks to the Author)
I maintain my prior assessment. This is a great manuscript. I would however like to reiterate the two points I made previously; please amend the data availability statement to reflect deposition as stated in the response to reviewers. Also ICIAM and ICI-myocarditis are now used interchangeably.

Open Access This Peer Review File is licensed under a Creative Commons Attribution 4.0 International License, which permits use, sharing, adaptation, distribution and reproduction in any medium or format, as long as you give appropriate credit to the original author(s) and the source, provide a link to the Creative Commons license, and indicate if changes were made.
In cases where reviewers are anonymous, credit should be given to 'Anonymous Referee' and the source.
The images or other third party material in this Peer Review File are included in the article's Creative Commons license, unless indicated otherwise in a credit line to the material. If material is not included in the article's Creative Commons license and your intended use is not permitted by statutory regulation or exceeds the permitted use, you will need to obtain permission directly from the copyright holder.
To view a copy of this license, visit https://creativecommons.org/licenses/by/4.0/

Reviewer #1 ICI checkpoint therapy (Remarks to the Author):

In this study, Shi-Long Zhang et al. characterize the cellular and molecular landscape of the rare but often lethal immune checkpoint inhibitor–associated myocarditis (ICIAM) in a large prospective cohort, screening nearly 20,000 patients for this uncommon complication. The authors collected peripheral blood mononuclear cells (PBMCs) and serum samples from patients during acute ICIAM, after ICIAM remission, and from ICI-treated patients who did not develop myocarditis, enabling a comprehensive analysis of the immune land-scape associated with this adverse event.

The study reveals pronounced alterations in the immune landscape during acute ICIAM, including changes in the frequencies of multiple $CD8^+$ T-cell subsets. Notably, the authors identify a distinct population of $ISG15^+$ $CD8^+$ T cells. They further demonstrate marked upregulation of the JAK–STAT signaling pathway in acute ICIAM and suggest that immune cell crosstalk is partially mediated through the CCR2–CCL2 axis. Using an in vivo mouse model, the authors show that blockade of the CCR2–CCL2 axis or inhibition of JAK–STAT signaling reduces $CD8^+$ T-cell infiltration and improves cardiac function, respectively. In addi-tion, they develop an eight-protein biomarker panel capable of distinguishing acute ICIAM samples from other ICI-treated patients and validate this panel in an independent cohort.

The management of immune-related adverse events represents a major challenge in clinical oncology and a key focus of translational oncoimmunology research. Overall, this study addresses an important and timely topic by defining immune cell profiles and intercellular communication pathways involved in acute ICIAM, while highlighting potential therapeutic targets for improved disease management. However, some sections of the manuscript are dense and repetitive. The following comments are intended to help improve the overall clarity, readability, and quality of the manuscript.

Author response: Thanks very much for the positive and constructive feedback on our manuscript. We appreciate the reviewer's recognition of the importance and timeliness of our study. In response, we have carefully revised the manuscript to improve clarity, readability, and overall quality, particularly by reducing dense and repetitive sections. All revised portions are marked in **RED** in the manuscript. Below, we provide a detailed point-by-point response to each comment.

Major Issues

1. Contextualization of ISG15 biology

ISG15 is, by definition, an interferon-stimulated gene. However, this well-established biological context is not discussed in the manuscript, and the upregulation of JAK–STAT signaling within the $ISG15^+$ $CD8^+$ T-cell subset is presented as somewhat unexpected. Including a brief overview of what is currently known in the literature about $ISG15^+$ $CD8^+$ T cells and their association with inter-feron signaling would

provide important context and help readers better interpret these findings.

Author response: We thank the reviewer for this important and constructive comment. We fully agree that ISG15 is, by definition, a canonical interferon-stimulated gene, and that its close association with interferon signaling, particularly the JAK-STAT pathway, should be explicitly contextualized to facilitate correct interpretation of our findings.

To address this, we have revised the Discussion to include a concise overview of the established ISG15 biology and to clarify that the observed signaling enrichment is biologically consistent with prior knowledge rather than surprising. In the revised Discussion (Lines 633-659), we now emphasize that $ISG15^+$ $CD8^+$ T cells represent an interferon-imprinted immune state, and we discuss relevant literature showing that ISG-expressing $CD8^+$ T-cell populations have been described in inflammatory and immunotherapy-associated contexts.

2. Rationale for focusing on $ISG15^+$ $CD8^+$ T cells
The manuscript convincingly shows a marked increase in the proportion of $ISG15^+$ $CD8^+$ T cells in acute ICIAM compared with non-ICIAM samples, suggesting that this subset may be biologically rel-evant. However, other immune cell populations also display significant frequency shifts but are not further explored. Providing a clearer rationale for prioritizing this specific subset, and commenting on whether similar analyses were performed (or considered) for other altered populations, would strengthen the interpretation of the results.

Author response: We thank the reviewer for this thoughtful comment and appreciate the opportunity to clarify our rationale.

First, we performed an unbiased global analysis of PBMC scRNA-seq data and identified six major immune cell types, namely NK cells, $CD8^+$ T cells, $CD4^+$ T cells, B cells, monocytes, and DCs. We found that, compared with no-ICIAM, act-ICIAM patients exhibited a significant increase in the proportion of $CD8^+$ T cells and significant decreases in the proportion of monocytes (**Fig. 1E**), consistent with a recent report. These global results provide the context that $CD8^+$ T cells represent the most prominently expanded compartment during active ICIAM in our dataset.

Second, to further investigate functional alterations across major cell types, we used hallmark gene sets to to compare pathway activities between the no-ICIAM and act-ICIAM groups. Intriguingly, $CD8^+$ T cells showed upregulation of a wide variety of pathway activities, including different aspects of immunology, metabolism, signalling, and proliferation (**Fig. 1F**). Furthermore, inflammatory-response and interferon-related pathways were upregulated in $CD8^+$ T cells (**Fig. 1F**). In addition, $CD4^+$ T cells, B cells, NK cells, and DCs displayed minimal changes in pathway activity. Accordingly,

subsequent analyses were focused on $CD8^+$ T cells.

Third, we focused our in-depth analysis on $CD8^+$ T cells for two reasons: 1) ICIs primarily target pathways whose targets are predominantly expressed on $CD8^+$ T cells, and 2) accumulating evidence supports a central role of $CD8^+$ T cells in the pathogenesis of ICI-associated myocarditis. Within the $CD8^+$ T cell compartment, we identified six $CD8^+$ T cell subclusters and discovered a previously underappreciated $ISG15^+CD8^+$ T cell population. Notably, $ISG15^+CD8^+$ T cells were most prominently elevated in act-ICIAM patients but markedly decreased in no-ICIAM and rem-ICIAM groups. Based on this disease-associated expansion pattern, we considered $ISG15^+CD8^+$ T cells a plausible key immune population linked to active disease and therefore prioritized mechanistic interrogation of this subset. To experimentally address this, we isolated murine $CD8^+$ T cells and cocultured them with recombinant mouse IFN-α2 (r-mIFN-α2) to induce ISG15 expression in vitro. Notably, r-mIFN-α2 stimulation led to a marked increase in the proportion of $ISG15^+$ $CD8^+$ T cells (**new Fig. 4D**). Importantly, we further explored the functional consequences of this population. In cardiomyocyte coculture assays, $ISG15^+$ $CD8^+$ T cells significantly increased cardiomyocyte apoptosis (**new Fig. 4E**), elevated reactive oxygen species (ROS) production(**new Fig. 4F**), and enhanced expression of apoptosis markers and PUMA compared with controls (**new Fig. 4G**), providing direct evidence that these cells possess pathogenic effector capacity capable of promoting myocardial injury.

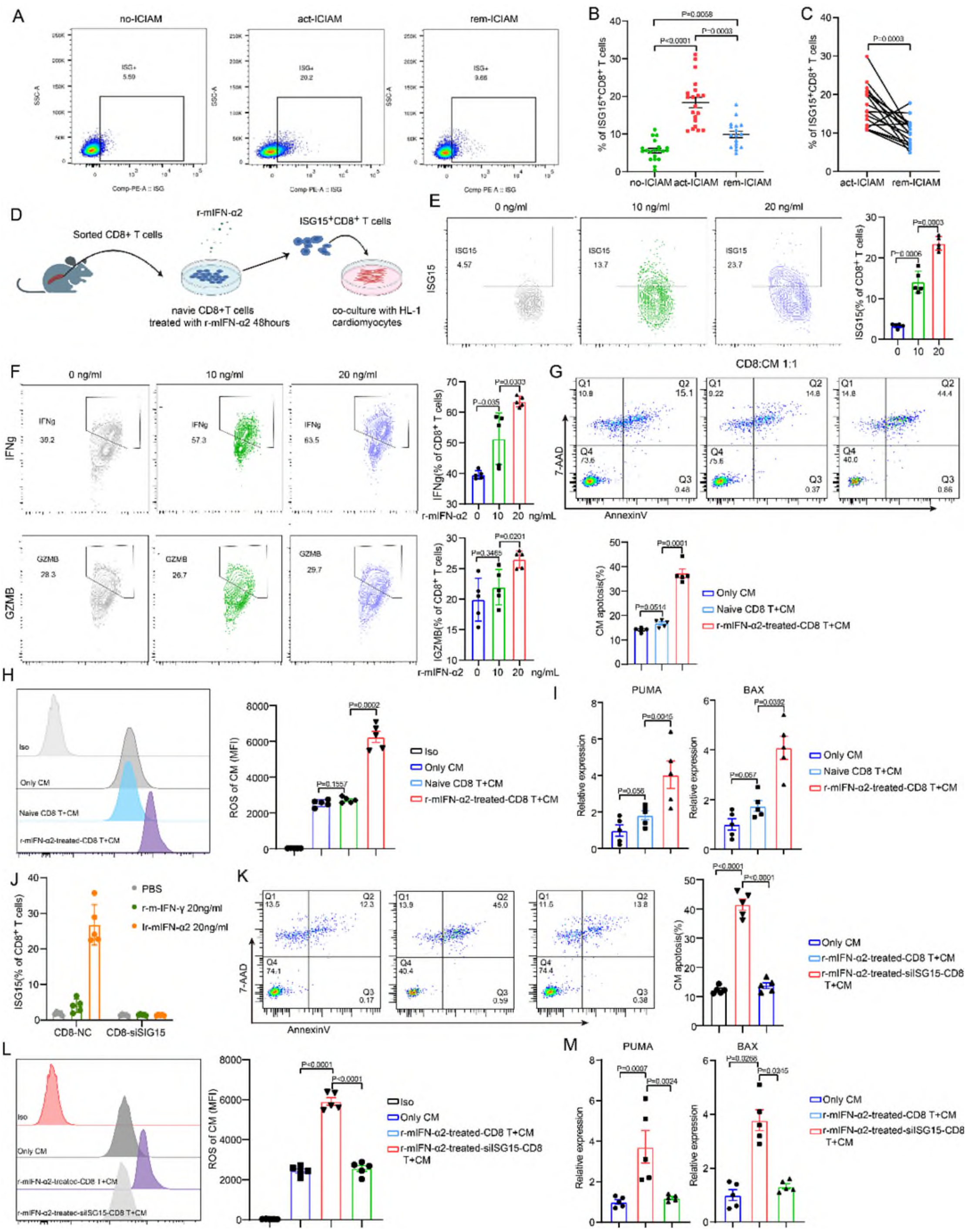

Fig. 4. ISG15⁺ CD8⁺ T cells induce cardiomyocyte apoptosis in co-culture assays.

A-B, Representative flow cytometry plots (A) and quantification (B) showing the frequency of ISG15⁺CD8⁺ T cells in the indicated groups.

C, Paired analysis showing dynamic changes in the proportion of ISG15⁺CD8⁺ T cells between act-ICIAM and rem-ICIAM samples.

D, Schematic workflow of the coculture assay between cardiomyocytes and ISG15⁺CD8⁺ T cells.

E, Flow cytometry analysis of ISG15 expression in CD8⁺ T cells following stimulation with r-mIFN-α2 at the indicated concentrations (0, 10 and 20 ng/mL).

F, Flow cytometry analysis of IFNG and GZMB expression in CD8⁺ T cells following

stimulation with r-mIFN-α2 at the indicated concentrations (0, 10 and 20 ng/mL).
G, Quantification of cardiomyocyte apoptosis after co-culture with ISG15$^+$CD8$^+$ T cells at a ratio of 1:1 (ISG15$^+$CD8$^+$ T:CM).
H, Quantitative analysis of reactive oxygen species (ROS) levels in cardiomyocytes cocultured with ISG15$^+$CD8$^+$ T cells at a ratio of 1:1.
I, Relative expression levels of BAX and PUMA in cardiomyocytes following coculture with ISG15$^+$CD8$^+$ T cells at a ratio of 1:1.
J, The specific siRNA was designed to interfere ISG15 expression in CD8$^+$ T cell and the suppressive efficacy was measured by flow cytometry assay.
K, Quantification of cardiomyocyte apoptosis after coculture with CD8$^+$ T cells following ISG15 siRNA-mediated knockdown.
L, Measurement and quantitative analysis of ROS levels in cardiomyocytes co-cultured with CD8$^+$ T cells after ISG15 siRNA transfection.
M, Relative expression of BAX and PUMA in cardiomyocytes co-cultured with CD8$^+$ T cells after ISG15 siRNA transfection.

Fourth, in direct response to the reviewer's request, we expanded our analyses to other immune compartments, focusing specifically on monocytes because they showed the most prominent compositional decrease during active disease. Unbiased single-cell analyses revealed that monocytes displayed marked changes in both pathway activity and cellular composition during active disease, ranking second only to CD8$^+$ T cells. Through the single-cell profile of monocytes, we defined five transcriptionally distinct monocyte subpopulations, including S100A12^{hi}CD14$^+$monocytes, HLA-DRB5$^+$CD14$^+$ monocytes, IFN-activated (IFN^{act}) CD14$^+$ monocytes, IL1B^{hi} CD14$^+$ monocytes, and CD16$^+$ monocytes (**new Supplementary Fig. 8**A-B). Notably, S100A12^{hi}CD14$^+$monocytes and IL1B^{hi} CD14$^+$ monocytes were increased in act-ICIAM group (**new Supplementary Fig. 8C**), and DGE indicated that S100A12^{hi}CD14$^+$monocytes was the most influenced between no-ICIAM and act-ICIAM (**new Supplementary Fig. 8D-E**). Accordingly, we revised the Results section to present the global immune landscape first, followed by parallel pathway analyses across major cell types, before focusing on ISG15$^+$CD8$^+$ T cells. And then we expanded our analyses to monocytes.

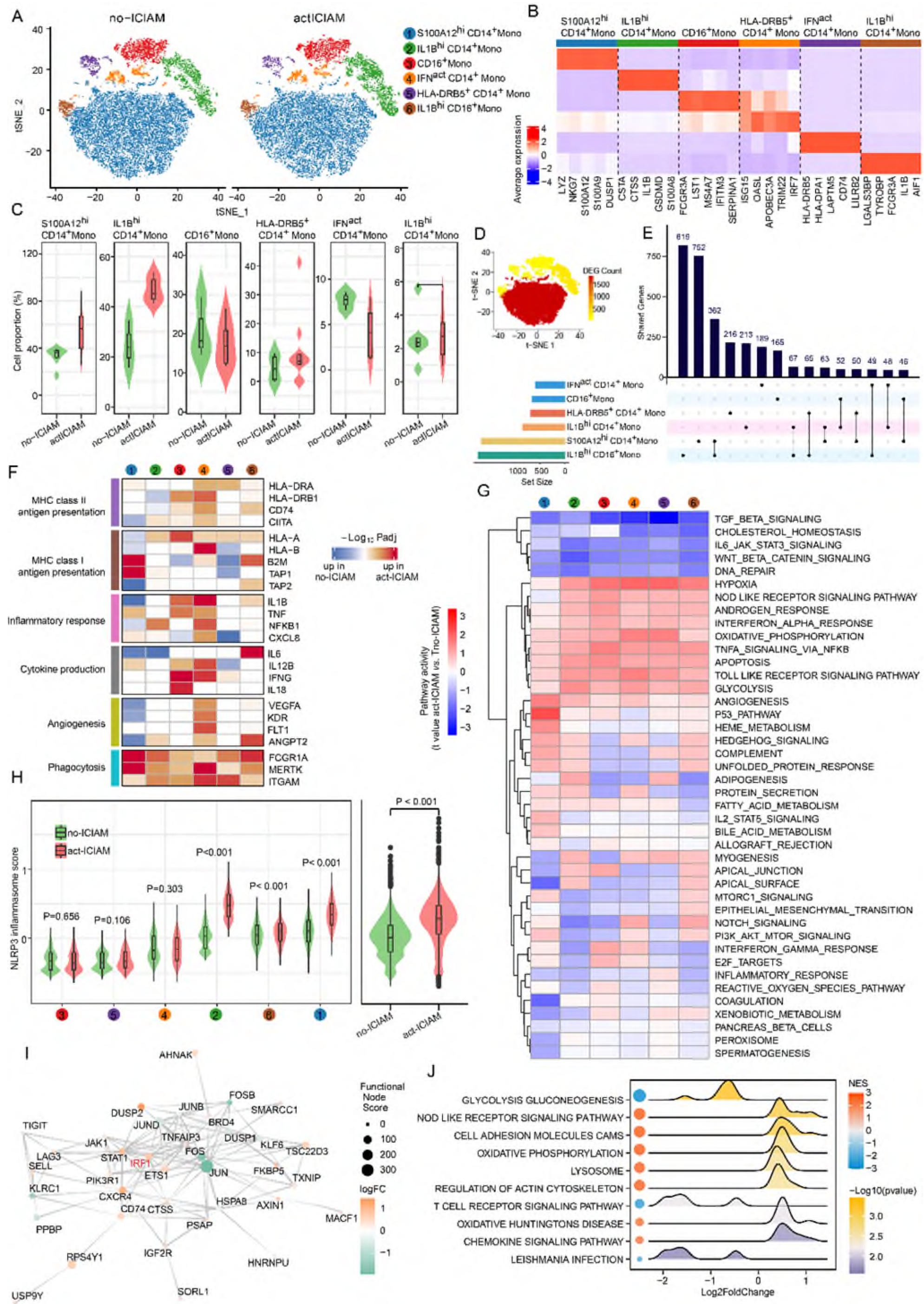

Supplementary Fig. 8. Changes in inflammatory response and cytokines in monocyte subclusters after treatment with different therapies.

A,Identification of 8 subclusters of monocytes between the no-ICIAM and act-ICIAM groups.

B, Heatmap showing the scaled expression of top 5 differentially expressed genes among 8 subclusters of monocytes.

C, Violin and box plot showing the proportion of each monocyte subcluster between the no-ICIAM and act-ICIAM groups.

D, Feature plot showing the count of DEGs determined by a two-sided Wilcoxon test.

E, UpSet plot showing all considered comparisons, with horizontal bars indicating the

number of genes in each set and vertical bars showing the intersections, revealing shared DEGs across monocyte subsets.

F, Heatmap showing the relative change in the expression level of signature genes within each monocyte subcluster between the no-ICIAM and act-ICIAM groups.

G, Differences in pathway activities scored per scored per monocytes using GSVA, between the no-ICIAM and act-ICIAM groups. t values were independent of effects from the patient of origin.

H, Violin and box plot showing the JAK-STAT signaling pathway signature score across monocyte between the no-ICIAM and act-ICIAM groups. The signature score was calculated by the AUCell algorithm.

I, Protein-protein interaction graph depicting minimal significant interaction graph of DEGs in $IL1B^{hi}CD14^{+}$ monocytes between the no-ICIAM and act-ICIAM groups (FDR $<1.0\times10^{-2}$).

J, Gene set enrichment analysis of the $IL1B^{hi}$ $CD14^{+}$ monocytes for inflammatory pathways comparing between the no-ICIAM and act-ICIAM groups.

3. Specificity of the 8-protein panel

The proposed 8-protein panel includes broadly acting immunomodulatory molecules (e.g., IFNγ, IL-6, and chemokines). While the panel effectively distinguishes acute ICIAM samples from other ICI-treated samples, it remains unclear whether it is specific for ICIAM as opposed to other immune-related adverse events (irAEs) or cardiac conditions. Testing the panel in plasma samples from patients with other irAEs, if available, would help clarify its diagnostic specificity.

Author response: We thank you for this important and thoughtful comment. In fact, our primary goal in developing this 8-protein panel was to address an unmet clinical need: the early identification of acute ICIAM among ICI-treated cancer patients, in whom timely differentiation from non-cardiac causes of symptoms is crucial. Accordingly, the panel was trained and validated specifically within an ICI-treated population, comparing acute ICIAM against non-ICIAM ICI-treated controls.

We acknowledge that the current study does not include plasma samples from patients with other irAEs, and therefore we cannot formally claim specificity for ICIAM across all irAEs. We have now explicitly acknowledged this limitation in the revised Discussion. Importantly, we now frame the 8-protein panel as a diagnostic classifier optimized for identifying acute ICIAM within the ICI-treated population, rather than as a universal irAE signature. Nevertheless, we note that the diagnostic performance of the panel arises not from any single broadly acting cytokine, but from the integrated multivariate pattern of the eight proteins, which together reflect coordinated immune activation associated with active ICIAM. This multivariate approach mitigates the nonspecificity of individual markers and enhances disease discrimination. We have

added this point to the Discussion to clearly outline the next steps required to further establish diagnostic specificity (Line 706-711).

4. Clinical applicability of the protein panel

The authors clearly outline the diagnostic challenges of ICIAM, including the need for multiple diag-nostic tools and exclusion of alternative causes, which may delay treatment and lead to myocardial damage. In this context, the development of the 8-protein panel is highly relevant. Expanding the discussion to address how this panel could be implemented in clinical practice—particularly with re-spect to feasibility, cost, turnaround time, and how it compares to current diagnostic standards—would further highlight its potential clinical impact.

Author response: We thank your comment. In response, we have expanded the Discussion to explicitly address the clinical applicability of the protein panel, including feasibility, turnaround time, and its relationship to current diagnostic tools (Line 694-706). We now clarify that the panel is designed as a plasma-based assay, requiring only routine blood sampling, and can be implemented using a multiplex Luminex platform that is compatible with standard clinical laboratory workflows. This approach enables simultaneous measurement of multiple analytes with relatively low sample volume, reasonable cost, and a turnaround time that is shorter than that of advanced imaging modalities or invasive diagnostic procedures. Importantly, we emphasize that the 8-protein panel is not intended to replace established diagnostic standards, such as cardiac biomarkers, echocardiography, or cardiac MRI. Rather, we propose its use as a complementary tool for early screening or risk stratification among ICI-treated patients. In this context, the panel could help identify patients at high risk for ICIAM at an earlier stage, prompting intensified cardiac monitoring, expedited imaging, or earlier therapy for ICIAM, thereby potentially reducing diagnostic delay and myocardial damage.

5. Causal role of ISG15$^+$ CD8$^+$ T cells

At present, the evidence provided does not fully support the conclusion that ISG15$^+$ CD8$^+$ T cells play a detrimental role in disease pathogenesis (line 336). The mouse experiments involve depletion of all peripheral CD8$^+$ T cells prior to myocarditis induction. While these data are informative, they do not specifically address the contribution of the ISG15$^+$ subset, which represents only a fraction of the total CD8$^+$ T-cell pool. This distinction should be clarified, and conclusions moderated accordingly.

Author response: We thank for this important comment and fully agree with the concern regarding causal interpretation. We also apologize that the original wording may have caused the impression that we were claiming subset-specific causality based on bulk CD8$^+$ depletion. In the revised manuscript, we have removed the corresponding

statements.

Importantly, we did attempt to directly and specifically target ISG15$^+$CD8$^+$ T cells in vivo. However, this was not feasible due to a practical technical limitation: to our knowledge, there is currently no commercially available, in vivo-validated ISG15-neutralizing antibody that would enable specific depletion of ISG15$^+$CD8$^+$ T cells in the mouse model. We respectfully ask the reviewer to understand that this limitation reflects the current tool availability in the field rather than a lack of effort. Once commercially available antibodies or small-molecule inhibitors targeting ISG15 become established and validated for in vivo use, we will further validate and extend the findings of the current study. Given this constraint, we strengthened our validation using an ex vivo functional approache[1-3] that more directly interrogate the pathogenic potential of ISG15$^+$CD8$^+$ T cells as follows:

Previous studies have established that ISG15 is a canonical interferon-stimulated gene that is robustly upregulated downstream of type I interferon signaling, and can be induced by IFN-α2[4-6]. Thus, we isolated murine splenic CD8$^+$ T cells and cocultured ex vivo, and stimulated with recombinant mouse IFN-α2 (r-mIFN-α2) to induce ISG15 expression (**new Fig. 4D**). Notably, Dose-response experiments using r-mIFN-α2 stimulation (0, 10, and 20 ng/mL) efficiently induced the differentiation of CD8$^+$ T cells into an ISG15$^+$ CD8$^+$ T cell population (**new Fig. 4E**). Importantly, we further explored the functional consequences of this population. In the context of ICI-associated myocarditis, pathogenic T cells may induce apoptosis of cardiomyocytes[7, 8]. To directly validate the cytotoxicity of ISG15$^+$ CD8$^+$ T cells on cardiomyocytes, we conducted a T cell-mediated cell-killing assay by co-culturing ISG15$^+$CD8$^+$ T cells and HL-1 cardiomyocytes. ISG15$^+$CD8$^+$ T cells cocultured with cardiomyocytes at a 1:1 ratio (CD8:CM) exhibited significantly increased apoptosis (**new Fig. 4G**), elevated reactive oxygen species (ROS) production(**new Fig. 4H**), and enhanced expression of apoptosis markers and PUMA compared with controls (**new Fig. 4I**). Notably, these cytotoxic effects remained evident even at a lower ratio of 1:10 (Supplementary Fig. 6B-C), indicating a robust cardiomyocyte-damaging capacity of ISG15$^+$CD8$^+$ T cells. To more directly investigate the role of ISG15, we knocked down ISG15 in CD8$^+$ T cells using siRNA. And we confirmed that the expression of ISG15 was significantly lower than those in the NC-siRNA groups (**new Fig. 4J**). Notably, ISG15-knockdown CD8$^+$ T cells failed to induce cardiomyocyte apoptosis, or ROS production, and were associated with markedly reduced expression of BAX and PUMA in cardiomyocytes (**new Fig. 4K-M**). These findings provide functional evidence supporting a direct pathogenic role of the ISG15$^+$ CD8$^+$ T-cell subset in ICI-associated myocarditis.

Collectively, although selective in vivo depletion of ISG15$^+$ CD8$^+$ T cells is

currently not technically feasible, these complementary functional experiments provide mechanistic evidence supporting a direct pathogenic role of the ISG15⁺ CD8⁺ T-cell subset in promoting cardiomyocyte injury during myocarditis. We have revised the manuscript to present these findings more cautiously and to clearly acknowledge the limitations of the current experimental approaches.

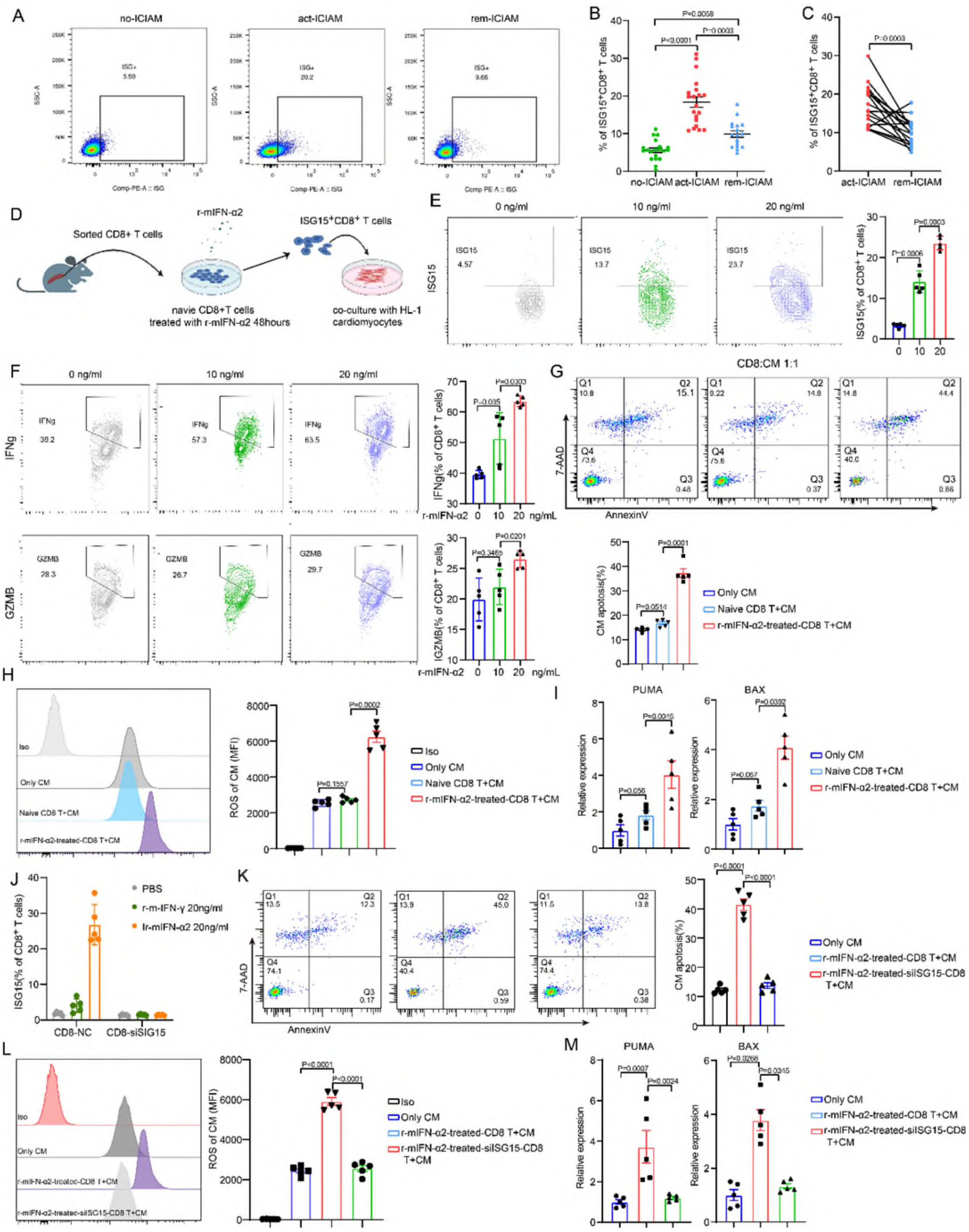

Fig. 4. ISG15⁺ CD8⁺ T cells induce cardiomyocyte apoptosis in co-culture assays. A-B, Representative flow cytometry plots (A) and quantification (B) showing the frequency of ISG15⁺CD8⁺ T cells in the indicated groups.

C, Paired analysis showing dynamic changes in the proportion of $ISG15^{+}CD8^{+}$ T cells between act-ICIAM and rem-ICIAM samples.

D, Schematic workflow of the coculture assay between cardiomyocytes and $ISG15^{+}CD8^{+}$ T cells.

E, Flow cytometry analysis of ISG15 expression in $CD8^{+}$ T cells following stimulation with r-mIFN-α2 at the indicated concentrations (0, 10 and 20 ng/mL).

F, Flow cytometry analysis of IFNG and GZMB expression in $CD8^{+}$ T cells following stimulation with r-mIFN-α2 at the indicated concentrations (0, 10 and 20 ng/mL).

G, Quantification of cardiomyocyte apoptosis after co-culture with $ISG15^{+}CD8^{+}$ T cells at a ratio of 1:1 ($ISG15^{+}CD8^{+}$ T:CM).

H, Quantitative analysis of reactive oxygen species (ROS) levels in cardiomyocytes cocultured with $ISG15^{+}CD8^{+}$ T cells at a ratio of 1:1.

I, Relative expression levels of BAX and PUMA in cardiomyocytes following coculture with $ISG15^{+}CD8^{+}$ T cells at a ratio of 1:1.

J, The specific siRNA was designed to interfere ISG15 expression in $CD8^{+}$ T cell and the suppressive efficacy was measured by flow cytometry assay.

K, Quantification of cardiomyocyte apoptosis after coculture with $CD8^{+}$ T cells following ISG15 siRNA-mediated knockdown.

L, Measurement and quantitative analysis of ROS levels in cardiomyocytes co-cultured with $CD8^{+}$ T cells after ISG15 siRNA transfection.

M, Relative expression of BAX and PUMA in cardiomyocytes co-cultured with $CD8^{+}$ T cells after ISG15 siRNA transfection.

Reference

1. Tong, Z., X. Yan, T. Chen, et al., Single-Cell Multi-Omics Identifies Specialized Cytotoxic and Migratory CD8(+) Effector T Cells in Acute Myocarditis. Circulation, 2025, 152(14): 1003-1022.
2. Zhang, L., K. Liu, X. Duan, et al., CXCL12/CXCR4 axis mediates CD8 (+) T cell overactivation in the progression of viral myocarditis. J Transl Med, 2025, 23(1): 399.
3. Santos-Zas, I., J. Lemarié, I. Zlatanova, et al., Cytotoxic CD8(+) T cells promote granzyme B-dependent adverse post-ischemic cardiac remodeling. Nat Commun, 2021, 12(1): 1483.
4. Martínez-Fleta, P., C. Pertusa, I. Fernández-Delgado, et al., ISG15 modulates inflammatory profiles and ability to activate CD8 + T cells in bone marrow-derived dendritic cells. Cell Mol Life Sci, 2025, 82(1): 362.
5. Chen, Y.L., A. Yousif, C.H. Chen, et al., Chronic ISG15 Exposure Accelerates CD8+ T-cell Dysfunction while Increasing PD-1 Blockade Sensitivity in Oral Squamous Cell Carcinoma. Cancer Immunol Res, 2025, 13(11): 1829-1844.
6. Iglesias-Guimarais, V., T. Ahrends, E. de Vries, et al., IFN-Stimulated Gene 15 Is an Alarmin that Boosts the CTL Response via an Innate, NK Cell-Dependent Route. J Immunol, 2020, 204(8): 2110-2121.
7. Huang, Y.V., Y. Sun, H. Chou, et al., Novel Therapeutic Approach Targeting CXCR3 to Treat Immunotherapy Myocarditis. Circ Res, 2025, 136(5): 473-490.

8. Axelrod, M.L., W.C. Meijers, E.M. Screever, et al., T cells specific for α-myosin drive immunotherapy-related myocarditis. Nature, 2022, 611(7937): 818-826.

6. Comparison with recent literature (Blum et al., Nature 2024)

A recent study by Blum et al. (Nature, 2024) analyzed blood and heart tissue from patients with immune-related myocarditis and identified cytotoxic $CD8^+$ T cells as key contributors to disease. This population is not discussed in the current manuscript, and some discrepancies between the blood-based findings of the two studies are apparent. Addressing these differences, proposing possible ex-planations, and—if feasible—validating key findings using this external dataset would substantially enhance the robustness and impact of the study.

Author response: We thank the reviewer for highlighting the important study by Blum et al. (Nature, 2024) and for encouraging a direct comparison with our findings. They analyzed blood and heart tissue from patients with ICI-related myocarditis and identified cytotoxic $CD8^+$ T cells as key contributors to disease. In contrast, our study focuses primarily on peripheral blood immune remodeling during active ICIAM, where we identified an expansion of $ISG15^+$ $CD8^+$ T cells characterized by a strong JAK-STAT transcriptional program. We recognize that these two $CD8^+$ T-cell states are not identical and may reflect distinct functional or temporal phases of the disease. We propose several non-mutually exclusive explanations for these apparent differences. First, sample source and biological compartment are likely major contributors: cytotoxic $CD8^+$ T cells enriched in cardiac tissue may represent terminal effector populations directly mediating myocardial injury, whereas $ISG15^+$ $CD8^+$ T cells in peripheral blood may reflect a systemic, interferon-imprinted immune activation state associated with acute disease. Second, disease stage and clinical severity may influence the dominant circulating T-cell phenotype; notably, Blum et al. reported associations between cycling $CD8^+$ T cells and fatal irMyocarditis, whereas our cohort includes patients across a broader clinical spectrum and focuses on samples collected at diagnosis prior to immunosuppressive treatment. Third, methodological differences, including cohort composition, sequencing depth, and analytical strategies, may further contribute to divergent observations.

Importantly, to directly address the reviewer's suggestion, we have leveraged publicly available single-cell dataset (GSE228597) from Blum et al. and performed an independent reanalysis using our $CD8^+$ T-cell annotation framework (**new Supplementary Fig. 5A-B**). This analysis confirmed the presence of interferon-stimulated gene-expressing $CD8^+$ T cells, including $ISG15^+$ $CD8^+$ T cells, within myocarditis samples (**new Supplementary Fig. 5C-E**). Consistent with our findings in

PBMCs, these myocardial ISG15⁺ CD8⁺ T cells exhibited a pronounced proinflammatory transcriptional program, characterized by enrichment of IL6-JAK-STAT3 signaling, IFN-γ and IFN-α responses, IL2-STAT5 signaling, and TNF signaling via NF-κB (**new Supplementary Fig. 5F**). These results have been incorporated into the revised manuscript (**new Supplementary Fig. 5**). In the Discussion, we now explicitly compare our findings with those of Blum et al. (Lines 657-659), emphasize the complementary nature of tissue- and blood-based immune profiling, and clarify that our study aims to characterize peripheral immune signatures and diagnostic biomarkers.

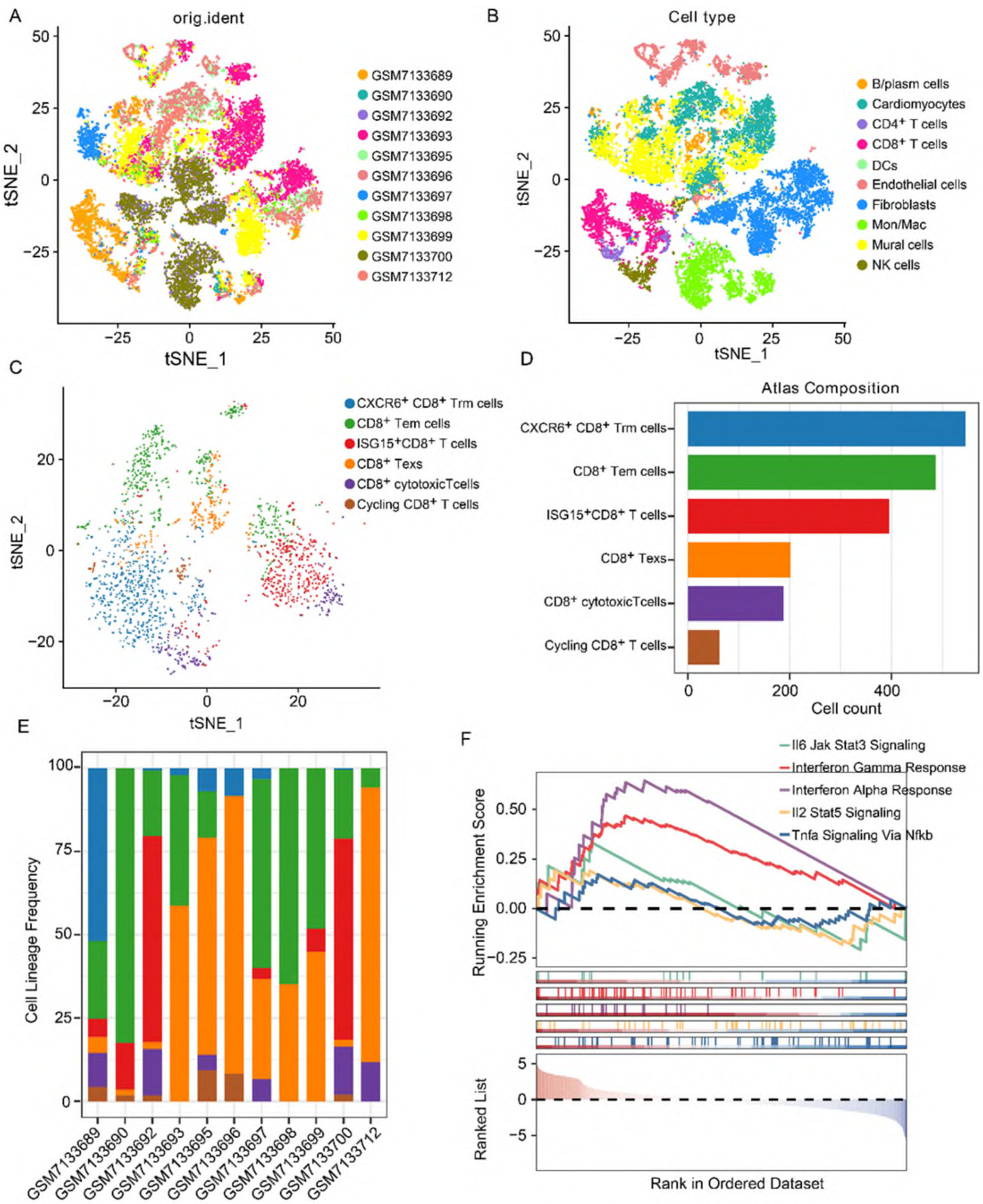

Supplementary Fig. 5. Single-cell transcriptomic analysis of cardiac tissues in the

GSE228597 dataset.

A, t-SNE plot showing the origins of individual immune cells in the cardiac tissues from no-ICIAM and ICIAM patients (n = 11).

B, t-SNE plot showing the overview of 10 cell clusters in the single-cell transcriptomes of cardiac tissues from no-ICIAM and ICIAM patients (n = 11). Clusters were named according to the indicate immune cell subsets using the canonical marker genes and were color-coded.

C, Clustering of $CD8^+$ T cells based on all the enrolled samples (n=11), colored and labeled by $CD8^+$ T cell clusters.

D-E, Bar plots showing the cell counts for each $CD8^+$T cell lineage captured (D) and the cell lineage proportions per sample (E).

F, GSEA analysis revealing pathways significantly upregulated in $ISG15^+$ $CD8^+$ T cells from act-ICIAM samples compared with no-ICIAM samples.

7. Link between blood-based findings and cardiac tissue pathology

The primary analyses are based on blood and serum samples collected during acute ICIAM. While significant immune alterations are identified, the mechanistic connection between circulating immune changes and myocardial tissue damage remains unclear. If available, additional mechanistic insights could be included; alternatively, this limitation could be explicitly acknowledged in the Discussion.

Author response: We thank your important comment. We agree that although our primary analyses are based on peripheral blood and plasma samples collected during acute ICI-associated myocarditis, the mechanistic link between circulating immune alterations and myocardial tissue pathology cannot be fully resolved from blood-based data alone. Peripheral blood profiling offers clear clinical advantages, including minimal invasiveness and feasibility for biomarker development and longitudinal monitoring. Accordingly, a central aim of our study was to characterize the peripheral immune landscape of ICIAM and to translate these findings into blood-based diagnostic biomarkers. However, circulating immune signatures primarily reflect systemic immune activation and may not fully capture the local immune microenvironment within cardiac tissue. Myocardial tissue, in contrast, directly reflects local inflammation, immune infiltration, and tissue damage, and therefore provides more definitive mechanistic insight into disease pathogenesis. However, routine acquisition of cardiac tissue in ICIAM patients is clinically impractical and ethically constrained, limiting the availability of tissue-based single-cell analyses in this setting. This represents a fundamental challenge in studying ICIAM, particularly in the acute phase.

In the Discussion, we now underscore the complementary roles of blood-based profiling for clinical translation and tissue-based analyses for mechanistic resolution, and we highlight the need for future integrative studies combining peripheral immune

monitoring with cardiac tissue, imaging, or spatially resolved approaches to more fully elucidate ICIAM pathogenesis (Lines 650-659). We are grateful to the reviewer for prompting this clarification, which has strengthened the balance and rigor of our interpretation.

8. Validation of ISG15$^+$ CD8$^+$ T cells in tissue datasets

Some findings are validated using bulk mRNA sequencing of human myocardial tissue and single-cell RNA sequencing of mouse myocardial tissue. However, given that a central conclusion of the study is the pathogenic role of ISG15$^+$ CD8$^+$ T cells, stronger validation in myocardial tissue would be valuable. Specifically, it would be helpful to clarify whether this population is detectable in the val-idation datasets or in other single-cell datasets derived from ICIAM myocardial tissue (e.g., Blum et al., 2024), rather than normal heart tissue.

Author response: We thank you for this insightful and constructive suggestion. Following your suggestion, we reanalyzed the single-cell dataset from the study by Blum et al., which includes heart samples from 10 ICIAM patients and one healthy donor (GSE228597). Using the same analytical framework and marker-based annotation strategy established in our study, we identified 19,032 high-quality cells and resolved 10 major cardiac and immune cell lineages. Importantly, focused subclustering of the CD8$^+$ T cell revealed a distinct ISG15$^+$ CD8$^+$ T cell population in the cardiac tissues. Consistent with our findings in PBMCs, these ISG15$^+$ CD8$^+$ T cells exhibited a pronounced proinflammatory transcriptional program, characterized by enrichment of IL6-JAK-STAT3 signaling, IFN-γ and IFN-α responses, IL2-STAT5 signaling, and TNF signaling via NF-κB. These results provide independent, tissue-level validation that ISG15$^+$ CD8$^+$ T cells are not only detectable in affected human hearts but also retain the JAK-STAT-driven, inflammatory phenotype observed in circulation.

We have now incorporated these analyses and results into the revised manuscript (**new Supplementary Fig. 5**). Many thanks again.

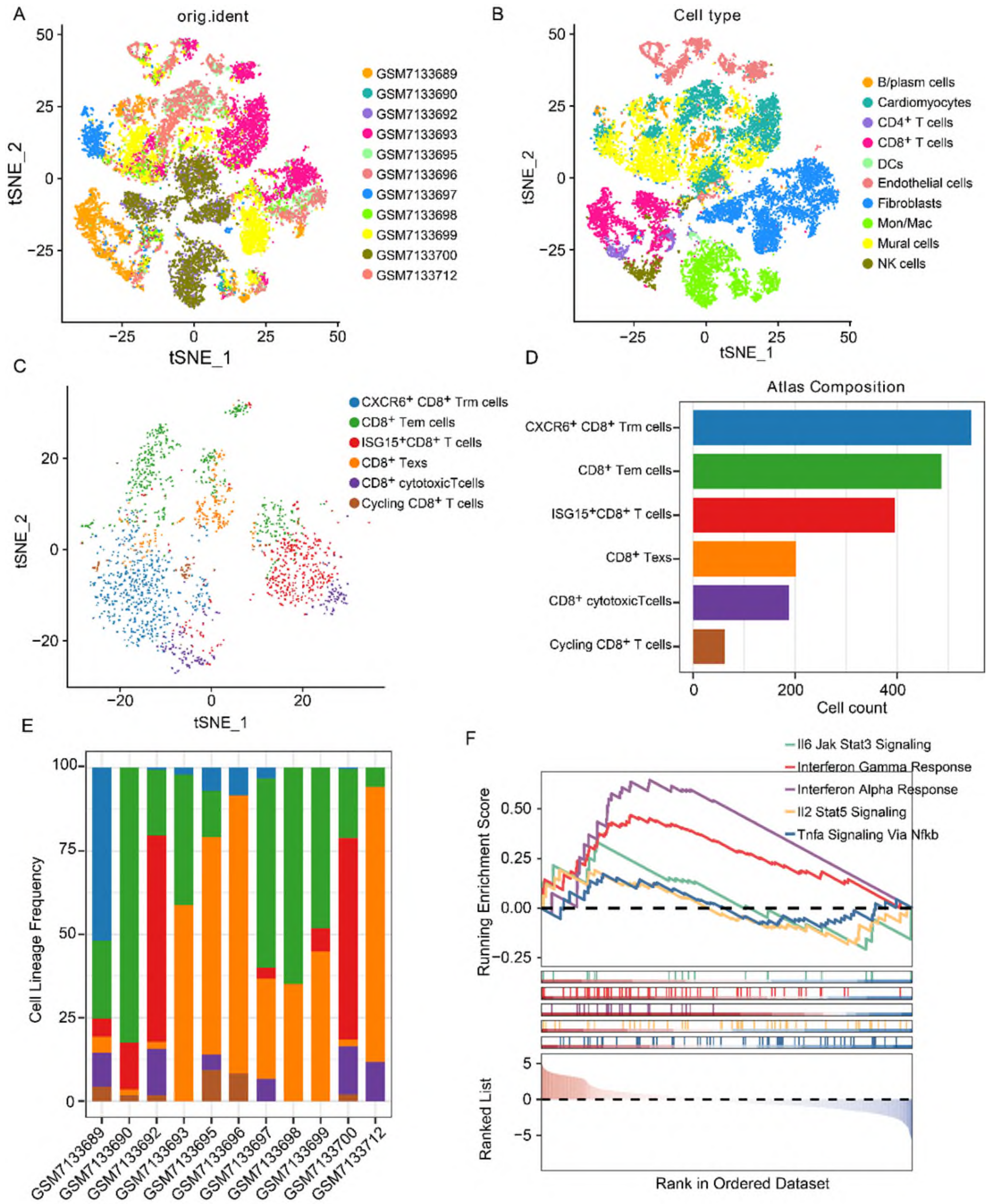

Supplementary Fig. 5. Single-cell transcriptomic analysis of cardiac tissues in the GSE228597 dataset.

A, t-SNE plot showing the origins of individual immune cells in the cardiac tissues from no-ICIAM and ICIAM patients (n = 11).

B, t-SNE plot showing the overview of 10 cell clusters in the single-cell transcriptomes of cardiac tissues from no-ICIAM and ICIAM patients (n = 11). Clusters were named according to the indicate immune cell subsets using the canonical marker genes and were color-coded.

C, Clustering of $CD8^+$ T cells based on all the enrolled samples (n=11), colored and labeled by $CD8^+$ T cell clusters.

D-E, Bar plots showing the cell counts for each $CD8^+$T cell lineage captured (D) and

the cell lineage proportions per sample (E).
F, GSEA analysis revealing pathways significantly upregulated in ISG15^{+} CD8^{+} T cells from act-ICIAM samples compared with no-ICIAM samples.

9. Interpretation of JAK–STAT pathway inhibition

The JAK–STAT pathway is widely recognized for its roles in cell survival, immune regulation, and, in some contexts, immune escape mechanisms (e.g., PD-L1 upregulation in tumors). Given these known functions, further discussion of the potential risks, limitations, and broader implications of in-hibiting this pathway—particularly in cancer patients—would be important.

Author response: We thank you for this thoughtful comment. In response, we have substantially revised and expanded the Discussion to explicitly address the potential risks, limitations, and broader implications of JAK-STAT inhibition (Lines 667-678). We now emphasize that, while aberrant JAK-STAT activation is associated with ICI-induced adverse events, this pathway also contributes to antitumor immunity in a context-dependent manner. Accordingly, indiscriminate inhibition may carry theoretical risks of impairing tumor control. To address this concern experimentally, we evaluated the impact of JAK inhibition on antitumor immunity using tumor-bearing mouse models with and without myocarditis. Importantly, we found that tofacitinib did not diminish antitumor efficacy and, when combined with anti-PD-1 therapy, even enhanced tumor control in the MC38 tumor model (**Fig. 8**). These findings are consistent with emerging clinical and preclinical evidence suggesting that JAK inhibitors can enhance ICI therapy in solid tumors. In the revised Discussion, we clearly state that JAK-STAT inhibition should be considered a targeted, context-dependent strategy for managing ICI-induced toxicity rather than a universally applicable adjunct to ICIs therapy. We also highlight the need for future prospective studies to establish optimal timing, dosing, and patient selection to balance toxicity control with preservation of durable tumor control (Lines 683-686).

10. Steroid treatment status

Please clarify whether patients with active ICIAM were receiving corticosteroid treatment at the time of sample collection, as this could significantly influence immune profiles and should be accounted for in the interpretation of the data.

Author response: We thank you for raising this point. We would like to clarify that all samples classified as "act-ICIAM" were collected at the time of myocarditis diagnosis, prior to the initiation of corticosteroid treatment. To avoid any ambiguity, we have now

explicitly stated this information in the revised manuscript. In the **Methods** section, we clarify that act-ICIAM patients provided PBMC samples prior to the initiation of any corticosteroid or immunosuppressive treatment (Lines 779-780). In the **Results** section, we further specify that PBMCs from act-ICIAM samples were obtained before corticosteroid administration (Lines 180-181).

11. Clarifications and interpretative questions

o Line 391: Please specify whether this refers to the discovery or validation cohort.

o Line 431: “Optimal predictive model” should be defined more clearly—optimal compared to which alternatives?

o While interferon signaling and T-cell infiltration are associated with inflammation, the mecha-nistic link to ICIAM development remains unclear. Are ISG15^{+} CD8^{+} T cells driving myocar-ditis, or does an inflamed myocardium promote IFNγ production and secondary ISG15 ex-pression in infiltrating T cells? Clarifying/discussing this point would improve mechanistic un-derstanding.

Author response: Thanks for the suggestion. We feel very sorry and the inadequate formulation has brought you trouble.

In fact, Line 391 refers specifically to the Luminex assay cohort. To avoid ambiguity, we have revised the manuscript to explicitly state that the analyses described at this point were performed in the Luminex assay cohort (new Line 497).

In Line 431, we systematically evaluated seven machine-learning algorithms—including KNN, logistic regression, SVM, RPART, Naive Bayes, NNET, and random forest—for their ability to predict ICIAM. Model performance was primarily assessed using the AUC, along with calibration and clinical applicability metrics. Among all candidate models, the random forest model consistently achieved the maximum AUC value and demonstrated superior overall performance. Accordingly, we have therefore revised the manuscript to clarify that the “optimal predictive model” refers specifically to the random forest model, which was selected based on its superior performance compared with the other algorithms (new Lines 501-509).

We thank the reviewer for this insightful comment. Previous studies have established that ISG15 is a canonical interferon-stimulated gene that is robustly upregulated downstream of type I interferon signaling, and can also be induced by IFN-γ-associated inflammatory contexts. This raises the possibility that IFN-γ-rich inflammatory microenvironments may promote ISG15 expression in infiltrating CD8^{+} T cells. To experimentally address this, we isolated murine CD8^{+} T cells and cocultured them with recombinant mouse IFN-α2 (r-mIFN-α2) in vitro. Notably, r-mIFN-α2

stimulation led to a marked increase in the proportion of ISG15⁺CD8⁺ T cells, supporting the notion that interferon signaling can directly induce ISG15 expression in CD8⁺ T cells. Importantly, we further explored the functional consequences of this population. In cardiomyocyte coculture assays, ISG15⁺CD8⁺ T cells significantly increased cardiomyocyte apoptosis (**new Figure 4G**), providing direct evidence that these cells possess pathogenic effector capacity capable of promoting myocardial injury. These findings support a model in which ISG15⁺ CD8⁺ T cells are not merely passive markers of inflammation, but can actively contribute to myocardial damage. Furthermore, we have revised the Discussion to clarify this mechanistic framework (Lines 632-659).

Minor Issues

• Marker genes identified by DEG analysis (FindAllMarkers) for each T-cell cluster should be provided.

Author response: We thank you for this helpful suggestion. In response, we performed differential gene expression analysis using FindAllMarkers and visualized the expression of differentially expressed genes across T-cell cluster using dot plots. These results have now been added as **Supplementary Fig 2**.

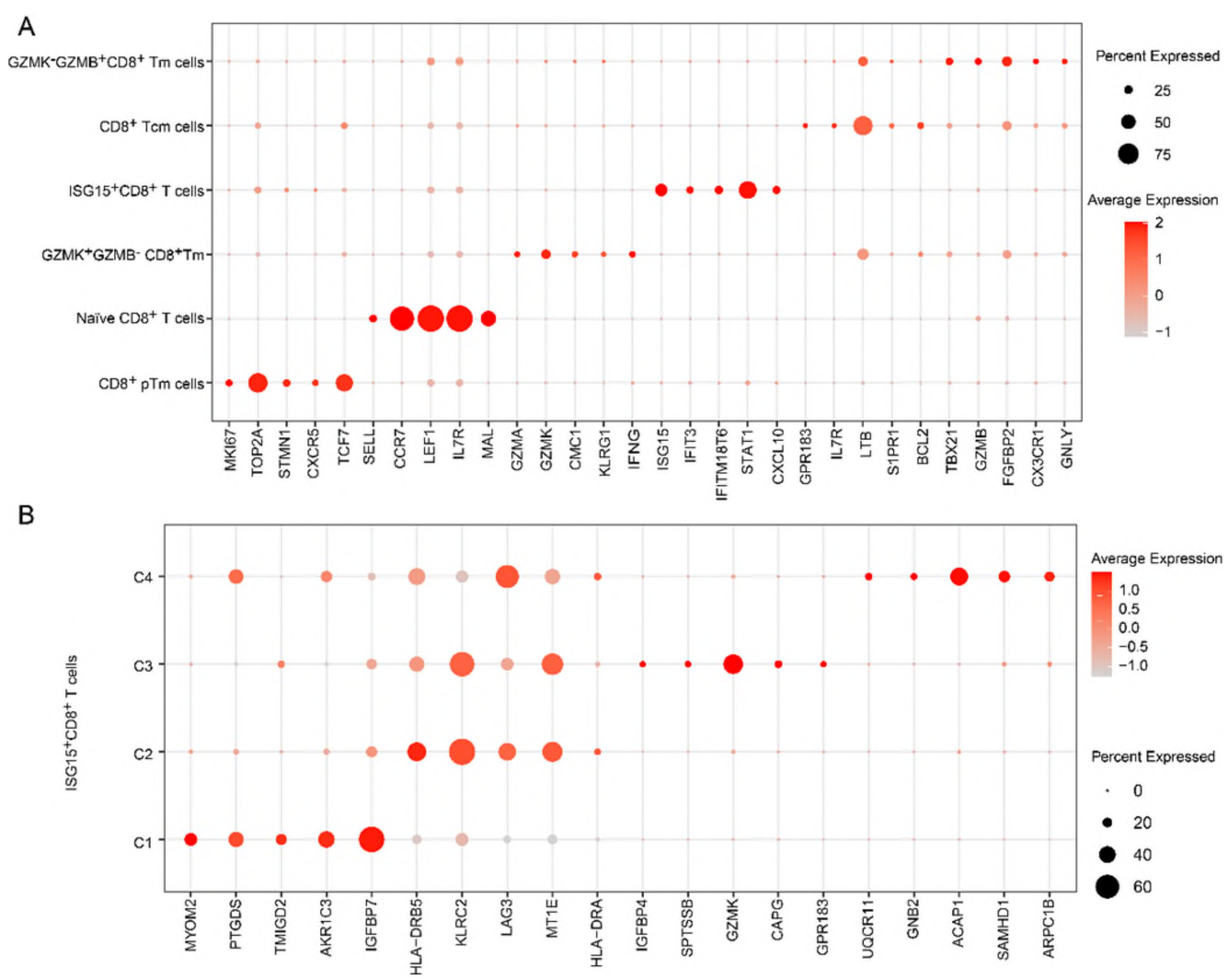

Supplementary Fig. 2. Dot plots show the expression of canonical marker genes across different clusters, including CD8⁺ T cell subclusters (A), and ISG15⁺CD8⁺T cell

subclusters (B).

• The repeated listing of techniques (scRNA-seq, flow cytometry, Olink, multiplex cytokine assays; e.g., lines 181 and 208) could be streamlined by introducing methods only when their corresponding data are first presented.

Author response: Thanks for the suggestion. As the methods are first introduced at line 181 where the corresponding data are initially presented, we have removed the redundant listing at line 208 to avoid repetition.

• t-SNE plots are shown in the figures but not described in the Methods section.

Author response: Thank for pointing out this omission. We have now revised the Methods to clearly state that the t-SNE or UMAP method was applied to visualize a 2D projection of identified clusters from the shared nearest neighbor (SNN) graph (Lines 833-836).

• The section beginning at line 301 contains results that do not fully align with the section title; subdividing the section based on the underlying rationale may improve clarity.

Author response: Thanks you for this constructive comment. we have reorganized this section into two clearly defined subsections: 1) Determinants of act-ICIAM from peripheral immune cell populations (new Lines 306); and 2) ISG15^{+} CD8^{+} T cells had direct cytotoxic effects on cardiomyocytes (new Lines 360). This reorganization better aligns the section structure with the underlying biological rationale and improves the logical flow of the Results.

• Figure 1E: The figure description does not match the text.

Author response: We thank you for pointing out this inconsistency. We have carefully revised the text describing Figure 1E to ensure that it now accurately reflects the data shown in the figure, including the direction and magnitude of changes in immune cell proportions across the no-ICIAM, act-ICIAM, and rem-ICIAM groups (Lines 210-213). Please check it.

• Figures 2A and 2G: Please provide dot plots showing DEG expression by cluster.

Author response: Many thanks for your helpful suggestion. In response, we

performed differential gene expression analysis using FindAllMarkers and visualized the expression of differentially expressed genes across clusters using dot plots. These results have now been added as **Supplementary Fig. 2A-B**.

A reference to Figure 2E is missing in the text.

Author response: We thank the reviewer for pointing out this omission and apologize for the oversight. We have corrected the text accordingly and now explicitly cite **Figure 2E**, with appropriate reference to the established framework of T cell dysfunction as described by Thommen and Schumacher[1].

Reference

1. Thommen DS, Schumacher TN. T Cell Dysfunction in Cancer. *Cancer cell*. 2018;33:547-562. doi: 10.1016/j.ccell.2018.03.012

The readability of the text in Figure 3A could be improved.

Author response: We thank the reviewer for this helpful comment. Accordingly, we have revised Figure 3A by optimizing text size, spacing, and label placement to enhance clarity and legibility. In addition, we have slightly streamlined the accompanying description in the Results of Figure 3A to improve readability (Lines 269-272).

Figure 3H: Clusters are labeled as 0–3 in the legend but elsewhere as C1–C4; consistent labeling is recommended. Alternatively, the legend could be simplified.

Author response: Thank you for pointing out this inconsistency. We sincerely apologize for the oversight. We have revised the Figure 3H to ensure consistent labeling throughout, with clusters uniformly denoted as C1-C4, and simplified the legend accordingly to improve clarity.

Line 381: Please clearly indicate that the following section refers to the mouse model.

Author response: We thank for this helpful suggestion. To improve clarity and avoid ambiguity, we have revised the text to explicitly specify that these experiments were performed in the ICIAM mouse model (Lines 418-421).

Figure 6A: The legend colors for “group” do not match those used in the figure.

Author response: Thank you for pointing out this inconsistency and we have corrected Figure 6A so that the legend now accurately matches the colors used for each group. Furthermore, we have also carefully checked and ensured color-legend consistency for other clinical variables throughout the manuscript.

Figure 7C: Increasing color contrast would improve discrimination between overlapping cell types (e.g., endothelial cells and M1 macrophages). Figure 7E: Label readability could be improved. Figure 7F: A legend description is missing.

Author response: We thank you for these helpful suggestions. In Figure 7C, we have revised the color scheme to further increase contrast between overlapping cell populations and improve visual discrimination, and we have added numeric labels directly on the UMAP plot to facilitate clearer identification of cell subpopulations, with corresponding identities provided in the legend. For Figure 7E, we have improved label readability by optimizing font size and layout. The x-axis represents different cell clusters, which are color-coded to match Figure 7C, and we have added numeric labels on the x-axis to clearly indicate each cell population. In Figure 7F, we have added a complete legend description to clarify the presented data.

• Several typographical errors are present (e.g., line 176 missing parenthesis; line 204 “analyzed” in-stead of “analyzing”; line 504).

Author response: We thank for carefully identifying these typographical errors. We have corrected the issues noted (line 176, 204, and 504). Furthermore, we have also conducted a thorough review of the entire manuscript to identify and correct any additional typographical or formatting errors.

• (Optional) Please comment on whether blockade of the CCL2–CCR2 axis affects tumor growth.

Author response: We thank the reviewer for this important question regarding the potential impact of CCL2-CCR2 axis blockade on tumor growth. Here is our comment regarding whether the blockade of the CCL2-CCR2 axis affects tumor growth:

CCL2 is highly expressed in various cancer types and has been shown to be associated with poor prognosis[1-3]. Inhibition or blockade of the CCL2/CCR2 signaling axis has thus been an area of interest for cancer therapy. Although tumor growth was not a primary endpoint of our in vivo study, previous studies indicates that inhibition of the CCL2-CCR2 pathway does not impair antitumor immunity and, in many contexts, may instead exert an antitumor role. Increasing studies have demonstrated that CCL2-CCR2 signaling promotes tumor progression by recruiting inflammatory monocytes[4]

and tumor-associated macrophages[5], vascular permeability[6], and tumor cell extravasation[7]. Genetic or pharmacologic disruption of this axis can inhibit tumor growth and metastasis in multiple cancer models, including breast cancer[4], hepatocellular carcinoma[5], cholangiocarcinoma[8], and HER2-driven mammary tumors[9]. Mechanistically, blockade of CCL2-CCR2 signaling limits monocyte/macrophage recruitment, reduces immunosuppressive M2-like macrophage polarization, and subsequently enhance $CD8^+$ T-cell-mediated antitumor immunity.

Importantly, Tu MM,et al.[3] have demonstrated that across multiple murine tumor and metastasis models that CCR2 antagonism in combination with anti-PD-1 therapy leads to sensitization and enhanced tumor response over anti-PD-1 monotherapy. And the enhanced treatment response is correlated with enhanced $CD8^+$ T cell recruitment and activation and a concomitant decrease in $CD4^+$ regulatory T cell. Collectively, these results provide strong preclinical rationale for further clinical exploration of combining CCR2 antagonism with PD-1/PD-L1-directed immunotherapies across multiple tumor types. In the context of our study, we therefore interpret CCR2 blockade primarily as a strategy to attenuate pathological immune cell trafficking in ICIAM, while acknowledging that such intervention is not expected to adversely affect tumor growth.

References

1. Li, L., Y.D. Liu, Y.T. Zhan, et al., High levels of CCL2 or CCL4 in the tumor microenvironment predict unfavorable survival in lung adenocarcinoma. Thorac Cancer, 2018, 9(7): 775-784.
2. Ueno, T., M. Toi, H. Saji, et al., Significance of macrophage chemoattractant protein-1 in macrophage recruitment, angiogenesis, and survival in human breast cancer. Clin Cancer Res, 2000, 6(8): 3282-9.
3. Tu, M.M., H.A. Abdel-Hafiz, R.T. Jones, et al., Inhibition of the CCL2 receptor, CCR2, enhances tumor response to immune checkpoint therapy. Commun Biol, 2020, 3(1): 720.
4. Qian, B.Z., J. Li, H. Zhang, et al., CCL2 recruits inflammatory monocytes to facilitate breast-tumour metastasis. Nature, 2011, 475(7355): 222-5.
5. Li, X., W. Yao, Y. Yuan, et al., Targeting of tumour-infiltrating macrophages via CCL2/CCR2 signalling as a therapeutic strategy against hepatocellular carcinoma. Gut, 2017, 66(1): 157-167.
6. Roblek, M., M. Calin, M. Schlesinger, et al., Targeted delivery of CCR2 antagonist to activated pulmonary endothelium prevents metastasis. J Control Release, 2015, 220(Pt A): 341-347.
7. Lim, S.Y., A.E. Yuzhalin, A.N. Gordon-Weeks, et al., Targeting the CCL2-CCR2 signaling axis in cancer metastasis. Oncotarget, 2016, 7(19): 28697-710.
8. Zabransky, D.J., E. Kartalia, J.W. Lee, et al., Tumor-derived CCL2 drives tumor growth and immunosuppression in IDH1- mutant cholangiocarcinoma.

Hepatology, 2025, 82(3): 599-611.

9. Chen, X., Y. Wang, D. Nelson, et al., CCL2/CCR2 Regulates the Tumor Microenvironment in HER-2/neu-Driven Mammary Carcinomas in Mice. PLoS One, 2016, 11(11): e0165595.

QC preprocessing cutoffs are repeated (lines 699 and 709).

Author response: We thank you for pointing out this redundancy. To avoid repetition, we have removed the duplicated description of QC preprocessing cutoffs at line 709, retaining a single, clear description earlier in the Methods section.

Olink is a well-established technique and does not require a detailed methodological explanation.

Author response: We agree with this suggestion. And we have streamlined and simplified the Olink-related methods section, retaining only essential information relevant to study reproducibility and interpretation (Lines 924-933).

Line 805: Please provide a table listing the antibodies used.

Author response: We thank the reviewer for this helpful suggestion. In response, we have now added a listing all antibodies used, including antibody name, clone, supplier, and catalog number to ensure clarity and reproducibility (Lines 919-923).

Reviewer #2 scRNA sequencing (Remarks to the Author):

Overall, Zhang et al. provide interesting data on ICIAM by employing single-cell omic methods as well as circulating plasma proteomics. They confirm key findings by reverse-translational approaches to the mouse model, showing that the CCL2-CCR2 axis recruits distinct, pathogenic ISG15+ CD8+ T-cell subsets which associate with adverse outcomes. The depletion of either rescues disease severity. Interestingly, inhibiton of cardiac toxicity of PD1 inhibitors by targetting culprit JAK/Stat signalling simultaneously boosters anti-tumor activity of PD1 inhibition.

This is an interesting study, yet requires major further improvements as it – as it currently stands – is rather preliminary yet.

Author response: We sincerely thank you for the thoughtful and balanced evaluation of our work. At the same time, we take the reviewer's concern regarding the preliminary nature of the study very seriously. In response, we have substantially strengthened the manuscript by expanding sample size, mechanistic validation, clarifying temporal and treatment-related confounders, and improving the interpretability of key figures and analyses. These revisions aim to more clearly delineate causality, enhance biological rigor, and better position our findings within the current landscape of ICI-related cardiotoxicity research. All revised portions are marked in **RED** in the manuscript. We believe that these additions significantly reinforce the depth and translational relevance of the study, and we are grateful to the reviewer for helping us improve the overall quality and clarity of the work. Below, we provide a detailed point-by-point response to each comment.

1. Statistics in Figure 1 includes too few patients with too high variability, a higher number of patients should be included at least for a FACS based validation in an independent validation cohort, focussing on $ISG15^+$ $CD8^+$ T-cells

Author response: Thank you for highlighting this issue. In fact, ICIAM is a rare yet highly lethal immune-related adverse event, with a reported incidence ranging from approximately 0.09-2.4%, which makes prospective biospecimen collection exceptionally challenging and renders each well-annotated sample extremely valuable. To our knowledge, our study represents one of the largest and most comprehensive patient-based investigations of ICIAM to date, both in terms of patient number and multi-omic depth. Our cohort integrates peripheral blood and myocardial tissue analyses and combines single-cell transcriptomics, plasma proteomics, and flow cytometry, providing convergent evidence across multiple experimental layers. Notably, the overall sample size and analytical breadth are comparable to, or exceed, those reported in recent high-impact ICIAM studies, including a recent report published in

Nature (*Nature.2024 Dec;636(8041):215-223.*). Importantly, the sample size in our study is also in line with, or larger than, those used in recent single-cell studies of immune-related adverse events more broadly. For example, a recent *Nature Medicine* study investigating ICI-associated colitis from 13 patients using scRNA-seq, highlighting that, for rare irAEs, biological insight is often driven by deep, multi-layered profiling rather than large cohort size alone(*Nat Med. 2024 May;30(5):1349-1362.*). Nevertheless, to directly address this concern, we have made substantial efforts to expand validation and strengthen the robustness of our findings.

First, as displayed in the study overview (**Fig. 1A**), we have further collected PBMC samples from an additional 37 patients, thereby expanding the internal cohort used for flow cytometry. As a result, a total of 57 patients were included for FACS analysis of ISG15$^+$CD8$^+$ T cells in the revised manuscript (**new Fig. 4A-C**). This expanded sample size substantially improves statistical power and reduces the impact of inter-individual variability.

Second, in response to Reviewer #1's suggestion, we leveraged an independent external dataset to provide orthogonal validation. Specifically, we reanalyzed the single-cell RNA-seq data from Blum et al. (*Nature, 2024, December; 636(8041): 215-223*) with the GEO accession number GSE228597, which comprises 11 myocarditis cardiac tissue samples. As a result, we identified an ISG15$^+$ CD8$^+$ T cell population within myocardial tissue. Furthermore, similar to the results for PBMCs, ISG15$^+$CD8$^+$ T cells in the ICIAM myocardial tissues also demonstrated proinflammatory features, including IL6-JAK-STAT3, IFN-γ response, IFN-α response, IL2-STAT5 and TNF signaling through NF-κB pathways (**new Supplementary Fig. 5**). This independent dataset confirms the presence and enrichment of ISG15$^+$ CD8$^+$ T cells in myocarditis, extending our blood-based findings to the tissue level.

Together, by expanding the internal flow cytometry cohort and integrating an independent external single-cell dataset, we provide convergent evidence supporting the upregulation of ISG15$^+$ CD8$^+$ T cells in ICIAM patients.

2. Would it be possible that the corticosteroids/treatment of ICI affect the responses

Author response: We appreciate this important question. We would like to clarify that all samples in the act-ICIAM group were collected at the time of myocarditis diagnosis and prior to the initiation of systemic corticosteroid therapy (Lines 180-181). Therefore, the immune signatures reported for act-ICIAM reflect the untreated disease state. In addition, ICI therapy was held at the time of suspected/confirmed ICI myocarditis, in accordance with major cardio-oncology guidance recommending immediate

interruption of ICI upon suspicion of ICIAM. Thus, the post-diagnosis immune profiles were not confounded by ongoing ICI administration.

3. Figure 2 does not employ proper statistics per patient but regards all cells from a conditiion as individual replicates, this should be changed.

Author response: Thank you for your valuable comments egarding statistical rigor and the unit of replication. We would like to clarify that patient-level statistics were applied where appropriate, and that cells were not treated as independent biological replicates for group-level comparisons.

For **Figure 2D**, the boxplots depict the proportions of specific T-cell subpopulations calculated at the individual patient level, including $CD4^+$ Tcm cells, $ISG15^+CD8^+$ T cells, $CD4^+$ Tfh cells, naïve $CD8^+$ T cells, and $CD8^+$ Tcm cells. Each data point represents one patient from the no-ICIAM (n = 6), act-ICIAM (n = 10), or rem-ICIAM (n = 3) group. Group comparisons were performed using a two-sided Wilcoxon rank-sum test, ensuring that statistical inference was conducted at the patient level rather than the single-cell level.

For **Figure 2I**, the analysis addresses a distinct biological question, namely the differences in progenitor-exhausted and terminally exhausted transcriptional programs among four transcriptionally defined subsets of $ISG15^+CD8^+$ T cells within the act-ICIAM group. In this panel, $ISG15^+CD8^+$ T cells were first extracted from act-ICIAM samples and subsequently subdivided into four subsets based on unsupervised clustering. Signature scores were then compared between these cell subsets using a two-sided Wilcoxon test, an approach that is commonly applied for within-population, cell-state-level comparisons and is consistent with prior high-impact single-cell studies, including *Nat Med* (2024 May;30(5):1349-1362), *Cancer Cell* (2023 Nov 13;41(11):1852-1870.e9) and *Nature Communications* (2024 Mar 7;15(1):2107).

In addition, all other panels in the Figure 2 are descriptive in nature and do not involve statistical testing. To avoid any potential misunderstanding regarding the unit of replication, we have revised the figure legends to explicitly state the statistical unit and tests applied, and we have added overlaid scatter points to visually indicate patient-level data.

We believe these clarifications address the reviewer's concern and confirm that the statistical analyses are appropriate for the biological questions posed in each panel.

4. The concept of a exploratory and validation cohort is not correct. Comparing cell-specific expression levels with circulating cytokines is far-fetched. The validation cohort is proteomics based and the exploratory cohort is cell based. This should be rephrased.

Author response: We thank the reviewer for this important comment and agree that the terminology of "exploratory" and "validation" cohorts was not appropriate in this context. As illustrated in the study overview (**Fig. 1A**), owing to limited sample

availability and the distinct material requirements across platforms, not all assays were performed for each patient. Therefore, samples were allocated into assay-specific analytical cohorts rather than conventional exploratory/validation groups. Specifically, these included a single-cell transcriptomic cohort comprising an in-house scRNA-seq dataset (n = 19, Supplementary Table 1) and a publicly available scRNA-seq dataset (n = 11), a flow cytometry cohort (n = 57, Supplementary Table 2), and a Luminex assay cohort (n = 68, Supplementary Table 3). We have revised the manuscript accordingly to clarify this point and avoid potential misunderstanding (Lines 180-191).

5. Language should be improved throughout the complete manuscript

Author response: Thanks for the suggestion. We feel very sorry and the inadequate formulation has brought you trouble. According to your advice, we have carefully modified our manuscript. All grammatical errors, most confusing, awkward, or wordy sentences have carefully edited and revised. These changes will not influence the content and framework of the paper.

6. The claim that depleting CD8+ T-cells in total corresponds to the depletion of ISG15+ CD8+ T-cells is too far fetched. Different CD8+ T-cell subsets might have fully opposite roles, hence depleting CD8+ T-cells in total is solely an approximation.

Author response: We thank your insightful comment and we fully agree with the central point. We also apologize that the original wording may have caused the impression that we were claiming subset-specific causality based on bulk $CD8^+$ depletion. In the revised manuscript, we have removed the corresponding statements.

Importantly, we did attempt to directly and specifically target $ISG15^+CD8^+$ T cells in vivo. However, this was not feasible due to a practical technical limitation: to our knowledge, there is currently no commercially available, in vivo-validated ISG15-neutralizing antibody that would enable specific depletion of $ISG15^+CD8^+$ T cells in the mouse model. We respectfully ask the reviewer to understand that this limitation reflects the current tool availability in the field rather than a lack of effort. Once commercially available antibodies or small-molecule inhibitors targeting ISG15 become established and validated for in vivo use, we will further validate and extend the findings of the current study. Given this constraint, we strengthened our validation using an ex vivo functional approache[1-3] that more directly interrogate the pathogenic potential of $ISG15^+CD8^+$ T cells as follows:

Previous studies have established that ISG15 is a canonical interferon-stimulated gene that is robustly upregulated downstream of type I interferon signaling, and can be

induced by IFN-α2[4-6]. Thus, we isolated murine splenic CD8$^+$ T cells and cocultured ex vivo, and stimulated with recombinant mouse IFN-α2 (r-mIFN-α2) to induce ISG15 expression (**new Fig. 4D**). Notably, Dose-response experiments using r-mIFN-α2 stimulation (0, 10, and 20 ng/mL) efficiently induced the differentiation of CD8$^+$ T cells into an ISG15$^+$ CD8$^+$ T cell population (**new Fig. 4E**). Importantly, we further explored the functional consequences of this population. In the context of ICI-associated myocarditis, pathogenic T cells may induce apoptosis of cardiomyocytes[7, 8]. To directly validate the cytotoxicity of ISG15$^+$ CD8$^+$ T cells on cardiomyocytes, we conducted a T cell-mediated cell-killing assay by co-culturing ISG15$^+$CD8$^+$ T cells and HL-1 cardiomyocytes. ISG15$^+$CD8$^+$ T cells cocultured with cardiomyocytes at a 1:1 ratio (CD8:CM) exhibited significantly increased apoptosis (**new Fig. 4G**), elevated reactive oxygen species (ROS) production (**new Fig. 4H**), and enhanced expression of apoptosis markers and PUMA compared with controls (**new Fig. 4I**). Notably, these cytotoxic effects remained evident even at a lower ratio of 1:10 (**new Supplementary Fig. 6B-C**), indicating a robust cardiomyocyte-damaging capacity of ISG15$^+$CD8$^+$ T cells. To more directly investigate the role of ISG15, we knocked down ISG15 in CD8$^+$ T cells using siRNA. And we confirmed that the expression of ISG15 was significantly lower than those in the NC-siRNA groups (**new Fig. 4J**). Notably, ISG15-knockdown CD8$^+$ T cells failed to induce cardiomyocyte apoptosis, or ROS production, and were associated with markedly reduced expression of BAX and PUMA in cardiomyocytes (**new Fig. 4K-M**). These findings provide functional evidence supporting a direct pathogenic role of the ISG15$^+$ CD8$^+$ T-cell subset in ICI-associated myocarditis.

Collectively, although selective in vivo depletion of ISG15$^+$ CD8$^+$ T cells is currently not technically feasible, these complementary functional experiments provide mechanistic evidence supporting a direct pathogenic role of the ISG15$^+$ CD8$^+$ T-cell subset in promoting cardiomyocyte injury during myocarditis. We have revised the manuscript to present these findings more cautiously and to clearly acknowledge the limitations of the current experimental approaches.

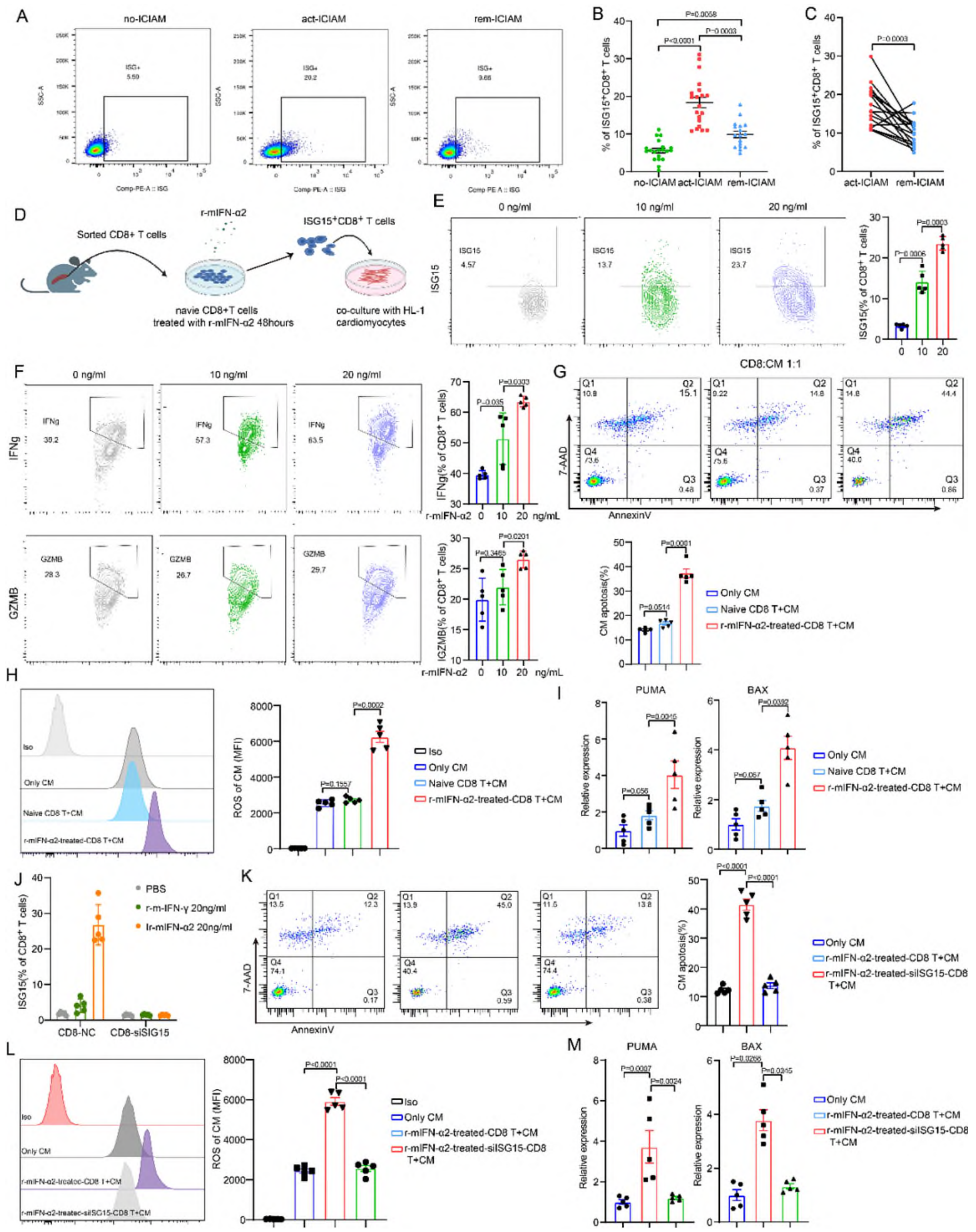

Fig. 4. ISG15⁺ CD8⁺ T cells induce cardiomyocyte apoptosis in co-culture assays.

A-B, Representative flow cytometry plots (A) and quantification (B) showing the frequency of $ISG15^{+}CD8^{+}$ T cells in the indicated groups.

C, Paired analysis showing dynamic changes in the proportion of $ISG15^{+}CD8^{+}$ T cells between act-ICIAM and rem-ICIAM samples.

D, Schematic workflow of the coculture assay between cardiomyocytes and $ISG15^{+}CD8^{+}$ T cells.

E, Flow cytometry analysis of ISG15 expression in $CD8^{+}$ T cells following stimulation with r-mIFN-α2 at the indicated concentrations (0, 10 and 20 ng/mL).

F, Flow cytometry analysis of IFNG and GZMB expression in $CD8^{+}$ T cells following

stimulation with r-mIFN-α2 at the indicated concentrations (0, 10 and 20 ng/mL).

G, Quantification of cardiomyocyte apoptosis after co-culture with $ISG15^+CD8^+$ T cells at a ratio of 1:1 ($ISG15^+CD8^+$ T:CM).

H, Quantitative analysis of reactive oxygen species (ROS) levels in cardiomyocytes cocultured with $ISG15^+CD8^+$ T cells at a ratio of 1:1.

I, Relative expression levels of BAX and PUMA in cardiomyocytes following coculture with $ISG15^+CD8^+$ T cells at a ratio of 1:1.

J, The specific siRNA was designed to interfere ISG15 expression in $CD8^+$ T cell and the suppressive efficacy was measured by flow cytometry assay.

K, Quantification of cardiomyocyte apoptosis after coculture with $CD8^+$ T cells following ISG15 siRNA-mediated knockdown.

L, Measurement and quantitative analysis of ROS levels in cardiomyocytes co-cultured with $CD8^+$ T cells after ISG15 siRNA transfection.

M, Relative expression of BAX and PUMA in cardiomyocytes co-cultured with $CD8^+$ T cells after ISG15 siRNA transfection.

Reference

1. Tong, Z., X. Yan, T. Chen, et al., Single-Cell Multi-Omics Identifies Specialized Cytotoxic and Migratory CD8(+) Effector T Cells in Acute Myocarditis. Circulation, 2025, 152(14): 1003-1022.
2. Zhang, L., K. Liu, X. Duan, et al., CXCL12/CXCR4 axis mediates CD8 (+) T cell overactivation in the progression of viral myocarditis. J Transl Med, 2025, 23(1): 399.
3. Santos-Zas, I., J. Lemarié, I. Zlatanova, et al., Cytotoxic CD8(+) T cells promote granzyme B-dependent adverse post-ischemic cardiac remodeling. Nat Commun, 2021, 12(1): 1483.
4. Martínez-Fleta, P., C. Pertusa, I. Fernández-Delgado, et al., ISG15 modulates inflammatory profiles and ability to activate CD8 + T cells in bone marrow-derived dendritic cells. Cell Mol Life Sci, 2025, 82(1): 362.
5. Chen, Y.L., A. Yousif, C.H. Chen, et al., Chronic ISG15 Exposure Accelerates CD8+ T-cell Dysfunction while Increasing PD-1 Blockade Sensitivity in Oral Squamous Cell Carcinoma. Cancer Immunol Res, 2025, 13(11): 1829-1844.
6. Iglesias-Guimarais, V., T. Ahrends, E. de Vries, et al., IFN-Stimulated Gene 15 Is an Alarmin that Boosts the CTL Response via an Innate, NK Cell-Dependent Route. J Immunol, 2020, 204(8): 2110-2121.
7. Huang, Y.V., Y. Sun, H. Chou, et al., Novel Therapeutic Approach Targeting CXCR3 to Treat Immunotherapy Myocarditis. Circ Res, 2025, 136(5): 473-490.
8. Axelrod, M.L., W.C. Meijers, E.M. Screever, et al., T cells specific for α-myosin drive immunotherapy-related myocarditis. Nature, 2022, 611(7937): 818-826.

7. In Figure 6A the legend does not match the colors in the Figure (i.e. group)

Author response: We thank you for pointing this out and sincerely apologize for the

inconsistency between the legend and the color coding in **Figure 6A**. We have corrected the figure to ensure that the legend now accurately matches the corresponding groups and colors, and we have also carefully checked the consistency of color assignments for other clinical variables throughout the manuscript. Please check it.

8. Figure 6J should be better explained, this remains

Author response: We thank for this helpful suggestion. The SHAP (Shapley Additive exPlanations) framework quantifies the contribution of each feature to the model's prediction by estimating the average change in prediction when the feature is included versus excluded across all possible feature combinations. In the revised manuscript, we have expanded the description of Fig. 6J to improve its interpretability. Specifically, we clarified that the SHAP summary (honeycomb) plot visualizes the magnitude and direction of individual feature contributions to the random forest model at the single-sample level, with features ranked by their mean absolute SHAP values. Each point represents an individual sample, and its horizontal position indicates whether a given feature increases or decreases the predicted probability of the outcome. Using this framework, we highlight that MMP1 exhibits the strongest overall contribution to the model prediction, followed by TRANCE and CXCL9, underscoring their dominant roles in shaping the model's decision-making process (**Fig. 6J, Supplementary Fig. 9E**). Corresponding explanatory text has been added to the Results section (Lines 516-522).

9. It remains unclear what is meant by L1-L5 in Figure7D (what are the different disease stages mentioned in the x-axis?

Author response: We thank the reviewer for pointing out this lack of clarity. In **Figure 7D**, L1-L5 refer to sample identifiers rather than distinct disease stages. To avoid misunderstanding, we have revised the figure and legend to explicitly define the groups: L1 and L2 correspond to control mice, whereas L3, L4, and L5 correspond to ICIAM mice. No additional disease staging was intended to be implied by the original labels.

10. It does not become clear how „exhaustion" is distinctly defined.

Author response: We thank you for raising this important point and agree that a clear definition of "exhaustion" is essential. In the revised manuscript, we categorized exhaustion-associated states using well-established marker sets reported in the previous study[1]. Terminally exhausted $CD8^+$ T cells were defined by the expression of markers

such as *PDCD1 (PD-1)*, *ENTPD1 (CD39)*, *HAVCR2 (TIM-3)*, and *TOX*, which are associated with sustained antigen stimulation and irreversible functional impairment. In contrast, progenitor exhausted $CD8^+$ T cells were defined by the co-expression of *PDCD1* together with memory- and self-renewal-associated markers including *TCF7*, *IL7R*, and *SELL*, consistent with a stem-like, differentiation-competent exhausted state. And we have now explicitly described these criteria in the Results sections (Lines 243, 257), please check it.

Reference

1. Thommen, D.S. and T.N. Schumacher, T Cell Dysfunction in Cancer. Cancer Cell, 2018, 33(4): 547-562.

Reviewer #3 myocarditis, cardio immune response (Remarks to the Author):

The manuscript of Zhang et al. addresses immune response during immune checkpoint inhibitor-associated myocarditis using scRNAseq on PBMC and plasma Olink/Luminex in human cohort with support of experiments in mouse model. Authors point to ISG15$^+$CD8$^+$ T cells as a key players in this condition, JAK-STAT pathway activation and CCR2-CCL2 axis as critical molecular mechanism. Furthermore, authors developed a 8-protein classifiers to identify ICIAM.

Overall, the manuscript is overloaded with bioinformatics analyses based on rather limited biological samples. Based of their nature, these analyses serve as hypothesis-generating tools. The actual validation of these hypotheses is mostly poor or inadequate. Furthermore, the experimental part often does not follow GLP principles.

Author response: We sincerely thank the reviewer for the careful and constructive evaluation of our manuscript. We appreciate the opportunity to clarify the rationale, validation strategy, and experimental rigor of our study, and we apologize if the initial presentation led to misunderstandings.

First, regarding the concern that the manuscript is "overloaded" with bioinformatic analyses, we would like to clarify that our study is intentionally designed as a comprehensive single-cell immune atlas of ICI-associated myocarditis. This design is consistent with recent high-impact studies on immune-related adverse events (irAEs), such as ICI-associated pneumonitis[1] and arthritis[2] published in *Nature Communications* and ICI-associated colitis[3] published in *Nature Medicine*. Given the rarity and acute clinical nature of ICI-associated myocarditis, a deep single-cell profiling represents a widely accepted and necessary approach to define disease-imprinted that cannot be captured by bulk assays[4]. Thus, the bioinformatic analyses serve as a structured discovery framework rather than an end point.

Second, while we agree that single-cell analyses are inherently hypothesis-generating, we respectfully disagree that validation in our study is inadequate. We implemented multi-layered orthogonal validation across independent modalities, including (i) plasma proteomics and development of an 8-protein diagnostic classifier addressing an unmet clinical need for non-invasive ICIAM detection, (ii) flow cytometric validation in an independent patient cohort, (iii) reanalysis of independent publicly available single-cell datasets, and (iv) functional experiments using ex vivo cardiomyocyte co-culture systems and established mouse models. Together, these approaches move beyond descriptive bioinformatics and provide convergent evidence supporting the pathogenic relevance of key immune populations, including ISG15$^+$ CD8$^+$ T cells.

Third, regarding adherence to GLP principles, we would like to clarify that all experiments were conducted under standardized, protocol-driven conditions, with predefined procedures for animal handling, dosing, sample collection, and data reporting. Variability in sample numbers across specific readouts reflects biological constraints inherent to fulminant myocarditis models[4, 5], such as premature mortality preventing certain measurements, rather than deviations from GLP or selective reporting. Similar variability has been explicitly reported in recent high-impact studies using comparable ICI-myocarditis models[6-9]. To further enhance transparency, we have revised the Methods to clarify experimental workflows and explicitly describe randomization, blinding where applicable, and inclusion/exclusion criteria.

In summary, our study follows a discovery-to-translation paradigm that is well established in irAE research: deep multi-omic profiling to define disease-associated immune states, followed by orthogonal validation and clinically oriented translation. We believe this integrated approach is scientifically justified and appropriate for advancing understanding and diagnosis of rare, life-threatening conditions such as ICI-associated myocarditis. All revised portions are marked in **RED** in the manuscript. Below, we provide a detailed point-by-point response to each comment.

Reference

1. Lin, X., C. Li, J. Deng, et al., Uncovering the immune mechanisms underlying the emergence of immunotherapy-induced pneumonitis in lung cancer patients. Nat Commun, 2025, 16(1): 11645.
2. Zhou, Z., X. Zhou, X. Jiang, et al., Single-cell profiling identifies IL1B(hi) macrophages associated with inflammation in PD-1 inhibitor-induced inflammatory arthritis. Nat Commun, 2024, 15(1): 2107.
3. Thomas, M.F., K. Slowikowski, K. Manakongtreecheep, et al., Single-cell transcriptomic analyses reveal distinct immune cell contributions to epithelial barrier dysfunction in checkpoint inhibitor colitis. Nat Med, 2024, 30(5): 1349-1362.
4. Blum, S.M., D.A. Zlotoff, N.P. Smith, et al., Immune responses in checkpoint myocarditis across heart, blood and tumour. Nature, 2024, 636(8041): 215-223.
5. Wei, S.C., W.C. Meijers, M.L. Axelrod, et al., A Genetic Mouse Model Recapitulates Immune Checkpoint Inhibitor-Associated Myocarditis and Supports a Mechanism-Based Therapeutic Intervention. Cancer Discov, 2021, 11(3): 614-625.
6. Axelrod, M.L., W.C. Meijers, E.M. Screever, et al., T cells specific for α-myosin drive immunotherapy-related myocarditis. Nature, 2022, 611(7937): 818-826.
7. Ma, P., J. Liu, J. Qin, et al., Expansion of Pathogenic Cardiac Macrophages in Immune Checkpoint Inhibitor Myocarditis. Circulation, 2024, 149(1): 48-66.
8. Zhang, Y., C. Sun, Y. Li, et al., Hormonal therapies up-regulate MANF and overcome female susceptibility to immune checkpoint inhibitor myocarditis. Sci Transl Med, 2022, 14(669): eabo1981.
9. Xue, G., X. Li, M. Kalim, et al., Clinical drug screening reveals clofazimine potentiates

the efficacy while reducing the toxicity of anti-PD-1 and CTLA-4 immunotherapy. Cancer Cell, 2024, 42(5): 780-796.e6.

Specific comments:

1) Clinical cohorts. Discovery cohort show no comparison between analysed groups (no-ICIAM, act-ICIAM, and rem-ICIAM) for clinical parameters. Validation cohort is impressive, but no experiment is performed on the whole cohort! For example, flow cytometry analysis or ELISAs show n=22 (out of 270). It is unclear how patients were preselected.

Author response: We thank the reviewer for this thoughtful comment and the opportunity to clarify the design and interpretation of our clinical cohorts.

First, regarding the discovery cohort, in fact, comparisons of clinical parameters across the no-ICIAM, act-ICIAM, and rem-ICIAM groups were already presented in **Fig. 2E** in the form of a heatmap. Heatmap-based visualization is a widely accepted approach for summarizing and comparing multidimensional clinical variables and has been extensively used in high-impact journals[1, 2]. The discovery cohort corresponds to our in-house scRNA-seq cohort. To further improve transparency and address your concern, we have now added comprehensive comparisons of baseline demographics, clinical characteristics, and laboratory parameters across the three groups, with the full numerical results provided in **Supplementary Table 1** in the revised manuscript.

Second, with respect to the validation cohort, we would like to clarify that there was no patient preselection for downstream assays. ICI-associated myocarditis is a rare but highly lethal immune-related adverse event, with a reported incidence of approximately 0.09-2.4%, which makes prospective biospecimen collection exceptionally challenging and renders each well-annotated sample extremely valuable[3, 4]. As a result, not all assays could be performed for every patient due to limited sample availability, a constraint that is common and widely acknowledged in ICI-related adverse events research[5]. To our knowledge, our study represents one of the largest and most comprehensive patient-based investigations of ICIAM to date, both in terms of patient number and multi-omic depth. Our cohort integrates peripheral blood and myocardial tissue analyses and combines single-cell transcriptomics, plasma proteomics, and flow cytometry, providing convergent evidence across multiple experimental layers. Notably, the overall sample size and analytical breadth are comparable to, or exceed, those reported in recent high-impact ICIAM studies, including a recent study published in *Nature*[5]. Importantly, the sample size in our study is also in line with, or larger than, those used in recent single-cell studies of ICI-related adverse events more broadly. For example, A recent study published in *Nature*

Medicine investigated ICI-associated colitis in 13 patients using scRNA-seq, emphasizing that for rare immune-related adverse events, biological insights are frequently derived from in-depth, multi-layered profiling rather than large cohort size alone[6]. Similarly, on November 6, 2024, multiple research teams from Massachusetts General Hospital, the Massachusetts Institute of Technology, and Harvard Medical School reported an integrated analysis of checkpoint inhibitor–associated myocarditis across heart, blood, and tumor tissues in *Nature*[5]. Importantly, these studies underscore that not all assays can be uniformly applied to every patient because of limited biospecimen availability, an inherent and widely recognized challenge in studies of rare immune-related adverse events, including ICI-associated myocarditis. However, this limitation did not compromise the robustness or validity of their conclusions.

Thirdly, we would like to emphasize that patients were not preselected for any specific downstream assays in this study. As illustrated in the study overview (**Fig. 1A**), patients were assigned to assay-specific cohorts solely based on biospecimen availability, including a single-cell transcriptomic cohort comprising an in-house scRNA-seq cohort (n=19, **Supplementary Table 1**) and a publicly available scRNA-seq cohort (n=11), a flow cytometry cohort (n=57, **Supplementary Table 2**), and a Luminex assay cohort (n=68, **Supplementary Table 3**). Importantly, this allocation was independent of clinical outcome or immunological phenotype. Within each assay-specific cohort, demographic characteristics, clinical features, and immunotherapy regimens were comparable between groups, thereby minimizing potential confounding arising from cohort composition. This design reflects a widely accepted methodological framework in irAE research, where the rarity of the condition and the logistical constraints of timely sample acquisition preclude the uniform application of all experimental assays across an entire clinical cohort. Rather than introducing bias, this approach allows complementary, multi-omic interrogation across distinct but overlapping patient subsets, providing convergent biological evidence from independent experimental layers. For rare and clinically acute conditions such as ICI-associated myocarditis, such depth-oriented, multi-layered profiling is recognized as a robust and informative strategy, even when individual assays cannot be performed for every patient[7, 8].

To avoid any potential misunderstanding, we have revised the **Fig. 1A** to more clearly depict patient inclusion, biospecimen availability, and assay-specific allocation. We believe these clarifications and additions adequately address your concerns and further strengthen the transparency and rigor of the study.

[editorial note: third party material redacted]

Fig. 1. A, workflow showing the collection and processing of samples for scRNA-seq, flow cytometry, Olink proteomics, and multiplex cytokine assays.

Reference

1. Lin, X., C. Li, J. Deng, et al., Uncovering the immune mechanisms underlying the emergence of immunotherapy-induced pneumonitis in lung cancer patients. Nat Commun, 2025, 16(1): 11645.
2. Dimitriou, F., P.F. Cheng, A. Saltari, et al., A targetable type III immune response with increase of IL-17A expressing CD4(+) T cells is associated with immunotherapy-induced toxicity in melanoma. Nat Cancer, 2024, 5(9): 1390-1408.
3. Bonaca, M.P., B.A. Olenchock, J.E. Salem, et al., Myocarditis in the Setting of Cancer Therapeutics: Proposed Case Definitions for Emerging Clinical Syndromes in Cardio-Oncology. Circulation, 2019, 140(2): 80-91.
4. Moslehi, J., A.H. Lichtman, A.H. Sharpe, et al., Immune checkpoint inhibitor-associated myocarditis: manifestations and mechanisms. J Clin Invest, 2021, 131(5).
5. Blum, S.M., D.A. Zlotoff, N.P. Smith, et al., Immune responses in checkpoint myocarditis across heart, blood and tumour. Nature, 2024, 636(8041): 215-223.
6. Thomas, M.F., K. Slowikowski, K. Manakongtreecheep, et al., Single-cell transcriptomic analyses reveal distinct immune cell contributions to epithelial barrier dysfunction in checkpoint inhibitor colitis. Nat Med, 2024, 30(5): 1349-1362.
7. Zhou, Z., X. Zhou, X. Jiang, et al., Single-cell profiling identifies IL1B(hi) macrophages associated with inflammation in PD-1 inhibitor-induced inflammatory arthritis. Nat Commun, 2024, 15(1): 2107.
8. Bukhari, S., B.S. Henick, R.J. Winchester, et al., Single-cell RNA sequencing reveals distinct T cell populations in immune-related adverse events of checkpoint inhibitors. Cell Rep Med, 2023, 4(1): 100868.

2) Mouse studies. Presented data show no myocarditis in this model, which is not surprising as BL6 mice are myocarditis-resistant. CD45 looks unspecific, with no

inflammatory aggregates typical for myocarditis (on H/E or CD45 IHC). It is very concerning that experimental data for mouse studies vary between n=3-5. For example, in Fig. 8 in anti-PD1 PBS group for serum measurement n=5, while for EF n=3. Furthermore, Method section describes (briefly) only 1 out of 3 mouse experiments.

Author response: We thank the reviewer for this careful and constructive feedback.

First, we acknowledge that C57BL/6 mice are relatively resistant to classical experimental autoimmune myocarditis, such as cardiac myosin-induced models, in the absence of strong immune priming. However, ICI-related myocarditis represents a distinct immune-mediated entity, and myocardial injury or myocarditis-like phenotypes have been reproducibly reported in C57BL/6 mice under appropriate ICI-based regimens. Notably, a recent study published in Nature Communications successfully demonstrated anti-PD-1-associated cardiotoxicity in C57BL/6 mice, using repeated anti-PD-1 administration alone[1]. This work provides direct evidence that C57BL/6 mice are suitable for modeling ICI-associated cardiac injury when immune activation is sufficiently engaged. In addition, anti-PD-1-induced cardiac myocarditis has also been reported in C57BL/6 mice in earlier studies[2], further supporting the feasibility of this strain for ICI myocarditis research. On the other hand, more fulminant autoimmune myocarditis models on a C57BL/6 background, such as those reported in Cancer Cell (2024), typically require repeated cardiac troponin I (cTnI) peptide immunization in complete Freund's adjuvant combined with intensive dual PD-1/CTLA-4 blockade[3]. These models are designed to maximize autoimmune severity and may overrepresent extreme phenotypes compared with the clinical spectrum of ICI-related myocarditis. In our study, we adopted a well-established intermediate modeling strategy that has been validated in our previous studies[4-6].You can carefully check it. Specifically, mice were immunized with cTnI peptide on days 0 and 7, followed by repeated anti-PD-1 administration from day 7 onward. This approach is intended to break peripheral tolerance and unmask PD-1-dependent cardiac autoimmunity, thereby modeling the immunopathogenesis of ICI-related myocarditis rather than classical autoimmune myocarditis. Importantly, although we did not aim to induce uniformly fulminant myocarditis, our model showed clear evidence of cardiac immune injury and activation. this combinatorial approach elicited reproducible myocardial inflammation marked by predominant infiltration of CD8+ T cells and macrophages, along with significant myocardial injury by day 7 post-treatment (**Fig. 1R**), thereby closely mimicking the pathological features observed in human ICIAM.

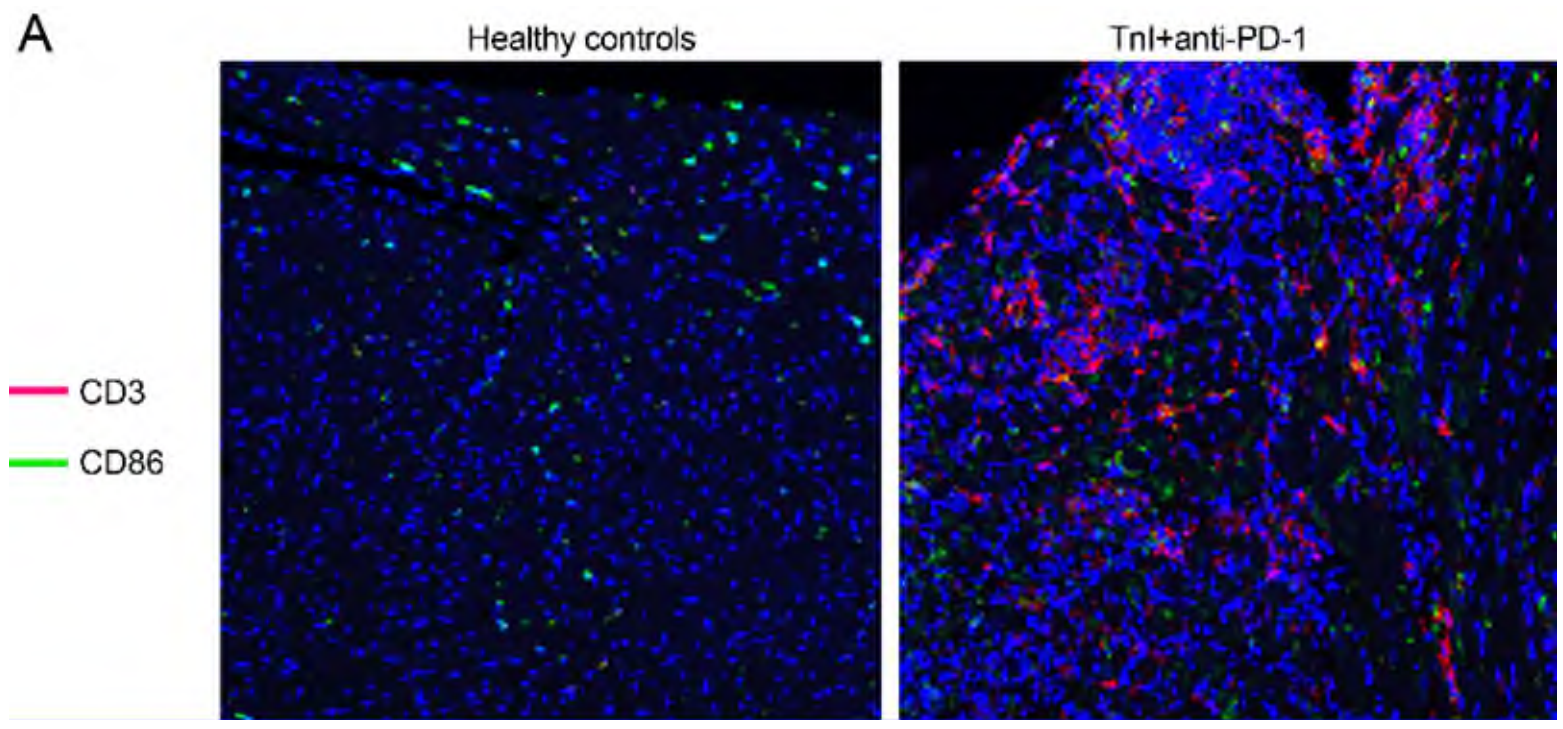

Fig. 1R. Representative immunofluorescence staining of T cells and macrophages in the cardiac tissues from each indicated experimental group.

Importantly, after carefully considering the reviewer's concern, we re-evaluated myocardial pathology using H&E staining, and Masson's staining, as you suggested. These analyses revealed clear and significant myocarditis phenotypes (**Fig. 5I-J, Fig. 8F-G**).

Second, we appreciate the reviewer's concern that CD45 staining appeared non-specific and that typical inflammatory aggregates were not evident. In the revised manuscript (**Fig. 5I-J, Fig. 8C-G**), we strengthened myocarditis validation using multiple orthogonal, widely accepted endpoints[7-9], including: 1) H&E staining with representative images at multiple magnifications and standardized inflammation scoring; 2) Masson's staining; 3) cardiac injury biomarkers: serum cTnT measurements.

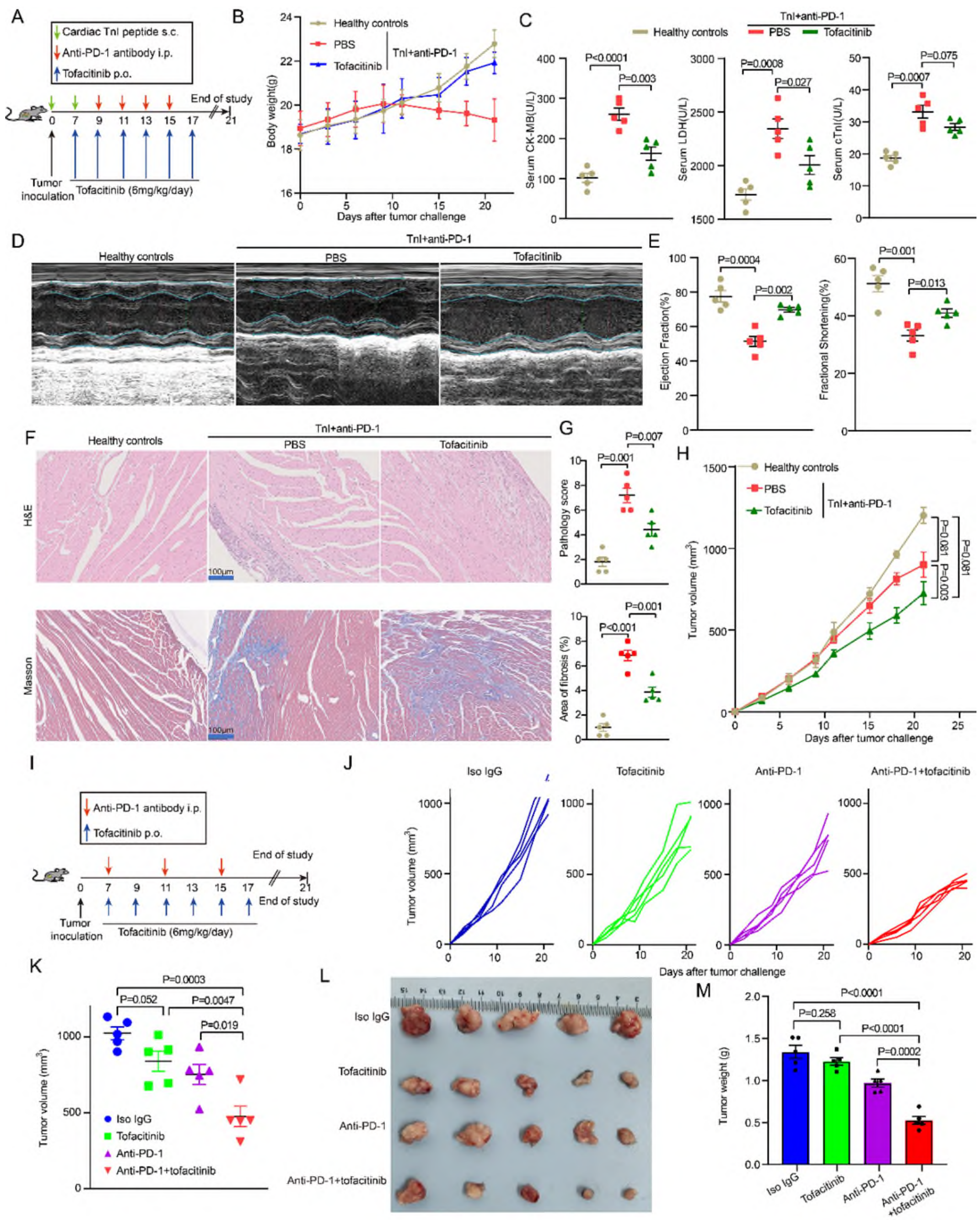

Fig. 8. JAK-STAT inhibition ameliorated cardiac inflammation and restored anti-PD-1 response in ICIAM model.

A, Treatment diagram for MC38 tumor-bearing mice induced with ICIAM by cardiac Tnl peptide and anti-PD-1 injection.

B, Body weight surveillance during the experiment described above (n =5).

C, Serum levels of CK-MB, LDH, and cTnI in mice (n = 5).

D-E, Representative photographs of echocardiography (D) and quantification of cardiac properties and output (E) in echocardiography test.

F-G, Representative images of H&E staining and Masson staining in mouse hearts of each group and their statistical analysis (n = 5).

H, Tumor growth curves of MC38 tumor-bearing mice (n = 5).
I, Schematic of treatment protocol for MC38 tumor-bearing mice receiving tofacitinib and anti-PD-1 therapy without myocarditis induction.
J, Individual tumor growth curves of mice (n = 5).
K,Tumor volumes from the tumor-bearing mice (n = 5).
L, Representative images of tumor tissues at the end of experiment (n = 5).
M, Tumor weights of MC38-bearing mice in the treatment groups (n = 5).

Third, for sample size consistency, we would like to clarify that all animal experiments in this study were conducted in strict accordance with GLP-compliant experimental procedures, including predefined protocols for animal handling, treatment administration, sample collection, and data recording. The observed differences in sample size between specific readouts (e.g., serum biomarkers, echocardiographic parameters in **Fig. 8G-E**) do not reflect noncompliance with GLP principles, but rather arise from the biological characteristics of the myocarditis model itself. In the anti-PD-1-treated PBS group, a subset of mice developed rapidly progressive and fatal myocarditis, leading to premature death before scheduled echocardiographic assessment could be performed. Consequently, while plasma samples collected prior to death remained available for serum analyses (n=5), echocardiographic measurements such as ejection fraction could only be obtained from surviving animals (n=3). This phenomenon is well recognized in fulminant myocarditis models and has been reported in prior high-impact studies using similar ICI-associated myocarditis models, including recent work[4] published in *Cancer Cell*. To further address your concern, in the revised study, we repeated all the major animal experiments and standardized group sizes to n =5 per group for key readouts wherever feasible, with predefined inclusion and exclusion criteria explicitly described in the Methods.

Finally, for the description of the animal experiments, in fact, all three mouse experiments were described in the Methods section in the original submission, including the disease induction protocols, treatment regimens, dosing schedules, and experimental timelines. However, we acknowledge that the presentation may not have been sufficiently clear or clearly separated, which could have given the impression that only one experiment was described in detail. To improve clarity and readability, we have now reorganized the Methods section and explicitly separated the three mouse experiments into individual subsections, each with a clear heading. For each experiment, we now provide a detailed description of the mouse model, induction method, treatment strategy, dosing, and outcome assessment Methods section (Line 748-778). We believe that this revised organization more clearly reflects the full scope of the in vivo work and addresses the reviewer’s concern.

Reference

1. Huang, R., Z.Y. Shen, D. Huang, et al., Microbiota-indole-3-propionic acid-heart axis mediates the protection of leflunomide against αPD1-induced cardiotoxicity in mice. Nat Commun, 2025, 16(1): 2651.
2. Xia, W., C. Zou, H. Chen, et al., Immune checkpoint inhibitor induces cardiac injury through polarizing macrophages via modulating microRNA-34a/Kruppel-like factor 4 signaling. Cell Death Dis, 2020, 11(7): 575.
3. Xue, G., X. Li, M. Kalim, et al., Clinical drug screening reveals clofazimine potentiates the efficacy while reducing the toxicity of anti-PD-1 and CTLA-4 immunotherapy. Cancer Cell, 2024, 42(5): 780-796.e6.
4. He, X., J. Zhang, Y. Zhang, et al., L-Kynurenine regulates immune response in ICIs-associated myocarditis via JAK/STAT pathway. Int Immunopharmacol, 2025, 156: 114676.
5. Wang, X., J. Chen, Y. Shen, et al., Baricitinib protects ICIs-related myocarditis by targeting JAK1/STAT3 to regulate Macrophage polarization. Cytokine, 2024, 179: 156620.
6. Zhang, H., J. Lin, Y. Shen, et al., Protective Effect of Crocin on Immune Checkpoint Inhibitors-Related Myocarditis Through Inhibiting NLRP3 Mediated Pyroptosis in Cardiomyocytes via NF-κB Pathway. J Inflamm Res, 2022, 15: 1653-1666.
7. Wei, S.C., W.C. Meijers, M.L. Axelrod, et al., A Genetic Mouse Model Recapitulates Immune Checkpoint Inhibitor-Associated Myocarditis and Supports a Mechanism-Based Therapeutic Intervention. Cancer Discov, 2021, 11(3): 614-625.
8. Yu, J., B. Long, Z. Li, et al., Central memory CD4+ T cells play a protective role against immune checkpoint inhibitor-associated myocarditis. Cardiovasc Res, 2024, 120(12): 1442-1455.
9. Axelrod, M.L., W.C. Meijers, E.M. Screever, et al., T cells specific for α-myosin drive immunotherapy-related myocarditis. Nature, 2022, 611(7937): 818-826.

3) Validation in mouse study is superficial. Even if experiments were performed in the right model, blockade of all $CD8^+$ cells can not validate pathogenic role of $ISG15^+CD8^+$ T subset as suggested in this manuscript.

Author response: We thank your insightful comment and we fully agree with the central point. We also apologize that the original wording may have caused the impression that we were claiming subset-specific causality based on bulk $CD8^+$ depletion. In the revised manuscript, we have removed the corresponding statements. Importantly, we did attempt to directly and specifically target $ISG15^+CD8^+$ T cells in vivo. However, this was not feasible due to a practical technical limitation: to our knowledge, there is currently no commercially available, in vivo-validated ISG15-neutralizing antibody that would enable specific depletion of $ISG15^+CD8^+$ T cells in the mouse model. We respectfully ask the reviewer to understand that this limitation reflects the current tool availability in the field rather than a lack of effort. Once

commercially available antibodies or small-molecule inhibitors targeting ISG15 become established and validated for in vivo use, we will further validate and extend the findings of the current study. Given this constraint, we strengthened our validation using an ex vivo functional approache[1-3] that more directly interrogate the pathogenic potential of ISG15^{+}CD8^{+} T cells as follows:

Previous studies have established that ISG15 is a canonical interferon-stimulated gene that is robustly upregulated downstream of type I interferon signaling, and can be induced by IFN-α2[4-6]. Thus, we isolated murine splenic CD8^{+} T cells and cocultured ex vivo, and stimulated with recombinant mouse IFN-α2 (r-mIFN-α2) to induce ISG15 expression (**new Fig. 4D**). Notably, Dose-response experiments using r-mIFN-α2 stimulation (0, 10, and 20 ng/mL) efficiently induced the differentiation of CD8^{+} T cells into an ISG15^{+} CD8^{+} T cell population (**new Fig. 4E**). Importantly, we further explored the functional consequences of this population. In the context of ICI-associated myocarditis, pathogenic T cells may induce apoptosis of cardiomyocytes[7, 8]. To directly validate the cytotoxicity of ISG15^{+} CD8^{+} T cells on cardiomyocytes, we conducted a T cell-mediated cell-killing assay by co-culturing ISG15^{+}CD8^{+} T cells and HL-1 cardiomyocytes. ISG15^{+}CD8^{+} T cells cocultured with cardiomyocytes at a 1:1 ratio (CD8:CM) exhibited significantly increased apoptosis (**new Fig. 4G**), elevated reactive oxygen species (ROS) production(**new Fig. 4H**), and enhanced expression of apoptosis markers and PUMA compared with controls (**new Fig. 4I**). Notably, these cytotoxic effects remained evident even at a lower ratio of 1:10 (new **Supplementary Fig. 6B-C**), indicating a robust cardiomyocyte-damaging capacity of ISG15^{+}CD8^{+} T cells. To more directly investigate the role of ISG15, we knocked down ISG15 in CD8^{+} T cells using siRNA. And we confirmed that the expression of ISG15 was significantly lower than those in the NC-siRNA groups (**new Fig. 4J**). Notably, ISG15-knockdown CD8^{+} T cells failed to induce cardiomyocyte apoptosis, or ROS production, and were associated with markedly reduced expression of BAX and PUMA in cardiomyocytes (**new Fig. 4K-M**). These findings provide functional evidence supporting a direct pathogenic role of the ISG15^{+} CD8^{+} T-cell subset in ICI-associated myocarditis.

Collectively, although selective in vivo depletion of ISG15^{+} CD8^{+} T cells is currently not technically feasible, these complementary functional experiments provide mechanistic evidence supporting a direct pathogenic role of the ISG15^{+} CD8^{+} T-cell subset in promoting cardiomyocyte injury during myocarditis. We have revised the manuscript to present these findings more cautiously and to clearly acknowledge the limitations of the current experimental approaches.

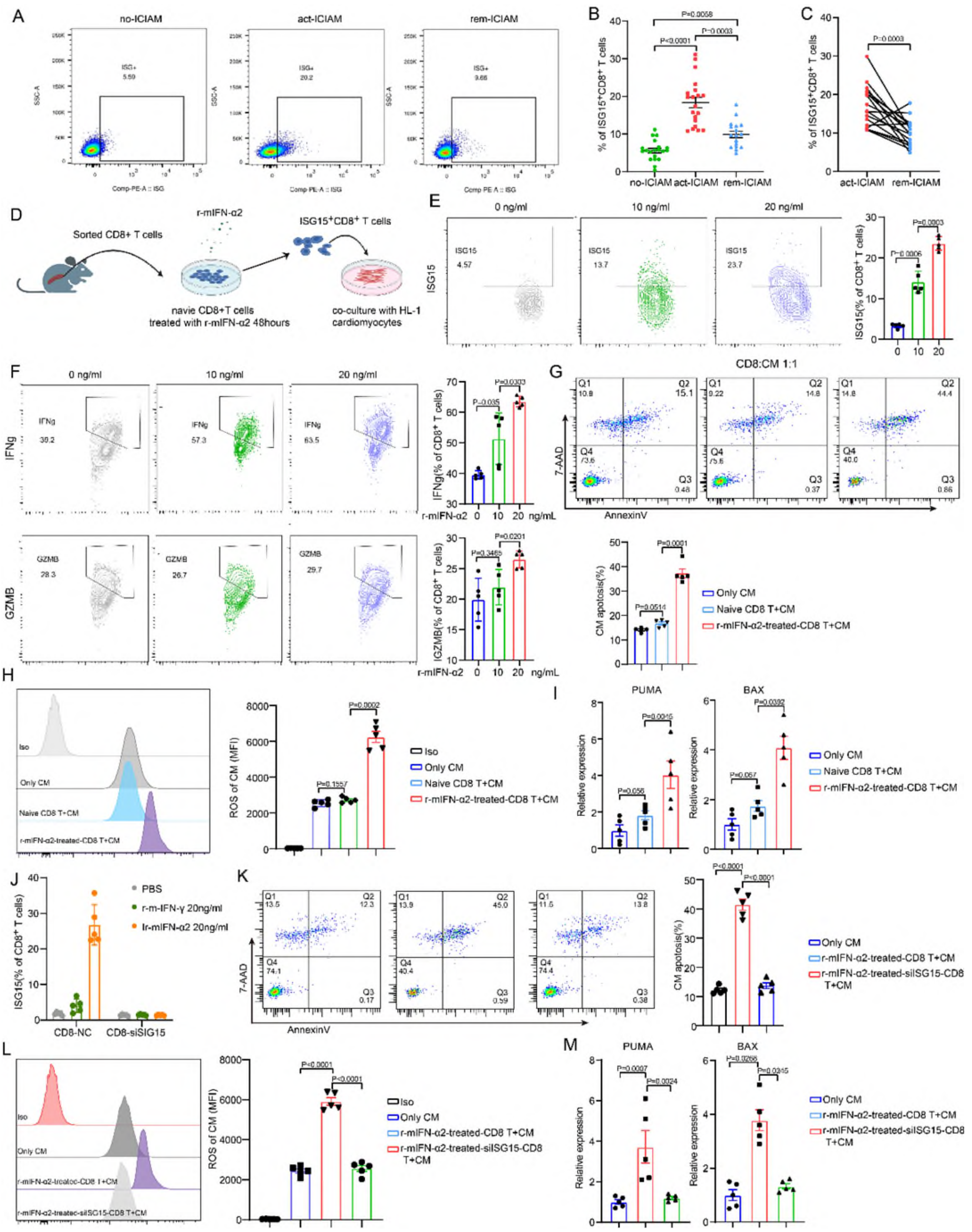

Fig. 4. ISG15⁺ CD8⁺ T cells induce cardiomyocyte apoptosis in co-culture assays

A-B, Representative flow cytometry plots (A) and quantification (B) showing the frequency of ISG15$^+$CD8$^+$ T cells in the indicated groups.

C, Paired analysis showing dynamic changes in the proportion of ISG15$^+$CD8$^+$ T cells between act-ICIAM and rem-ICIAM samples.

D, Schematic workflow of the coculture assay between cardiomyocytes and ISG15$^+$CD8$^+$ T cells.

E, Flow cytometry analysis of ISG15 expression in CD8$^+$ T cells following stimulation with r-mIFN-α2 at the indicated concentrations (0, 10 and 20 ng/mL).

F, Flow cytometry analysis of IFNG and GZMB expression in CD8$^+$ T cells following

stimulation with r-mIFN-α2 at the indicated concentrations (0, 10 and 20 ng/mL).
G, Quantification of cardiomyocyte apoptosis after co-culture with ISG15^{+}CD8^{+} T cells at a ratio of 1:1 (ISG15^{+}CD8^{+} T:CM).
H, Quantitative analysis of reactive oxygen species (ROS) levels in cardiomyocytes cocultured with ISG15^{+}CD8^{+} T cells at a ratio of 1:1.
I, Relative expression levels of BAX and PUMA in cardiomyocytes following coculture with ISG15^{+}CD8^{+} T cells at a ratio of 1:1.
J, The specific siRNA was designed to interfere ISG15 expression in CD8^{+} T cell and the suppressive efficacy was measured by flow cytometry assay.
K, Quantification of cardiomyocyte apoptosis after coculture with CD8^{+} T cells following ISG15 siRNA-mediated knockdown.
L, Measurement and quantitative analysis of ROS levels in cardiomyocytes co-cultured with CD8^{+} T cells after ISG15 siRNA transfection.
M, Relative expression of BAX and PUMA in cardiomyocytes co-cultured with CD8^{+} T cells after ISG15 siRNA transfection.

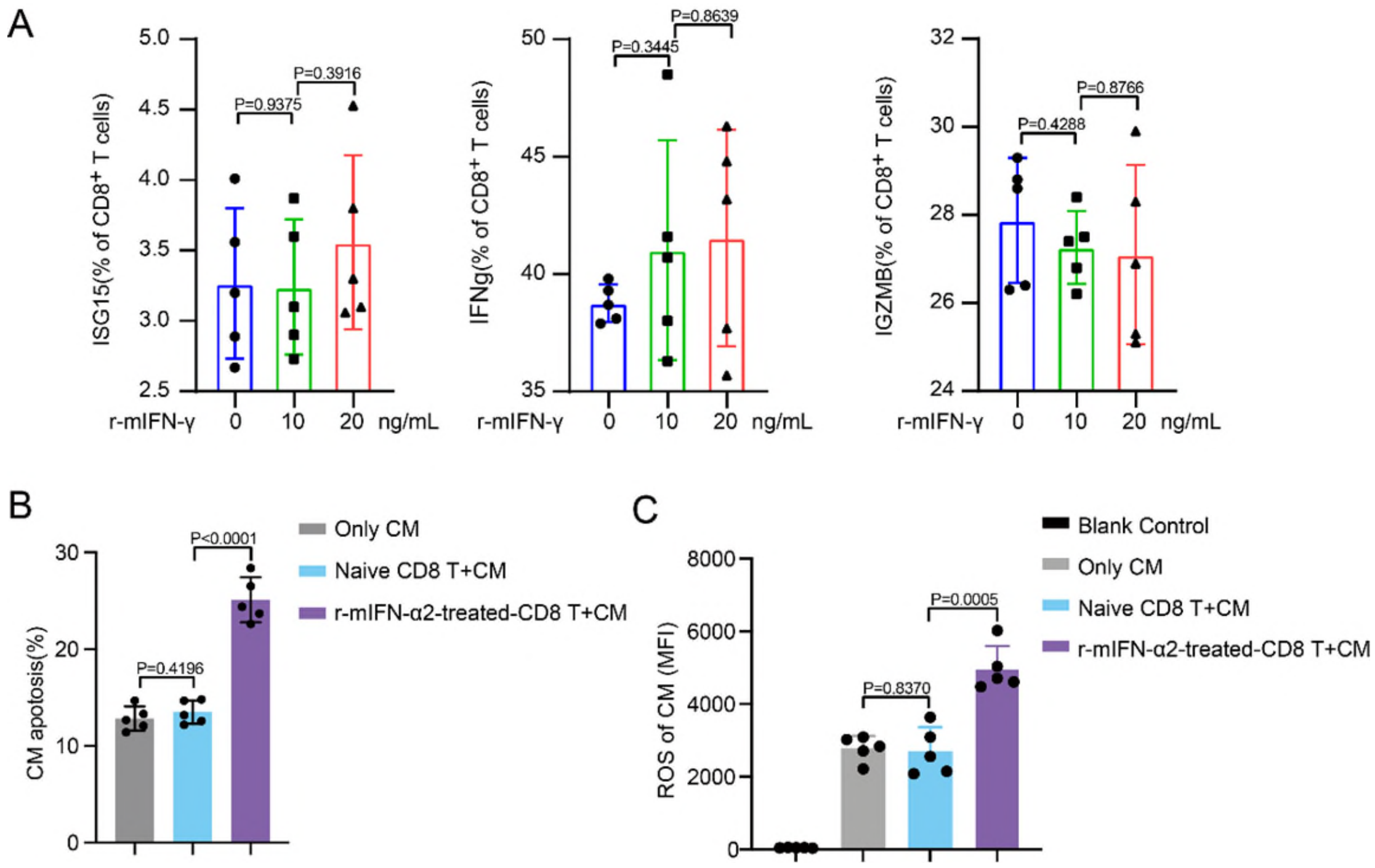

Supplementary Fig. 6. ISG15 induction and cytotoxic effects of ISG15^{+}CD8^{+} T cells on cardiomyocyte at a low ratio.
A, Flow cytometry analysis of ISG15 expression in murine CD8^{+} T cells following stimulation with recombinant type II interferon (r-mIFN-γ) at the indicated concentrations (0,10, and 20 ng/mL).
B, Quantification of cardiomyocyte apoptosis after coculture with ISG15^{+}CD8^{+} T cells at a ratio of 1:10.
C, Quantitative analysis of reactive oxygen species (ROS) levels in cardiomyocytes

cocultured with $ISG15^{+}CD8^{+}$ T cells at a ratio of 1:10.

Reference

1. Tong, Z., X. Yan, T. Chen, et al., Single-Cell Multi-Omics Identifies Specialized Cytotoxic and Migratory CD8(+) Effector T Cells in Acute Myocarditis. Circulation, 2025, 152(14): 1003-1022.
2. Zhang, L., K. Liu, X. Duan, et al., CXCL12/CXCR4 axis mediates CD8 (+) T cell overactivation in the progression of viral myocarditis. J Transl Med, 2025, 23(1): 399.
3. Santos-Zas, I., J. Lemarié, I. Zlatanova, et al., Cytotoxic CD8(+) T cells promote granzyme B-dependent adverse post-ischemic cardiac remodeling. Nat Commun, 2021, 12(1): 1483.
4. Martínez-Fleta, P., C. Pertusa, I. Fernández-Delgado, et al., ISG15 modulates inflammatory profiles and ability to activate CD8 + T cells in bone marrow-derived dendritic cells. Cell Mol Life Sci, 2025, 82(1): 362.
5. Chen, Y.L., A. Yousif, C.H. Chen, et al., Chronic ISG15 Exposure Accelerates CD8+ T-cell Dysfunction while Increasing PD-1 Blockade Sensitivity in Oral Squamous Cell Carcinoma. Cancer Immunol Res, 2025, 13(11): 1829-1844.
6. Iglesias-Guimarais, V., T. Ahrends, E. de Vries, et al., IFN-Stimulated Gene 15 Is an Alarmin that Boosts the CTL Response via an Innate, NK Cell-Dependent Route. J Immunol, 2020, 204(8): 2110-2121.
7. Huang, Y.V., Y. Sun, H. Chou, et al., Novel Therapeutic Approach Targeting CXCR3 to Treat Immunotherapy Myocarditis. Circ Res, 2025, 136(5): 473-490.
8. Axelrod, M.L., W.C. Meijers, E.M. Screever, et al., T cells specific for α-myosin drive immunotherapy-related myocarditis. Nature, 2022, 611(7937): 818-826.

4) Along the same line, analysis of transient appearance of ISG15+CD8+ T cells can be directly measured in the blood of the same patients during and post myocarditis. Instead, authors used monocle pseudotime trajectory analysis with validation of total CD8+ in mouse model which is superficial.

Author response: We highly acknowledge your recommendation. We fully agree that the most direct and biologically rigorous approach to demonstrate the transient emergence of $ISG15^{+}CD8^{+}$ T cells would be longitudinal analysis of peripheral blood from the same patients during during and post myocarditis.

To directly address your concern, we further performed longitudinal validation using flow cytometry and expanded the sample size to include 16 paired peripheral blood samples collected from the same patients during the acute phase of ICIAM and after clinical remission. $ISG15^{+}CD8^{+}$ T cells were directly quantified by flow cytometric analysis in each paired sample. Importantly, this within-patient longitudinal analysis revealed a significant reduction in the frequency of $ISG15^{+}CD8^{+}$ T cells in the

post myocarditis compared with the acute myocarditis phase (**new Fig. 4C**). These data provide direct evidence that ISG15^{+}CD8^{+} T cells expand transiently during active disease and decline following clinical resolution. We have incorporated these results into the revised manuscript and revised the text to emphasize experimentally observed temporal dynamics. Accordingly, the pseudotime analysis is now presented in the **Supplementary Fig. 4**.

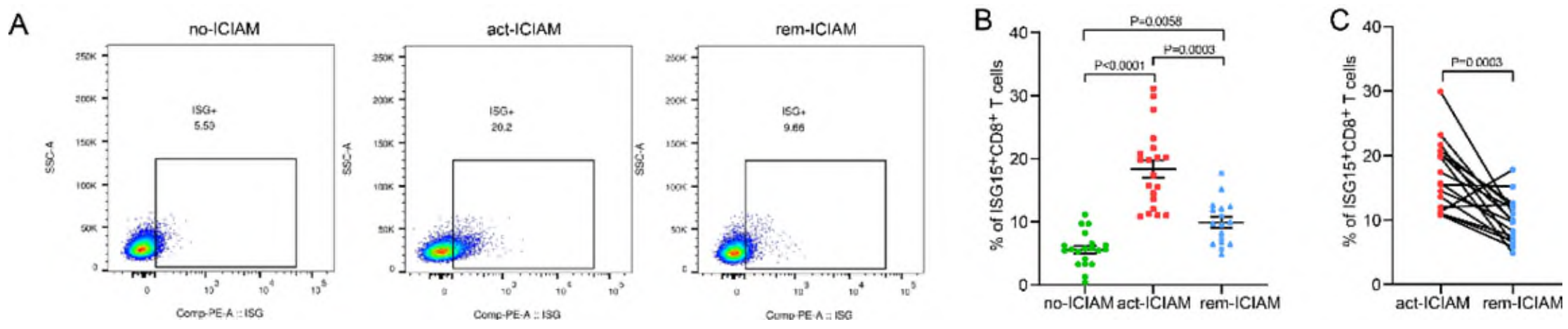

Fig. 4. A-B, Representative flow cytometry plots (A) and quantification (B) showing the frequency of ISG15^{+}CD8^{+} T cells in the indicated groups.
C, Paired analysis showing dynamic changes in the proportion of ISG15^{+}CD8^{+} T cells between act-ICIAM and rem-ICIAM samples.

5) In depth analysis of scRNAseq data is nearly exclusively shown for ISG15+CD8+ T subset. The question arises, whether the identified mechanisms are the same in other cell populations in ICIAM patients?

Author response: We have carefully considered the comments and made corresponding revisions accordingly. We agree that it is essential to evaluate whether the implicated mechanisms are shared across other immune populations in ICI-associated myocarditis. We have carefully considered this point and provide the following clarifications:

First, we performed an unbiased global analysis of PBMC scRNA-seq data and identified six major immune cell types, namely NK cells, CD8^{+} T cells, CD4^{+} T cells, B cells, monocytes, and DCs. We compared immune-cell composition across the no-ICIAM, act-ICIAM, and rem-ICIAM groups and found that, compared with no-ICIAM, act-ICIAM patients exhibited a significant increase in the proportion of CD8^{+} T cells and significant decreases in the proportion of monocytes (**Fig. 1E**). These global results provide the context that CD8^{+} T cells represent the most prominently expanded compartment during active ICIAM in our dataset.

Second, to further investigate functional alterations across major cell types, we used hallmark gene sets to to compare pathway activities between the no-ICIAM and act-ICIAM groups. Intriguingly, CD8^{+} T cells and monocytes showed upregulation of a wide variety of pathway activities, including different aspects of immunology,

metabolism, signalling, and proliferation (**new Fig. 1F**), implying that distinct anatomical contexts may preferentially remodel these cells and induce specific functional states. Furthermore, inflammatory-response and interferon-related pathways were upregulated in $CD8^+$ T cells, and monocytes showed greater enrichment of metabolism-associated pathways compared with the no-ICIAM group (**new Fig. 1F**). In addition, $CD4^+$ T cells, B cells, NK cells, and DCs displayed minimal changes in pathway activity. Accordingly, subsequent analyses were focused on $CD8^+$ T cells.

Thirdly, we focused our in-depth analysis on $CD8^+$ T cells for two reasons: (1) ICIs primarily target pathways whose targets are predominantly expressed on $CD8^+$T cells, and (2) accumulating evidence supports a central role of $CD8^+$T cells in the pathogenesis of ICI-associated myocarditis. Within the $CD8^+$ T cell compartment, we identified six $CD8^+$ T cell subclusters and discovered a previously underappreciated $ISG15^+CD8^+$ T cell population. Notably, $ISG15^+CD8^+$ T cells were most prominently elevated in act-ICIAM patients but markedly decreased in no-ICIAM and rem-ICIAM groups. Based on this disease-associated expansion pattern, we considered $ISG15^+CD8^+$ T cells a plausible key immune population linked to active disease and therefore prioritized mechanistic interrogation of this subset. To experimentally address this, we isolated murine $CD8^+$ T cells and cocultured them with r-mIFN-α2 in vitro (**new Fig. 4D**). Notably, r-mIFN-α2 stimulation led to a marked increase in the proportion of $ISG15^+$ $CD8^+$ T cells (**new Fig. 4E**), supporting the notion that interferon signaling can directly induce ISG15 expression in $CD8^+$ T cells. Importantly, we further explored the functional consequences of this population. In cardiomyocyte coculture assays, $ISG15^+$ $CD8^+$ T cells significantly increased cardiomyocyte apoptosis (**new Fig. 4G**), elevated reactive oxygen species (ROS) production(**new Fig. 4H**), and enhanced expression of apoptosis markers and PUMA compared with controls (**new Fig. 4I**). Notably, these cytotoxic effects remained evident even at a lower ratio of 1:10 (**new Supplementary Fig. 6B-C**). These findings support a model in which $ISG15^+$ $CD8^+$ T cells are not merely passive markers of inflammation, but can actively contribute to myocardial damage.

Finally, in direct response to the reviewer's request, we expanded our analyses to other immune compartments, focusing specifically on monocytes because they showed the most prominent compositional decrease during active disease. Unbiased single-cell analyses revealed that monocytes displayed marked changes in both pathway activity and cellular composition during active disease, ranking second only to $CD8^+$ T cells. Through the single-cell profile of monocytes, we defined five transcriptionally distinct monocyte subpopulations, including $S100A12^{hi}CD14^+$monocytes, $HLA\text{-}DRB5^+CD14^+$ monocytes, IFN-activated (IFN^{act}) $CD14^+$ monocytes, $IL1B^{hi}$ $CD14^+$ monocytes, and

CD16^{+} monocytes (**new Supplementary Fig. 8A-B**). Notably, S100A12^{hi}CD14^{+}monocytes and IL1B^{hi} CD14^{+} monocytes were increased in act-ICIAM group (**new Supplementary Fig. 8C**), and DGE indicated that S100A12^{hi}CD14^{+}monocytes was the most influenced between no-ICIAM and act-ICIAM (**new Supplementary Fig. 8D-E**). Accordingly, we revised the Results section to present the global immune landscape first (Lines 216-226), followed by parallel pathway analyses across major cell types, before focusing on ISG15^{+}CD8^{+} T cells. And then we expanded our analyses to monocytes (Lines 425-466).

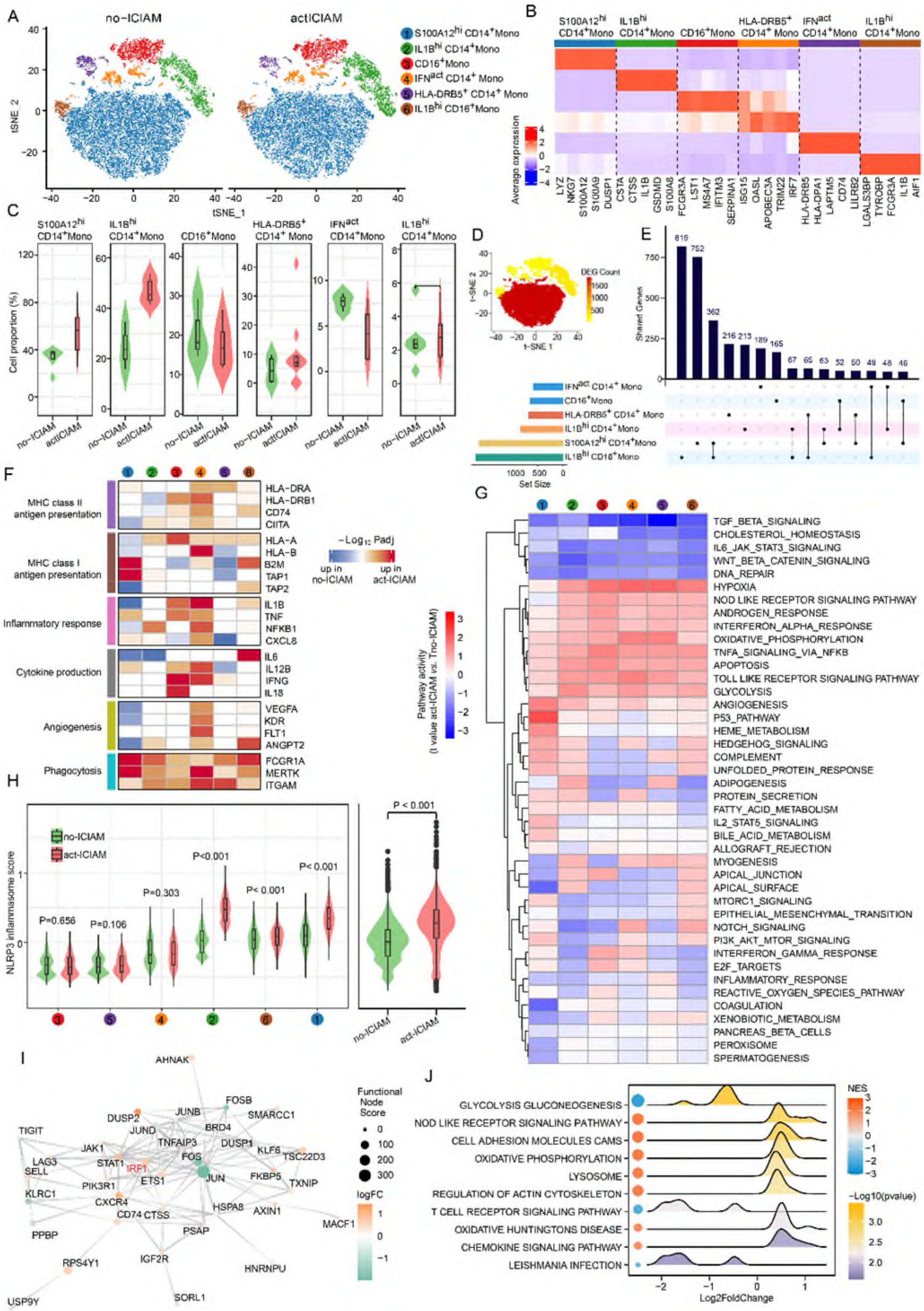

Supplementary Fig. 8. Changes in inflammatory response and cytokines in monocyte subclusters after treatment with different therapies.

A,Identification of 8 subclusters of monocytes between the no-ICIAM and act-ICIAM groups.

B, Heatmap showing the scaled expression of top 5 differentially expressed genes among 8 subclusters of monocytes.

C, Violin and box plot showing the proportion of each monocyte subcluster between the no-ICIAM and act-ICIAM groups.

D, Feature plot showing the count of DEGs determined by a two-sided Wilcoxon test.

E, UpSet plot showing all considered comparisons, with horizontal bars indicating the number of genes in each set and vertical bars showing the intersections, revealing shared DEGs across monocyte subsets.

F, Heatmap showing the relative change in the expression level of signature genes within each monocyte subcluster between the no-ICIAM and act-ICIAM groups.

G, Differences in pathway activities scored per scored per monocytes using GSVA, between the no-ICIAM and act-ICIAM groups. t values were independent of effects from the patient of origin.

H, Violin and box plot showing the JAK-STAT signaling pathway signature score across monocyte between the no-ICIAM and act-ICIAM groups. The signature score was calculated by the AUCell algorithm.

I, Protein-protein interaction graph depicting minimal significant interaction graph of DEGs in IL1B^{hi}CD14^{+} monocytes between the no-ICIAM and act-ICIAM groups (FDR $<1.0\times10^{-2}$).

J, Gene set enrichment analysis of the IL1B^{hi} CD14^{+} monocytes for inflammatory pathways comparing between the no-ICIAM and act-ICIAM groups.

Reviewer #4 myocarditis, cardio immune response (Remarks to the Author):

The manuscript presented by Zhang et al. is not only beautifully presented but also a simple tour de force including all elements of a study anybody could wish for: a range of state-of-the-art technology, clear patient relevance, several distinct animal models for mechanistic explanation, thorough validation. If anything, the immense amount of data could probably be broken up into several manuscripts to make them easier to digest. I did not find any concerns and congratulate the authors to this impressive achievement.

Noteworthy results are (among others) that the authors show that ISG15+CD8+ T cells are major culprits in ICI-myocarditis, and validating aspects of this using human cohorts as well as a range of distinct mouse models. Importantly, they use patient PBMC, allowing this study to have direct translational impact as blood biomarker.

This work will be of significance to the field as this is an analysis at thus far unseen scale and depth. The generated datasets will be of immense value to the community (provided they are being deposited as per FAIR data policy).

All conclusions and claims are robustly supported by several lines of evidence. The methodology is sound and exceeds standard expectations in the field. The methods are well described. I have not discovered any flaws.

Author Response: We sincerely thank the reviewer for the exceptionally positive and thoughtful evaluation of our work. We are deeply encouraged by the recognition of the comprehensiveness, translational relevance, and methodological rigor of our study. Thank you very much.

Minor comments:

1) I recommend to reword the title to take emphasis away from one selected technology (single-cell omics). The manuscript is so much more than just yet another single-cell atlas and that should be reflected in the title.

Author Response: We sincerely thank the reviewer for this insightful suggestion. We fully agree that our study extends well beyond a single-cell atlas and encompasses multi-dimensional approaches with significant translational relevance. In response, we have revised the title to better reflect the breadth and integrative nature of our work, while avoiding overemphasis on a single technology. The revised title is:Multi-omics profiling identifies distinct immune signatures with diagnostic and therapeutic implications in immune checkpoint inhibitor-induced myocarditis.

2) ICIAM feels like a rather clunky abbreviation for ICI-myocarditis. I suggest to spell out ICI-myocarditis for easier readability.

Author Response: We appreciate this helpful suggestion. We agree that the abbreviation “ICIAM” may affect readability. Therefore, we have replaced it with the full term “ICI-myocarditis” throughout the manuscript to improve clarity and accessibility for readers.

3) Data availability; in the interest of open science, I believe the presented datasets should be deposited in an appropriate repository.

Author Response: We fully agree with the reviewer on the importance of open science and data sharing. We have now deposited the key datasets generated in this study in a publicly accessible repository [PRJCA052032], and have included the corresponding accession numbers in the revised manuscript.

Reviewer #5 (Remarks to the Author):

I co-reviewed this manuscript with one of the reviewers who provided the listed reports. This is part of the Nature Communications initiative to facilitate training in peer review and to provide appropriate recognition for Early Career Researchers who co-review manuscripts.

Author response: Thanks so much for all the suggestions. These pieces of feedback have greatly improved the quality of our manuscript

Reviewer #1 (Remarks to the Author):

The revised manuscript is substantially improved, and I appreciate the authors' efforts to address the previous comments. Several important points have been strengthened, including the contextualization of ISG15 biology, clarification of corticosteroid status at sampling, additional monocyte analyses, reanalysis of the Blum et al. dataset, and the expanded discussion of JAK-STAT inhibition.

Author response: Thanks very much for the positive and constructive feedback on our manuscript. We appreciate the recognition of our work. Below, we provide a detailed point-by-point response to the concerns raised.

A few issues would still benefit from further refinement. First, the causal language regarding $ISG15^{+}$ $CD8^{+}$ T cells should be moderated. The new coculture and ISG15-knockdown experiments are valuable additions and strengthen the biological plausibility of this subset. However, because these experiments remain in vitro/ex vivo, they do not fully establish subset-specific pathogenic causality in vivo. I would therefore suggest replacing terms such as "direct pathogenic role" with more cautious wording, such as "pathogenic potential" or "capacity to induce cardiomyocyte injury in vitro."

Author response: We thank the reviewer for this valuable suggestion. We agree that our coculture and ISG15-knockdown experiments demonstrate the potential of $ISG15^{+}$ $CD8^{+}$ T cells to induce cardiomyocyte injury but do not fully establish subset-specific pathogenic causality in vivo. Therefore, we have revised the manuscript to replace stronger causal statements with more cautious wording, including "pathogenic potential" and "capacity to induce cardiomyocyte injury in vitro, " and clarified this limitation in the revised Results and Discussion sections.

Second, the diagnostic specificity of the 8-protein panel should be framed more cautiously. The panel appears promising for distinguishing active ICI-myocarditis from the tested ICI-treated non-myocarditis controls, but it has not yet been evaluated against other irAEs, non-ICI myocarditis, acute coronary syndromes, heart failure, infection, or other inflammatory conditions. Therefore, broader claims of ICI-myocarditis specificity should be avoided or clearly presented as requiring future validation.

Author response: We sincerely thank the reviewer for this important comment. In the revised manuscript, we have moderated our statements and clarified that this panel demonstrates potential utility for distinguishing active ICI-myocarditis from ICI-treated

patients without myocarditis in our cohort, rather than representing a definitive ICI-myocarditis-specific biomarker panel. We have also acknowledged that the current study did not evaluate the performance of this panel against other clinically relevant conditions, including non-ICI-related myocarditis, acute coronary syndromes, heart failure, infections, other immune-related adverse events, or systemic inflammatory disorders. Accordingly, we have added a statement in the Discussion emphasizing that further validation in larger, independent cohorts with broader clinical differential diagnoses will be required to determine the specificity and generalizability of this biomarker panel.

Finally, the manuscript would benefit from thorough professional English editing. Some typographical and grammatical issues remain, including "Basd on," "with high potential inducing cardiomyocyte apoptosis," "custom panel of 8-protein," "other ICIs-treated samples," "we used hallmark gene sets to to compare," "To be note," and "Shapley Additive exExplanations." Correcting these issues throughout the manuscript would improve clarity and readability.

Author Response: Thanks for the suggestion. We apologize for the typographical and grammatical errors in the previous version. We have thoroughly edited the entire manuscript and corrected all identified issues, including those specifically mentioned by the reviewer. In addition, we performed a comprehensive review of the manuscript to improve language accuracy, formatting consistency, and overall readability.

Overall, the manuscript has improved considerably, but further revision would help ensure that the conclusions are appropriately balanced and that the presentation meets publication standards.

Author Response: We sincerely thank you for the exceptionally positive and thoughtful evaluation of our work. We are deeply encouraged by the recognition of the comprehensiveness, translational relevance, and methodological rigor of our study. Thank you very much.

Reviewer #2 (Remarks to the Author):

I believe that the key points were answered, I have no further questions.

Author Response: We sincerely thank you for the careful re-evaluation of our revised manuscript. We greatly appreciate the reviewer's positive assessment and are pleased

that the major concerns have been adequately addressed. We are grateful for the constructive feedback provided during the review process, which has helped us further improve the quality and clarity of our manuscript.

Reviewer #3 (Remarks to the Author):

The authors responded very broadly and adequately to the reviewer's comments.

Author Response: We thank the reviewer for the constructive comments and kind evaluation. We greatly appreciate your recognition that our responses have adequately addressed the previous concerns. The suggestions provided during the review process were highly valuable and have contributed substantially to improving the rigor and presentation of our work.

Reviewer #4 (Remarks to the Author):

I maintain my prior assessment. This is a great manuscript. I would however like to reiterate the two points I made previously; please amend the data availability statement to reflect deposition as stated in the response to reviewers. Also ICIAM and ICI-myocarditis are now used interchangeably.

Author Response: We sincerely thank you for the positive assessment of our manuscript and for reiterating these important points. In response, we have amended the Data Availability Statement to accurately reflect the data deposition information as stated in our response to the reviewers. We have also carefully reviewed the manuscript and standardized the terminology throughout. Specifically, we now consistently use “ICI-myocarditis” throughout the manuscript to avoid interchangeable use of “ICIAM” and “ICI-myocarditis.”

We appreciate your helpful suggestions, which have improved the clarity and consistency of the manuscript.
